# Chalcogenide Ovonic Threshold Switching Selector

**DOI:** 10.1007/s40820-023-01289-x

**Published:** 2024-01-11

**Authors:** Zihao Zhao, Sergiu Clima, Daniele Garbin, Robin Degraeve, Geoffrey Pourtois, Zhitang Song, Min Zhu

**Affiliations:** 1 National Key Laboratory of Materials for Integrated Circuits, Shanghai Institute of Microsystem and Information Technology, Chinese Academy of Sciences, Shanghai, 200050 People’s Republic of China; 2 University of Chinese Academy of Sciences, Beijing, 100029 People’s Republic of China; 3 IMEC, Kapedreef 75, Leuven, Belgium

**Keywords:** Non-volatile memory, Ovonic threshold switch (OTS), Chalcogenide, Selector

## Abstract

Today’s explosion of data urgently requires memory technologies capable of storing large volumes of data in shorter time frames, a feat unattainable with Flash or DRAM. Intel Optane, commonly referred to as three-dimensional phase change memory, stands out as one of the most promising candidates. The Optane with cross-point architecture is constructed through layering a storage element and a selector known as the ovonic threshold switch (OTS). The OTS device, which employs chalcogenide film, has thereby gathered increased attention in recent years. In this paper, we begin by providing a brief introduction to the discovery process of the OTS phenomenon. Subsequently, we summarize the key electrical parameters of OTS devices and delve into recent explorations of OTS materials, which are categorized as Se-based, Te-based, and S-based material systems. Furthermore, we discuss various models for the OTS switching mechanism, including field-induced nucleation model, as well as several carrier injection models. Additionally, we review the progress and innovations in OTS mechanism research. Finally, we highlight the successful application of OTS devices in three-dimensional high-density memory and offer insights into their promising performance and extensive prospects in emerging applications, such as self-selecting memory and neuromorphic computing.

## Highlights

- The development history and key milestones of ovonic threshold switch (OTS) materials were comprehensively summarized. Combined with the latest advancements of OTS research, the mainstream OTS material systems were systematically introduced.
- A thorough overview of the prevailing viewpoints regarding the OTS switching mechanisms was presented.
- Recent progress in OTS devices for applications in 3D memory, self-selecting memory, and neuromorphic computing was summarized.

## Graphical abstract

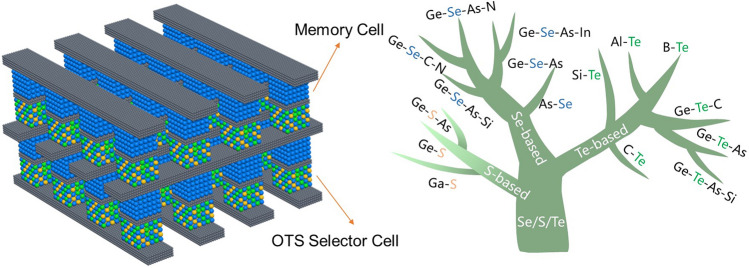

## Introduction

**Fig. 1:**
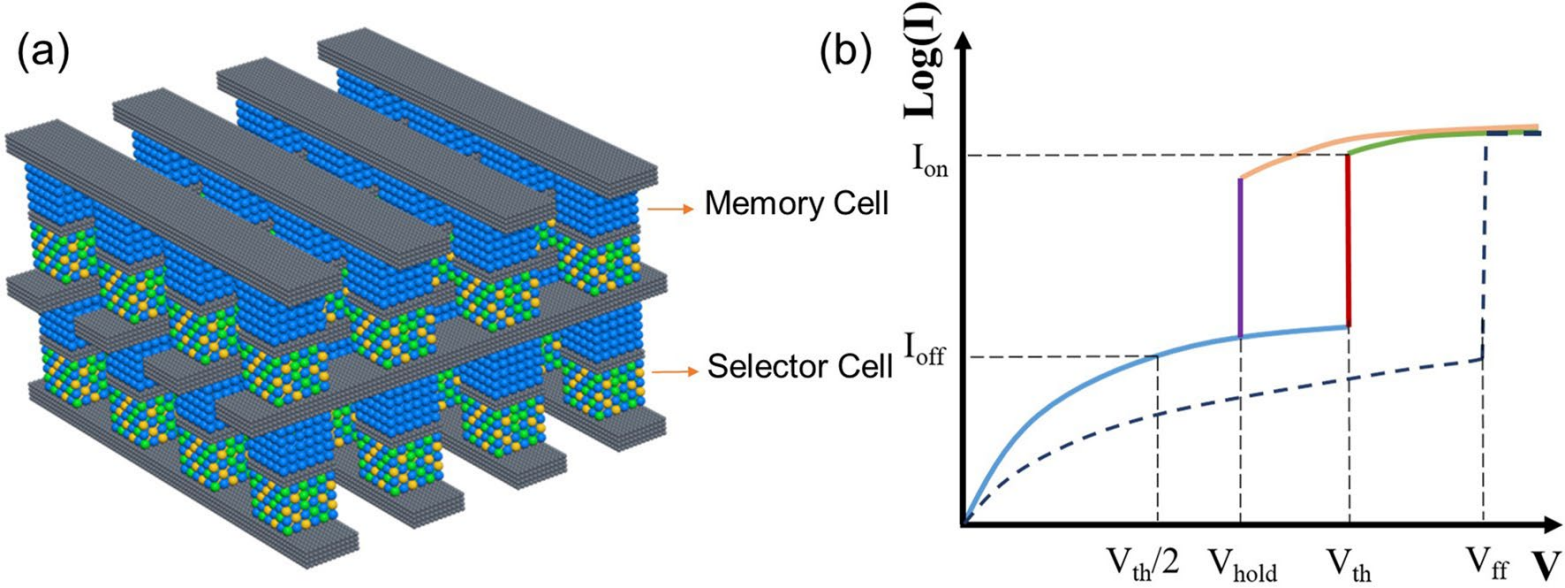
3D Crosspoint architecture (**a**) and typical current–voltage (*I*-*V*) curve of the selector cell (**b**). A first fire voltage (*V*_ff_) is required to operate the selector cell. Then, a relative low voltage, called threshold voltage (*V*_th_), can turn on the cell, which would return to the off-state as the voltage decreases to the hold voltage (*V*_hold_). *V*_ff_ > *V*_th_ > *V*_hold_

The era of big data and AIoT (Artificial Intelligence and Internet of Things) necessitates the novel memory technologies for massive and quick integration of data storage, which is predicted to reach 175 ZB (1 zettabyte = 10^21^ byte) in 2025 [1]. The 3D stackable emerging non-volatile memory has become a key solution to address the challenge. In 3D high-density stacking, as process nodes continue to shrink from hundreds of nanometers to a few nanometers, the spacing between the parallel memory cells in the stack decreases, resulting in an increased load on the metal interconnects (Fig. 1a). “Leakage current” or “sneak current” becomes an unavoidable issue. Leakage current can cause crosstalk between neighboring memory cells, affecting read and write operation, interfering with stored data, reducing storage lifespan, and increasing power consumption [2–4]. To effectively suppress leakage current, it is essential to control all possible leakage paths. The most efficient solution is to directly connect each memory cell to an independent device called “selector,” forming the memory array.

The selector is not simply a dielectric layer material or a high-resistance device; it appears to be simple but has several requirements. From a functional perspective, the selector must be an independent device with switch function (Fig. 1b). Considering high integration density, simple two-terminal structures, capable of vertically stacking with the memory cell, would significantly increase the spatial utilization [2]. Additionally, the selector needs to accurately control the selected storage unit when activated, strictly prevent the selection of other storage units, and maximize the suppression of leakage current in these unselected cells. The selector thereby should exhibit low leakage current in the off-state, while providing sufficient high drive current in the on-state. Furthermore, as the most common operation in memory, that is, read/write operation, needs to switch on/off the selector cell, the selector should have high stability and endurance [5]. From the perspective of the memory system, the performance of the selector should be compatible with the operation logic of the peripheral circuitry, necessitating high operational speed (switching speed) to accommodate high-speed read and write operation. Moreover, it is preferable for the selector to have bi-directional select capability to adapt to different operation rules. From a manufacturing process standpoint, the selector should have good compatibility with CMOS technology and not damage the memory cells during fabrication. Therefore, the research on selector is of great importance and has even become one of the core issues in 3D high-density memory.

**Fig. 2:**
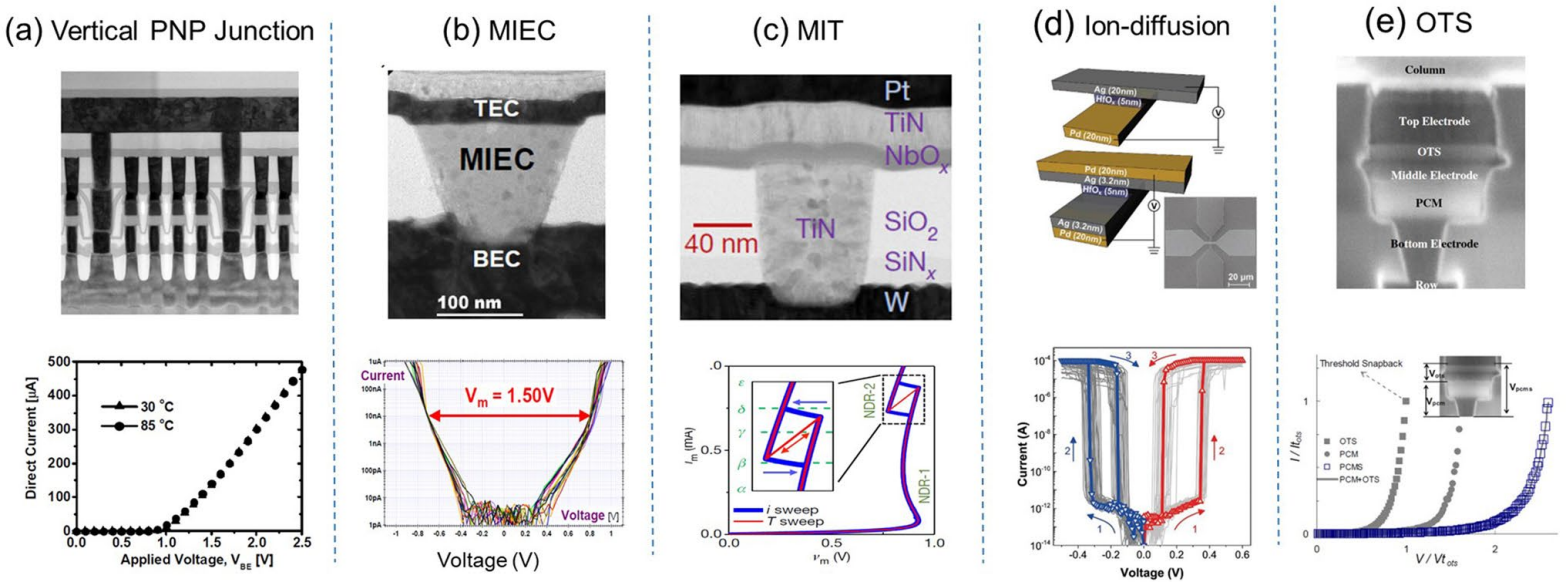
Various selector candidates. **a** Vertical PNP junctions. Adapted with permission from [6]. Copyright 2009, IEEE. **b** Mixed ion–electron conductor (MIEC). Adapted with permission from [7]. Copyright 2012, IEEE. **c** Metal–insulator transition (MIT). Adapted with permission from [9]. Copyright 2017, Springer Nature. **d** Ion-diffusion threshold switching. Adapted with permission from [10]. Copyright 2017, John Wiley and Sons. **e** Ovonic threshold switching (OTS). Adapted with permission from [11]. Copyright 2009, IEEE

After decades of effort, many selector candidates have been proposed (Fig. 2), such as traditional PNP junctions [6], mixed ion–electron conductor (MIEC) [7], metal–insulator transition (MIT) [8, 9], ion-diffusion threshold switching [10] as well as ovonic threshold switch (OTS) [11]. Among them, chalcogenide-based OTS selector, which exhibits distinct high drive current, nanosecond speed, low leakage current, and excellent scalability, is the only one that has been successfully used in commercial 3D memory chips, namely Optane by Intel [12]. In this paper, we first briefly introduce the discovery process of the OTS phenomenon. Next, we summarize the key electrical parameters of OTS devices and discuss the recent explorations of OTS materials, which are classified as Se-based, Te-based, and S-based material systems. Furthermore, we address the various theoretical explanations for the OTS switching behavior, listing the diverse perspectives and reviewing the progress and innovations in OTS mechanism research. Finally, we highlight the successful application of OTS devices in three-dimensional high-density memory, and we provide insights into their promising performance and extensive prospects in emerging applications such as self-selecting memory and neuromorphic computing.

## Discovery of OTS Phenomenon

In 1964, Northover and Pearson from Bell Telephone Laboratories first observed the threshold switching (TS) phenomenon in As–Te–I [13]. This phenomenon refers to a rapid increase in electrical conductivity and a transition to a low-resistance state (on-state) when a specific threshold voltage (*V*_th_) is reached under applied external voltage. Dennard also found this phenomenon in As–Te–Se [14]. In 1966, S.R. Ovshinsky applied for a patent [15], in which he reported his findings on reversible switching in memory devices composed of 48 at.% tellurium, 30 at.% arsenic, 12 at.% silicon, and 10 at.% germanium (amorphous Te_48_As_30_Si_12_Ge_10_). Then, in his paper published in 1968 [16], he notes at the end that “an unusual memory effect is observed in materials in which structural changes are facilitated by the removal of cross-linking elements from the above formula—for example, the reduction of arsenic to 5 at.%. After switching from a highly resistive state, structural changes result in the preservation of a conductive state even when the current is totally removed. The material can be reversibly switched back to the highly resistive state by application of a current pulse of either polarity exceeding a threshold value.”

From a modern perspective, these descriptions imply three key points. Firstly, chalcogenide devices exhibiting the TS phenomenon can display volatile (returning to the initial low-conductivity state after removing external excitation) or non-volatile (maintaining a high-conductivity state after removing external excitation) electrical behaviors, which are later known as OTS and ovonic memory switch (OMS), respectively. Secondly, the same material system can exhibit memory potential by modifying the composition (As < 5 at.%), establishing a basis for future composition optimization and selector/memory applications. Thirdly, a short and high-amplitude current pulse can be applied to achieve the state transition in OMS devices, which laid the foundation for the future RESET operation in phase change memory (PCM), enabling controllable and reversible phase change operation.

**Fig. 3:**
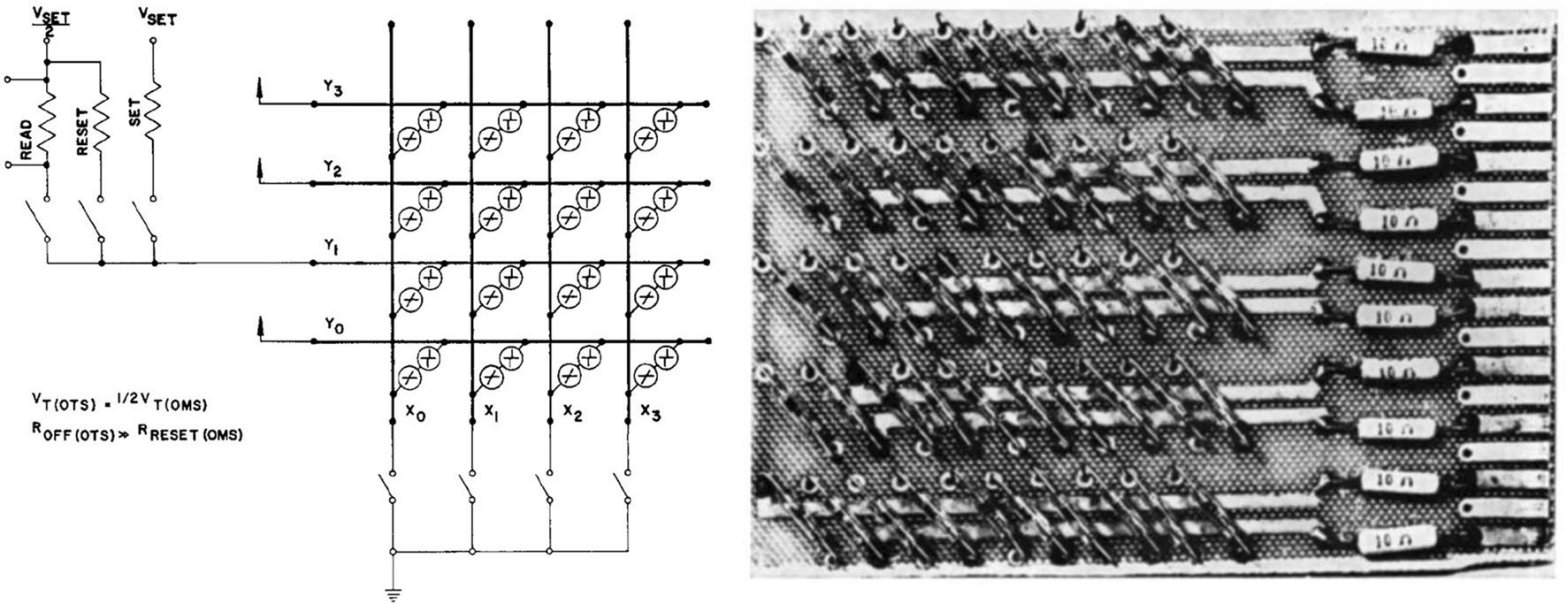
Schematic diagram and photograph of an OTS + OMS array. Adapted with permission from [17]. Copyright 1970, Elsevier

The concept of OTS itself represents a categorization of specific electrical performance in materials rather than a physical definition. Broadly speaking, material systems that exhibit the OTS phenomenon and meet specific criteria can be referred to as OTS materials. The distinction between OTS and OMS (also known as PCM) material systems, as well as the analysis of specific parameters, remains a focal point in chalcogenide research. In 1970, D.L. Nelson from Ovshinsky’s company (Energy Conversion Devices) explicitly delineated the electrical characteristic differences between OTS and OMS and successfully fabricated the first storage array with independent OTS and OMS structures, as displayed in Fig. 3, a precursor to the 1S1R (S: selector; R: resistance/memory) structure for today’s 3D memory architecture [17]. Although chalcogenide TS devices demonstrated typical switching performance, their goal of replacing Si-based and Ge-based transistors was not successfully achieved in practice at that time. However, non-volatile chalcogenide TS devices, later known as PCM, gradually gained widespread application in the field of optical storage after the discovery of GeTe–Sb_2_Te_3_ pseudo-binary alloys [18, 19] and subsequent Ag–In–Sb–Te materials [20, 21].

Although both optical and electrical differences between the crystalline and amorphous states of PCM were discovered, the development of electrical storage using PCM progressed slowly due to limitations imposed by the semiconductor fabrication technology at that time [22]. It was not until the early twenty-first century, with the scaling down of integrated circuit process nodes to 180 nm, that PCM gained significant attention again due to its fast switching speed [23, 24], large resistance contrast, and excellent scalability [20]. Since 2001, major storage companies worldwide, including Intel, Samsung, IBM, Micron, and STMicroelectronics, have conducted research on PCM and subsequently introduced their storage product, in which traditional transistors were applied as the selector cell, including MOSFETs, PN diodes and vertical PNP junctions [6]. Although 4 Mb to 8 Gb PCM chips were fabricated [21, 24–27], the fact that the transistor cannot survive without the silicon substrate makes it impossible to achieve multiple-desks stacking for 3D high-density memory. Noticeably, in 2009, Intel and Numonyx proposed a novel scalable and stackable memory [11], built by layering an OMS and an OTS, which was a crucial milestone in the ultimate commercialization of 128 Gb PCM-based 3D Xpoint in 2017. Since then, OTS selectors have emerged as the most promising selector in the new generation of 3D stacked memories, and more and more novel OTS materials were explored.

## OTS Materials and Device

OTS materials, discovered during the same period as PCM, are primarily composed of chalcogenide. Besides the slight difference in stoichiometry, OTS materials are only found in the amorphous state, whereas crystalline phase are still needed for the PCM [23]. Therefore, a stable amorphous system, which can withstand 400–450 °C annealing for 30 min in the back-end-of-line process [29], is often sought in the design of OTS materials. The OTS devices have a sandwich structure, and exhibit two states: on and off states. As shown in Fig. 1b, a first fire voltage (*V*_ff_) is required to operate the selector cell. Then a rather low voltage, called threshold voltage (*V*_th_), can turn on the cell (on-state), which could return to the off-state as the voltage decreases to the hold voltage (*V*_hold_). Therefore, *V*_ff_ > *V*_th_ > *V*_hold_. Specific electrical parameters of OTS devices are: (1) on-state current (*I*_on_)/current density (*J*_on_), (2) off-state leakage current (*I*_off_), (3) on/off ratio or selectivity, (4) endurance, (5) thermal stability, (6) threshold voltage/field (*V*_th_/*E*_th_), and (7) switching speed (*t*_on_/*t*_off_). Appropriate control of these electrical parameters can greatly satisfy the application of OTS in storage devices, as well as its application in other fields.

For 3D high-density memory applications, there are several requirements for the OTS device, which exhibit significant correspondences to material properties, as summarized in Table 1. Firstly, in order to successfully drive the connected memory cell, the OTS cell requires a large driving current density, for example, greater than 10 MA cm^−2^ for 3D PCM [28, 29]. This necessitates OTS materials with saturated covalent bond to provide a more robust amorphous network, thus tolerating high passing *I*_on_ current without abundantly breaking the bonds and recrystallization. Secondly, to achieve > Mb-scale capacity storage, the leakage current (*I*_off_) of the OTS device, obtained at *V*_th_/2, should be suppressed below 10^−8^A and a selectivity (*I*_on_/*I*_off_) of at least 10^4^ is needed [4, 30]. This requires materials with a big mobility gap and thereby high activity barrier [31, 32]. Thirdly, as the most frequent read operation in practical 3D memory needs to switch on/off the OTS selector, the endurance of the OTS device should be several orders of magnitude higher than the one of the memory unit (typically 10^6^ cycles for PCM and resistance random access memory RRAM [20, 33]), that is, exceeding 10^8^ cycles for the OTS cell. This necessitates OTS materials with high thermal stability (to avoid crystallization-caused failure) and no phase separation during operation. Fourthly, the amorphous state of OTS device should be able to withstand a temperature of 400–450 °C in the back-end-of-line (BEOL) process since the OTS behavior disappears upon crystallization, in which the insulator and contact metal layers are deposited [29, 34]. This requires the crystallization temperature of the amorphous OTS material to be higher than 400 °C. Fifthly, to be compatible with advanced logic applications (e.g., 3.3 V for *I*/*O* pin) and also reduce the power consumption, an OTS cell with a low *V*_th_ are recommended [35]. This implies that the material's mobility gap and trap state density should not be too large. Obviously, this requirement is contrary to that of low *I*_off_; therefore, a comprehensive consideration is needed. To achieve high-speed storage, the switching speed of the OTS device should be within 100 ns, faster or comparable with the memory units. This requires the OTS material to be chalcogenides without Ag, Cu, etc., active elements, avoiding atomic diffusion and maintaining an electron-dominated switching process. In addition, the drift of *V*_th_ (i.e., the change of *V*_th_ over time after the operation) in OTS materials is also a noteworthy issue; *V*_th_ drift should be as small as possible. It should be noted that the OTS device structure (such as OTS material layer thickness, multi-layer structure [36]) and operation mode [37] can also affect the overall device performance.

**Table 1:** The application of 3D memory requires OTS materials to meet many requirements

Key parameters	Application requirement	Material requirement
On-state current (*I*_on_)/current density (*J*_on_)	> 10 MA cm^−2^ for PCM applications	Saturated covalent bond
Off-state leakage current (*I*_off_)	< 10^–8^ A	Big mobility gap
*I*_on_/*I*_off_ or selectivity	> 10^4^ for 1 Mb memory array	Strong bond and big mobility gap
Endurance	> 10^8^ cycles	High crystallization temperature and no phase segregation
Thermal stability	400–450 °C annealing for 30 min	> 400 °C crystallization temperature
Threshold voltage (*V*_th_)	< 3.3 V	Relatively low mobility gap and trap state density
Switching speed (*t*_on_/*t*_off_)	< 100 ns	Inert material to avoid the diffusion

The development of OTS material systems is not confined to a single pathway. Specifically, the research on OTS material compositions generally exhibits a trend from simplicity to complexity. Typically, the development starts with a simple basic material with good performance, which is then analyzed and optimized to address any deficiencies. Through doping and compositional optimization, a multi-component material system is eventually established. The reasons for this development trend are multifaceted [29]. Firstly, in the research and development of OTS switch devices, optimization of specific components is often carried out for engineering considerations or the need for mechanism studies to enhance certain performance indicators. Secondly, the successful application of OTS devices in commercial products is exciting, but the process of commercialization introduces additional considerations such as cost, process compatibility, and environmental impact. These new requirements drive innovation and optimization of OTS materials. Lastly, the optimization and innovation of a material system are often propelled by the advancement of its mechanism research. Different theoretical analyses and explanations of OTS switching mechanisms lead to variations, and the optimization of performance under different theories will employ different compositional optimization methods.

**Fig. 4:**
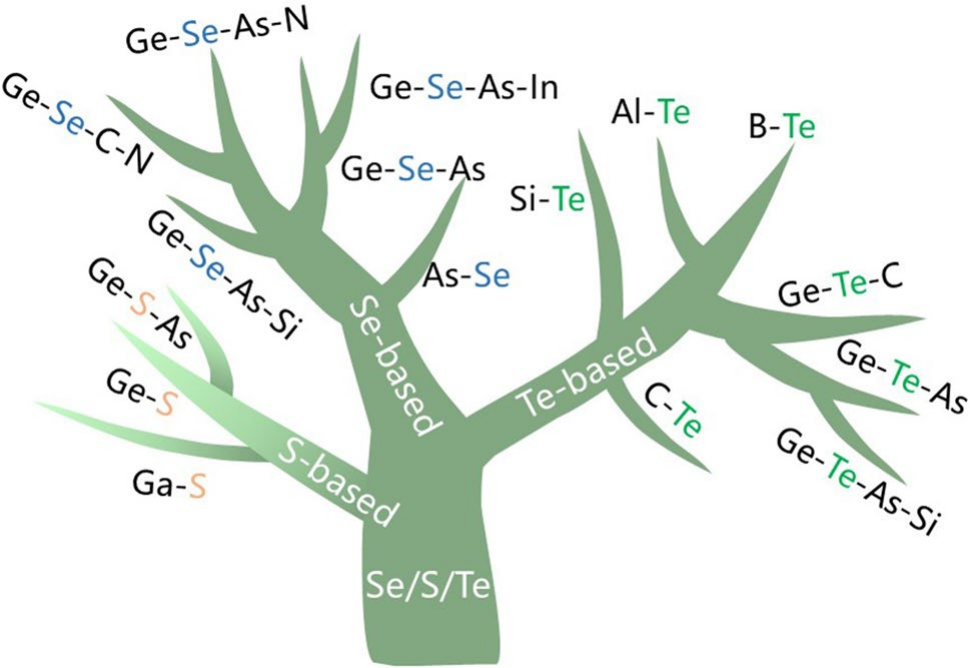
OTS material tree. The tree depicts three material systems, including Te-, Se-, and S-based ones

Doping processes and compositional optimization, as typical means of material performance enhancement, are also applicable to OTS devices. It is evident that the introduction of a new element usually affects more than one key parameter, regardless of the initial purpose of its introduction. From a material design perspective, the incorporation of elements such as Ge, As, Si, C, N, and B can improve the material’s crystallization temperature, stabilize the amorphous system, or reduce leakage by introducing elements with wider mobility gap. Subsequently, targeted improvements can be made based on the defects in these binary compounds. The participation of these elements, with varying relative concentrations, often nonlinearly affects electrical parameters such as *V*_th_ and *I*_off_. Therefore, the composition of OTS materials is not a simple superposition of fixed doping elements. On the contrary, these elements interact with each other in a multi-component system, and the addition of some elements may even cause the system to lose its OTS characteristics. Although dozens of OTS materials have been explored since 1966, Se, Te, and S chalcogens are essential elements, as summarized in Fig. 4, which are discussed in detail as follows.

### Se-based OTS

**Fig. 5:**
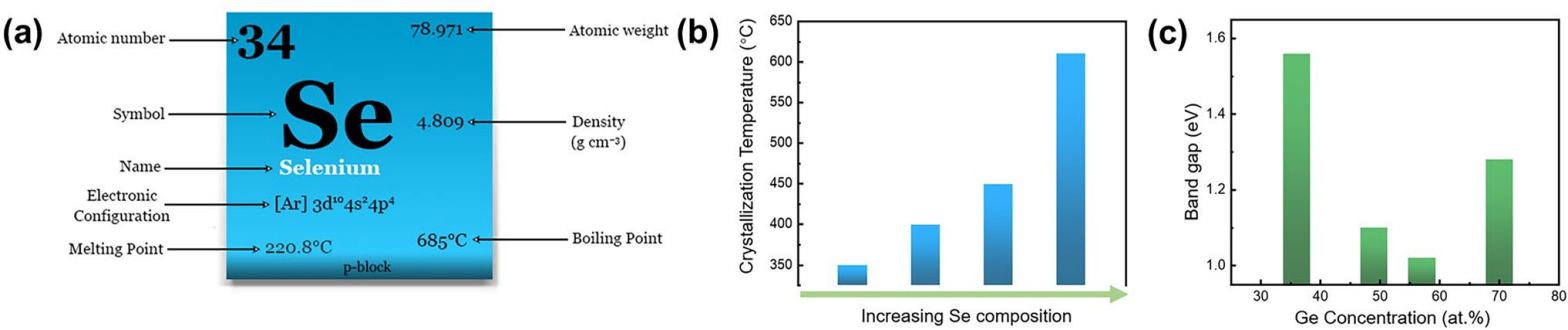
Elemental Se and Ge–Se properties. **a** Element selenium’s properties. **b** Crystallization temperatures (*T*c) [38] and **c** mobility gap of Ge–Se alloys [39, 40]. Adapted with permission from [39]. Copyright 2011, IOP Publishing

**Fig. 6:**
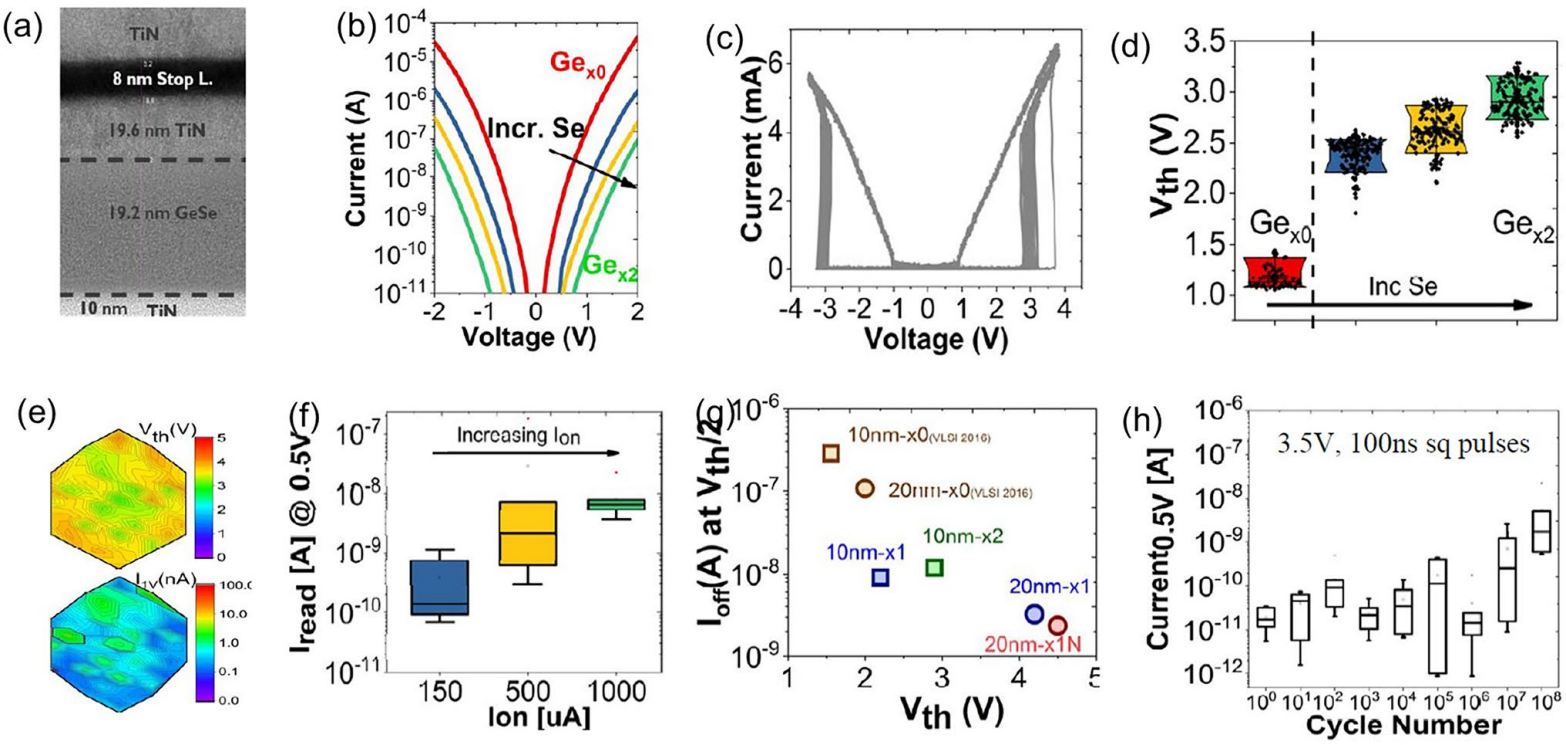
Ge–Se OTS selectors. **a** Device structure with 50-nm plug. **b** Leakage current. **c** AC *I*–*V* curves. **d** *V*_th_ increase with higher Se content. **e** *V*_th_ and *I*_off_ distributions. **f** *I*_on_ vs. leakage current. **g** *I*_off_ comparison and **h** endurance. Adapted with permission from [38]. Copyright 2018, IEEE

Selenium (Se) is the number 34 element in the periodic table (Fig. 5a), with an atomic radius of 1.20 Å. Se-based OTS materials, in which the Se element has the highest concentration, often combine with Ge to form Ge–Se alloys. Ge–Se compounds have been widely reported as OTS matrix for mechanism analysis, doping, and compositional optimization [41–47]. Ge–Se exhibits a high crystallization temperature, with GeSe having a crystallization temperature of 350 °C (Fig. 5b) and further rising up to 600 °C with higher Se content [38, 39]. Since elemental Se has a large mobility gap of ~ 2 eV, the Ge–Se-based materials have a mobility gap ranging from approximately 1–2 eV (Fig. 5c) [46], demonstrating < 10^−7^A low leakage current and > 10^8^-cycle high endurance (Fig. 6b, h). The trap states of Ge–Se alloy are located at 0.42–0.56 eV below the conduction band edge using a Poole–Frenkel fitting [38]. The Ge–Se bond has a short bond length of ~ 2.38 Å and thereby high bond energy of 234.5 kJ mol^−1^ [32]. Thus, Se-based OTS materials generally exhibited large *V*_th_, > 2 V@10 nm thick, which would further increase with higher Se concentration (Fig. 6d) [38].

**Fig. 7:**
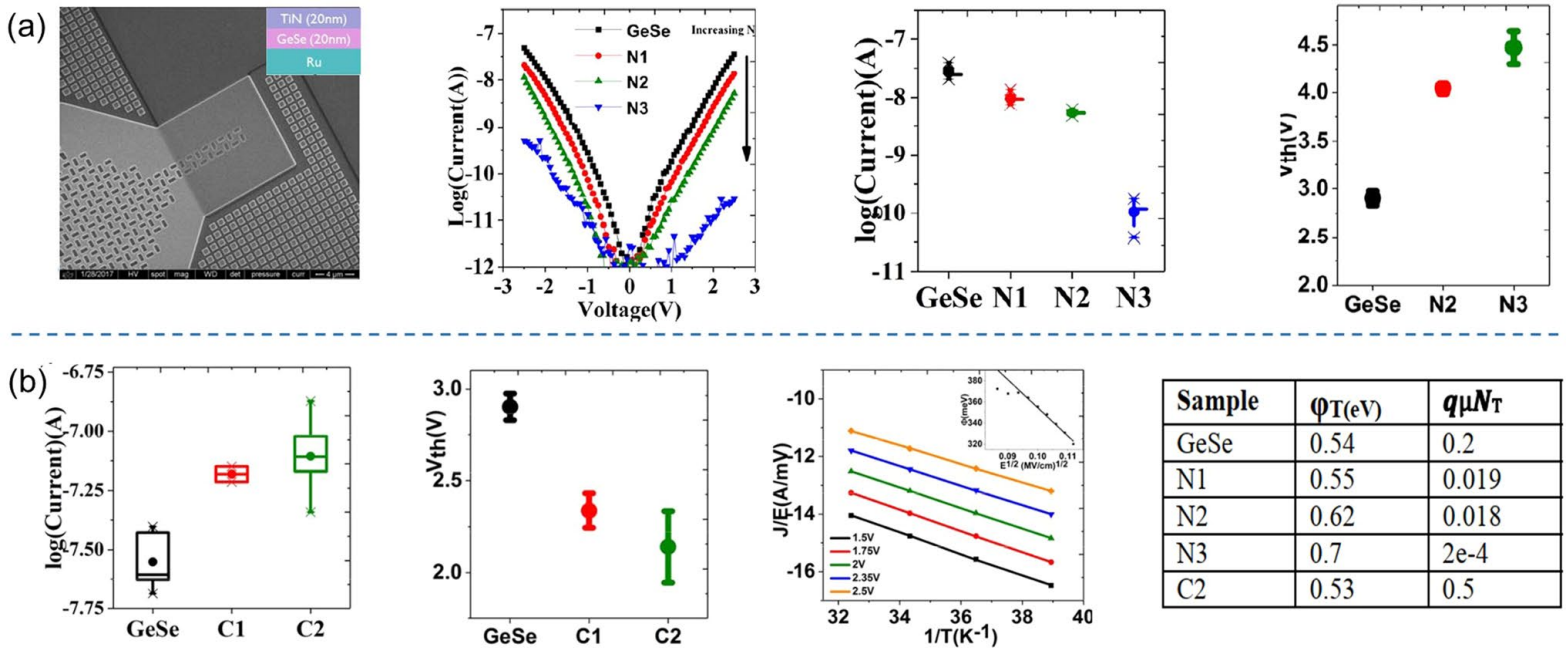
N- or C-doped GeSe OTS selectors. **a** Device structure with 3 μm × 3 μm size; *I*–*V* curves of N-doped GeSe selector; *I*_off_ vs. different N contents; *V*_th_ vs. different N contents. **b** *I*_off_ vs. different C contents; *V*_th_ of C-doped samples; T dependence of subthreshold conduction fitting confirming Poole–Frenkel conduction; trap positions and densities. Adapted with permission from [32]. Copyright 2017, IEEE

When optimizing the Ge–Se system, the overall objective is to construct a stable amorphous network, enhance thermal stability, reduce leakage current, and, to some extent, lower the *V*_th_. Researchers have extensively investigated the improvement of Ge–Se system performance through doping engineering. For instance, Avasarala et al*.* introduced N into Ge–Se to eliminate some of the Ge dangling bonds, resulting in a shift of the mobility edge, hence increased mobility gap, and reduced *I*_off_ (Fig. 7a) [32]. As the content of N increased from N1 to N3, the trap states became deeper (0.54–0.7 eV), reducing defect density and minimizing leakage current from ~ 5 × 10^–8^ A to ~ 10^–10^ A, yet causing a sharp increase in *V*_th_ from ~ 2.8 eV to ~ 4.5 V (Fig. 7a). Moreover, N doping effectively enhanced the thermal stability of the material (> 450 °C). On the other hand, C doping in Ge–Se led to an increase in *I*_off_ up to ~ 10^–7^ A and a ~ 0.8 V decrease in *V*_th_ as the content of C reached C2 (Fig. 7b), which differ from N doping. Avasarala et al. believed that the increased density of trap states or introduced C-chain-based gap states after C doping was responsible for the increased leakage and the decreased *V*_th_ [32].

**Fig. 8:**
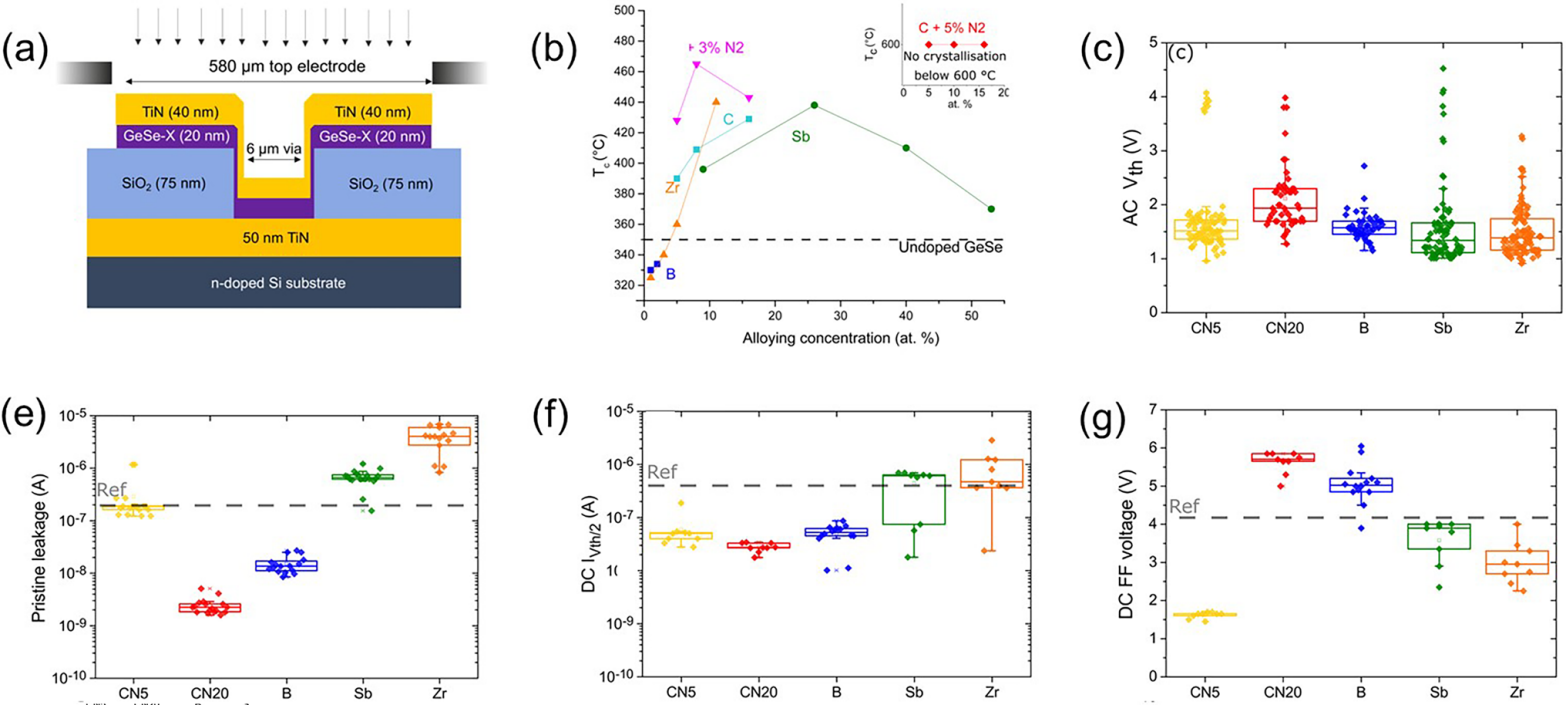
C/Sb/B/N/Zr-doped GeSe OTS selectors. **a** Device structure with 6 um size. **b** Crystallization temperature *T*_c_. **c** Alternating current (AC) *V*_th_. **e** Pristine leakage at 1 V/2 V. **f** *I*_off_. **g*** V*_ff_ voltage. Adapted with permission from [49]. Copyright 2021, AIP Publishing

Similarly, Sb doping decreases *V*_th_, and the higher the Sb content (Sb < 22 at.%), the lower the *V*_th_ (Fig. 8) [48]. In 2021, Keukelier et al*.* conducted a systematic study on the effects of doping elements on the thermal stability and OTS performance of the Ge–Se system, focusing on Zr (metal), B, Sb (metal-like), C, and N (non-metal) [49]. Their results showed that B doping lowered the crystallization temperature, while Zr doping initially lowered it and then surpassed the pristine material temperature at 5 at.% (Fig. 8b). Sb doping initially increased the crystallization temperature, but started to decrease after reaching 26 at.%. C doping significantly raised the crystallization temperature, with a temperature exceeding 400 °C at 8 at.%. Further N doping increased the crystallization temperature to 600 °C (Fig. 8b). No matter metallic or metalloid elements are doped, the alternating current (AC) *V*_th_ of the sample had little difference and remained about 1.5 V (Fig. 8c). *C*/*N* and B doping reduced leakage current, while Sb and Zr doping increased it (Fig. 8e, f). In Ge–Se, Ge–Ge homopolar bonds contributed to leakage current, and N doping reduced homogenization and leakage current. Doping with only N led to excessively high operating voltages, while doping with only C increased leakage current. The most suitable approach was believed to be C and N co-doping. Overall, Sb and Zr contents above 10 at.% modulated the crystallization temperature to exceed 400 °C. In the study, metal and metalloid elements even decreased the crystallization temperature at low doping concentrations (< 3 at.%), while the non-metal element C effectively increased the crystallization temperature at relatively low doping concentrations. Regarding the impact on electrical characteristics, metal elements were more effective in reducing the *V*_th_ and *V*_ff_ (Fig. 8g).

**Fig. 9:**
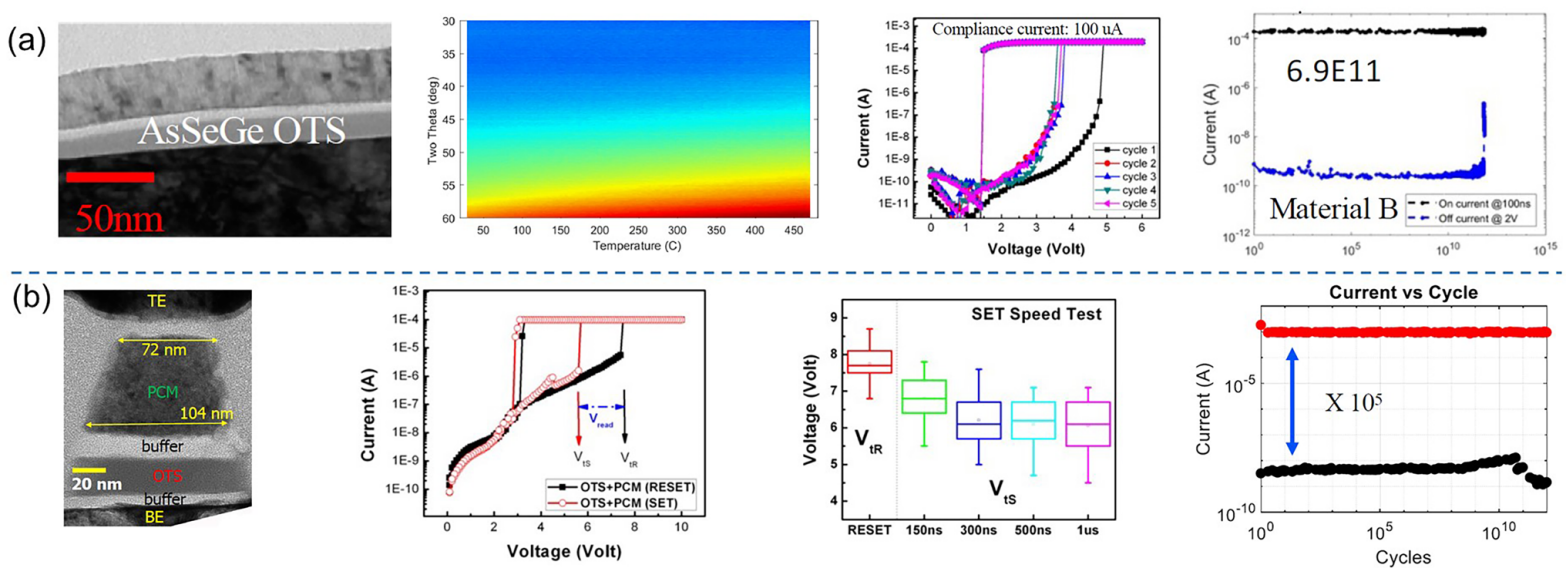
Ge–Se–As OTS and 1S1R cells. **a** OTS device with 350-nm plug; > 450 °C thermal stability; *I*–*V* curves showing 3.5 V *V*_th_ and ultralow *I*_off_ (131 pA@2 V); 6.9 × 10^11^-cycle endurance. **b** 1S1R stacking structure; *I*–*V* curves showing ~ 2 V memory window; 300 ns speed and > 10^12^-cycle read endurance. Adapted with permission from [50]. Copyright 2021, IEEE

The element As, despite being considered environmentally unfriendly and requiring specific fabrication processes, plays a significant role in the optimization of Ge–Se OTS materials. Most importantly, Ge–Se–As–Si–based OTS material was believed to be the one used in the commercial 3D Xpoint in 2017 [11]. The IBM/Macronix team, focusing on PCM, conducted continuous research on the Ge–Se–As system [50–52]. Cheng et al*.*, by optimizing the content of Ge and As, successfully increased the materials’ crystallization temperature to over 450 °C and achieved a low* I*_off_ of 10^–10^ A, a large *J*_on_ of 7.9 MA cm^−2^ and the device endurance exceeding 6.9 × 10^11^ cycles (Fig. 9a) [50]. Using this selector, 1S1R (OTS + PCM) structure was successfully fabricated, demonstrating a ~ 2 V memory window, a 300 ns speed and > 10^12^-cycle read endurance (Fig. 9b). Subsequently, the team further improved the Ge–As–Se system by doping. The introduction of Si in Ge–Se–As devices extended the endurance to 10^11^, while doping Te resulted in Ge–Se–As–Si devices with enhanced thermal stability up to 500 °C and endurance reaching 10^11^. In 2021, they also addressed the trade-off issue among *V*_th_, *I*_off_, and cycling endurance in B-, C-, and S-doped OTS selectors. Furthermore, In-doped Ge–Se–As OTS devices exhibited low *I*_off_ (~ 0.1 nA), high endurance (~ 10^10^ cycles), and inhibition of *V*_th_ drift, while minimally affecting *V*_th_ and *I*_off_ [52].

In terms of fabrication techniques, in 2023, Jun et al*.* employed the atomic layer deposition (ALD) process to fabricate Ge–Se–S-based OTS devices [53]. The novel Ge–Se–S material exhibited a slightly larger *V*_th_ drift compared to GeSe_2_. However, it demonstrated several advantages, including a lower *I*_off_, and a smaller *V*_th_ fluctuation up to 10^6^ cycles. The successful application of the ALD process in the fabrication of Ge–Se system OTS devices is expected to contribute to the further miniaturization of 3D cross-point (Xpoint) memory and the realization of 3D Vertical memory in future.

Table 2 summarizes device performances of Ge–Se-based OTS selectors. Clearly, Ge–Se-based OTS selectors present thermal stability of > 350 °C, *J*_on_ ranging from 0.1 to 23 MA cm^−2^, *I*_off_ ranging from 10^–7^ to 10^–10^ A, *V*_th_ ranging from 1.4 to 4 V, *V*_hold_ ranging from 0.5 to 1.8 V, endurance ranging from 10^6^ to 10^11^ cycles.

**Table 2:** Performance summary and comparison of Se-based OTS devices

Materials	Feature size/nm	Selectivity	*J*_on_/MA cm^−2^	*I*_off_/A	*V*_th_/V	*V*_hold_/V	Speed/ns	Endurance	Thermal stability/°C
GeSe [54]	50	10^3^	23	10^–7^	~ 1.4	~ 0.5	~ 2	10^8^	~ 350
Ge–Se–N [32]	50	10^5^	23	2 × 10^–9^	~ 4	~ 1	–	10^8^	> 475
Ge–Se–Si [43]	200	10^3^	1.6	10^–7^	~ 2.4	~ 1.2	~ 20	5 × 10^6^	> 375
Ge–Se–As [50]	350	10^5^	7.9	1.3 × 10^–10^	~ 3.5	~ 1.2	~ 10	6 × 10^11^	~ 450
Ge–Se–Sb–N [31]	–	10^4^	1.4	10^–9^	2.2	0.76	–	10^6^	< 475
Ge–Se–As–Te–Si [55]	350	10^4^	0.44	1.9 × 10^–9^	~ 2.2	~ 1.5	~ 50	> 10^10^	> 350
Ge–Se–As–In [52]	350	10^6^	0.1	~ 1 × 10^–10^	3.7	~ 1.8	–	< 10^10^	~ 350
Ge–Se–S [53]	50	10^4^	~ 5	4 × 10^–8^	~ 3.2	–	–	> 10^6^	–

### Te-based OTS

**Fig. 10:**
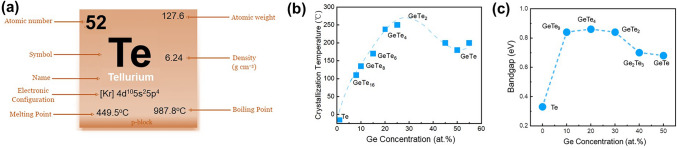
Elemental Te and Ge–Te properties. **a** Element tellurium’s properties. **b** Crystallization temperatures (*T*c) [56] (Adapted with permission from [56]. Copyright 1986, AIP Publishing) and **c** the mobility gap [57] (Adapted with permission from [57]. Copyright 1974, Elsevier) of Ge–Te alloys, respectively

Tellurium (Te) is the 52nd element on the periodic table, with an atomic radius of 1.4 Å (Fig. 10). Te exists in the trigonal crystalline state at room temperature since it has an ultralow crystallization temperature of −10 °C [58]. Trigonal Te shows a band gap from 0.33 eV to 1.43 eV as the thickness decreases, and has a melting point of approximately 450 °C [59]. Te plays a significant role in the development of OTS materials, as evidenced by the first discovered As–Te–I OTS system, as well as the pioneering Te–As–Si–Ge OTS materials. However, despite the extensive exploration of various ternary and even binary Te-based chalcogenides exhibiting OTS behavior, the intrinsic properties of Te as a standalone element have long been overlooked. It wasn’t until 2021 that Shen et al*.* discovered elemental Te selector switch based on the novel crystal-liquid–crystal phase transition mechanism, intrinsically differing from conventional OTS selectors [60]. Pure-Te device exhibited a large drive current density > 11 MA cm^−2^, a rather high selectivity of 10^3^, a *I*_off_ < 10^−6^ A, and a fast switching speed < 20 ns [60, 61]. The ~ 0.95 eV Schottky barrier formed at Te/TiN interface enabled an *I*_off_ of 0.4 μA, which reduced to ~ 50 nA after 35 at.% Zn-doping and further to 88 pA after 50 at.% Mg-doping with a large 3 eV mobility gap. Noticeably, resembling the elemental Te cell, the Zn/Mg-doped Te layer remained in the crystalline state in the off-state [62, 63].

**Fig. 11:**
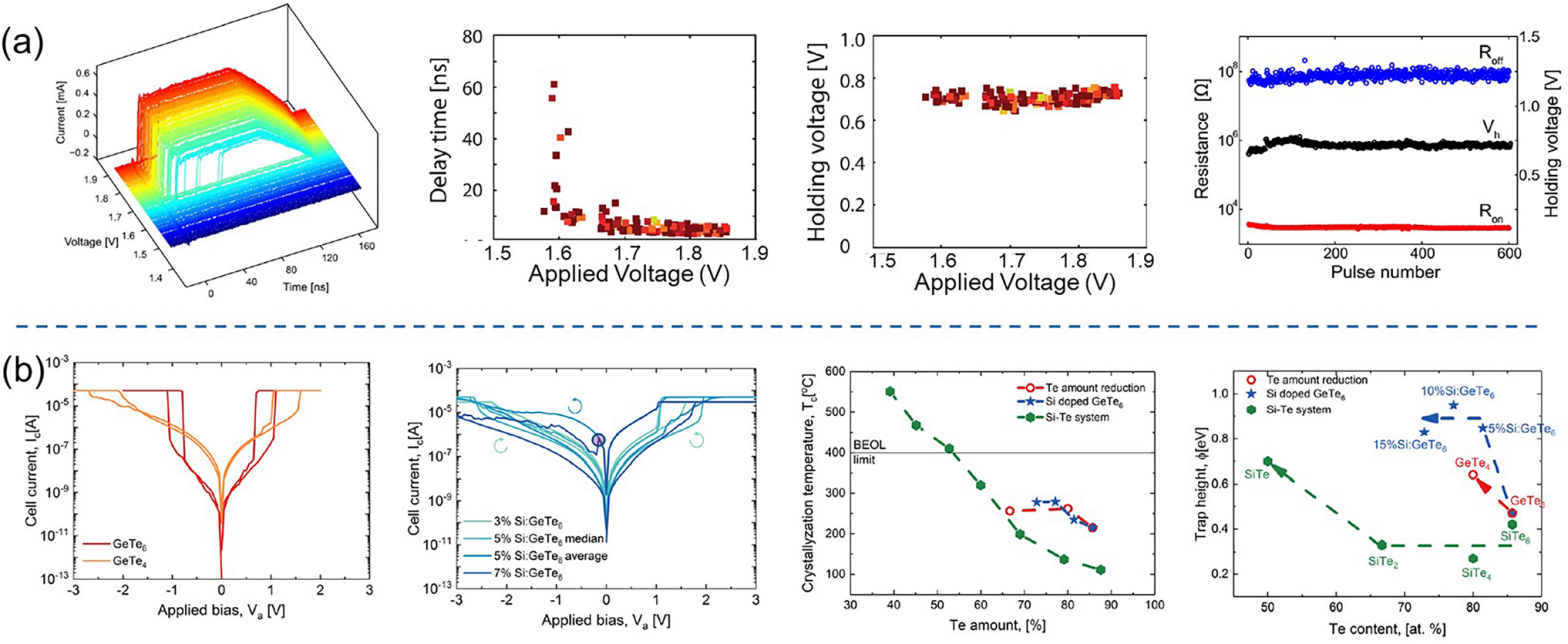
Ge–Te and Si-doped Ge–Te OTS selectors. **a** Time-resolved measurement, switching speed, *V*_hold_, and endurance of GeTe_6_ OTS cells. Adapted with permission from [64]. Copyright 2012, AIP Publishing. **b** DC *I–V* curves of GeTe_4_ and GeTe_6_ cells; DC *I–V* curves of Si-doped GeTe_6_ cells; Crystallization temperature and trap positions of Ge–Te, Si-doped GeTe_6_, and Si–Te materials [64, 65]. Adapted with permission from [65]. Copyright 2017, Springer Nature

Binary Te-based systems have attracted attention due to their simplicity in composition and environmental friendliness. Among binary Te OTS materials, Ge–Te alloys is the earliest studied and popular OTS matrix. In 2012, Anbarasu et al*.* first reported that GeTe_6_ cell surprisingly presented an OTS behavior, rather than an OMS one found in typical GeTe device, with 5 ns fast switching speed and 0.65 mA *I*_on_ and 600-time cycles (Fig. 11a) [64]. Then GeTe_4_ OTS materials were also found by Velea et al*.* (Fig. 11b) [65], the *V*_th_ of which increased from 1.2 eV to 1.55 V and the on/off ratio degraded from 5 × 10^3^ to 10^2^ (compared to GeTe_6_)*.* Since the low mobility gap width of Ge–Te materials ranged from 0.33 to 0.9 eV (Fig. 10d), Ge–Te OTS selector presented a low *V*_th_ of < 1.7 V@ 20 nm thick. Ge–Te devices exhibited good consistency among different device units, with low fluctuations observed during multiple tests of the same device unit. However, the narrower mobility gap also led to a large leakage current > 10^–8^ A. Additionally, as pure Te crystallizing at −10 °C, the highest crystallization temperature was found to only ~ 260 °C even increasing Ge content to 33.3 at.% (GeTe_2_) (Fig. 10b), far lower than the 400–450 °C required for the BEOL process. It also means that the Ge–Te films are more likely to crystallize during switching, which is one of the main reasons for the reduced endurance of the OTS selector (< 1000 cycles) [64]. From a bond energy perspective, the Te atomic radius is 1.4 Å, and the Ge–Te bond energy is 192 kJ mol^−1^ [66], indicating longer bond lengths and weaker bond energies. This makes it less capable of withstanding high temperatures and large current.

**Fig. 12:**
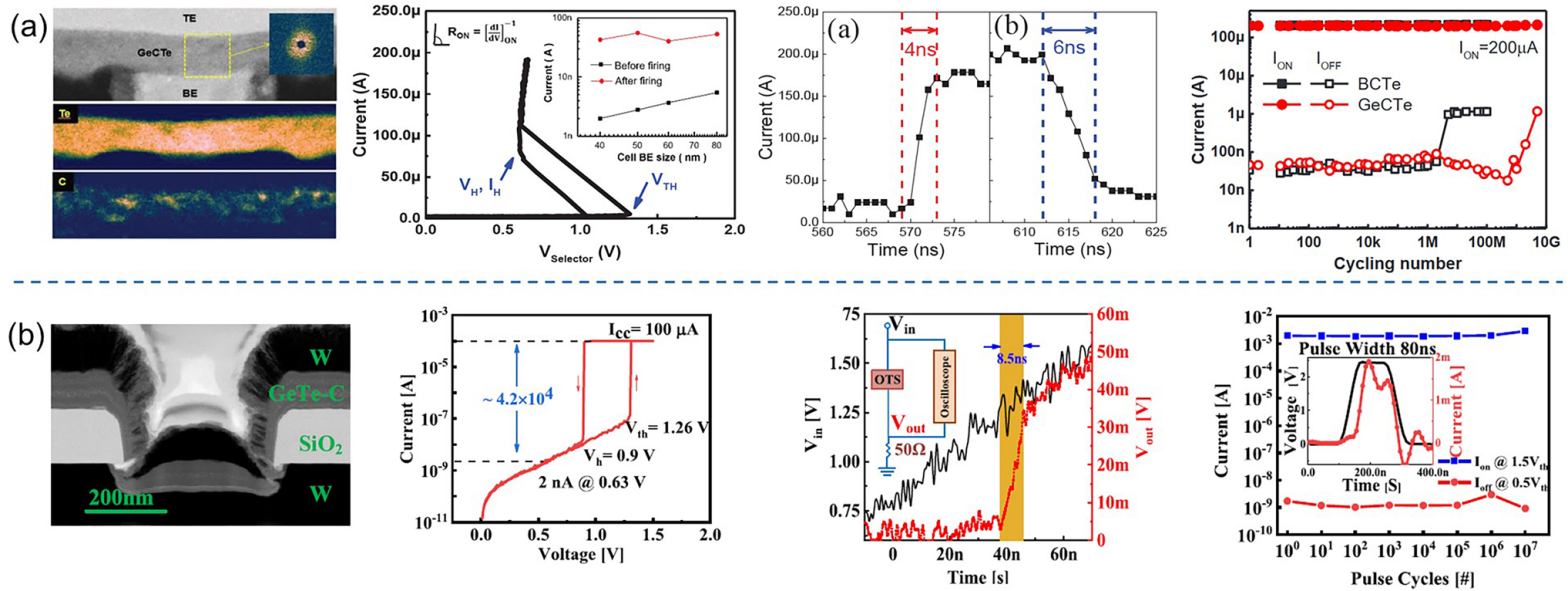
Ge–Te–C OTS selectors. **a** Device structure (30-nm plug), AC *I*–*V* curves, switching speed, and endurance of Ge–Te–C OTS cells reported by Wu et al*.* [35]. **b** Device structure (250-nm BE), DC *I*–*V* curves, on-switching speed, and endurance of Ge–Te–C OTS cells reported by Wang et al. Adapted with permission from [69], Copyright 2021, IEEE

In order to solve the problem of insufficient thermal stability of GeTe_6_ itself, Velea et al*.* proposed three optimization paths: reducing Te content, adding Si element, and directly replacing Ge with Si to form Si–Te system [65] (Fig. 11b). The reduction of Te content alone cannot significantly increase the crystallization temperature; in that report, the maximum increase was only 45 °C, and there was a clear bottleneck for further improvement. After 2 at.% Si doping, it exhibited OTS switching behaviors in current-limiting tests [67], whereas non-volatility occurred as the Si content exceeds 5 at.% [68]. Besides, to improve the thermal stability and reduce the *I*_off_, in 2021, Wu et al*.* [35] doped C into the Ge–Te alloy, which sharply extended the device lifetime to > 10^11^-cycle and exhibits an *I*_off_ < 5 nA, a switching speed about 5 ns, and a *V*_th_ of 1.3 V (Fig. 12a). In the same year, Wang et al*.* [69] also investigated 5 at.% C-doped Ge–Te OTS selector, presenting *I*_on_ = 2 mA, *I*_off_ = 2 nA, 8.5 ns on-speed, and > 10^7^ cycles endurance (Fig. 12b).

**Fig. 13:**
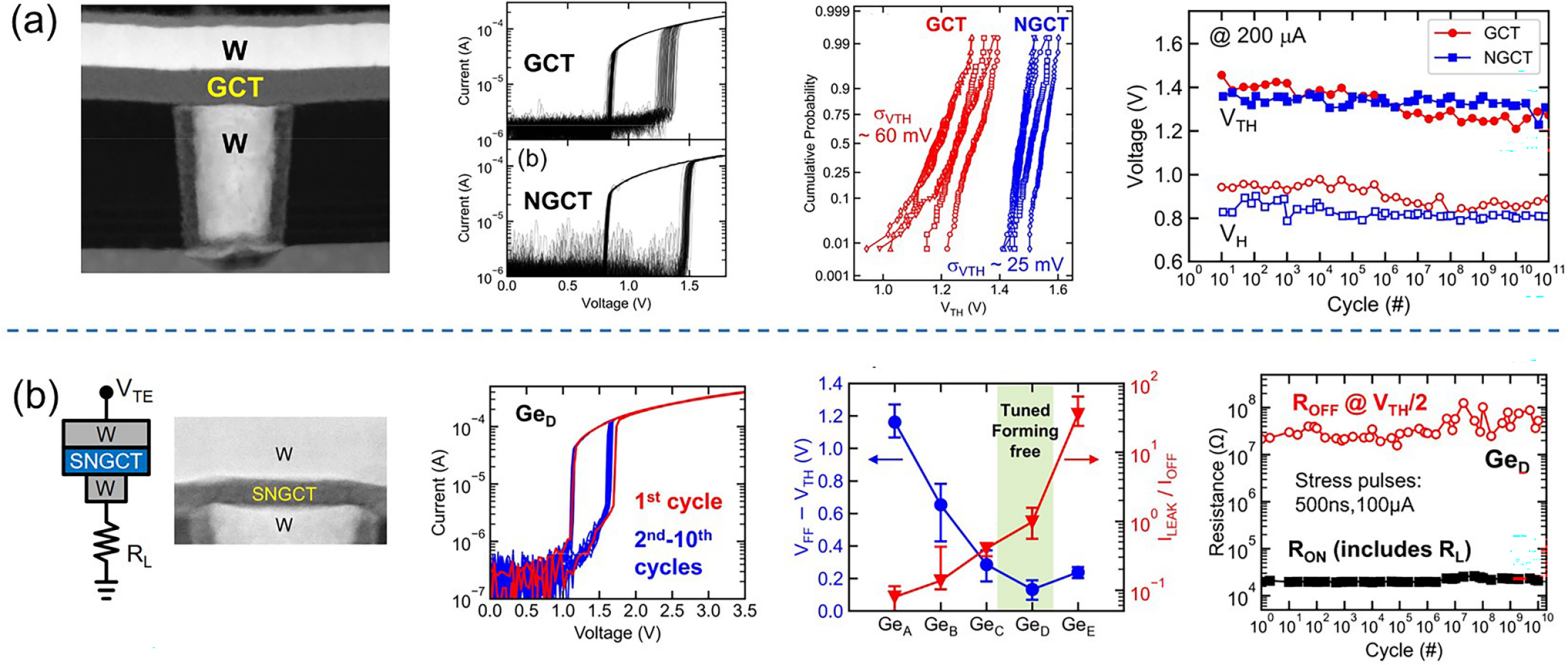
Ge–Te–C–N (**a**) and Ge–Te–C–N–Si (**b**) OTS selectors. **a** Device structure of a Ge–Te–C–N OTS cell (32–72-nm plug); 100 DC switching cycles; Cumulative probability plot of *V*_th_; Endurance. Adapted with permission from [70]. Copyright 2021, IEEE. **b** Device structure of a Ge–Te–C–N–Si cell (~ 40-nm plug); DC *I–V* curves after repeated operation; Firing-free optimization plot showing firing-free behavior in Ge_D_ composition; Endurance. Adapted with permission from [71]. Copyright 2022, IEEE

Ambrosi et al*.* from TSMC further incorporated N into the Ge–Te–C OTS materials to improve the thermal stability above 400 °C [70], enabling even smaller performance fluctuations, reducing parameter dispersion such as *V*_th_ after multiple operation, and exceeding 10^11^ cycles (Fig. 13a). The further introduction of Si elements into the system enhanced the robustness of the device. The thermal stability and high-current tolerance of the Ge–Te–C–N–Si system were further enhanced. The device’s thermal stability could be improved to above 450 °C, and the voltage drift was reduced compared to the Ge–Te–C and Ge–Te–C–N systems, resulting in significantly improved device reliability with endurance > 10^10^ cycles (Fig. 13b). Additionally, by controlling the Ge content, modulation of defect states in the Ge–Te–C–N–Si system could effectively reduce the difference between *V*_ff_ and *V*_th_ within a specific compositional range, achieving a "firing-free" effect [71] (Fig. 13b). This optimization further reduced the requirement for *I*/*O* voltage in application scenarios and facilitated the application of OTS devices in more advanced process nodes.

**Fig. 14:**
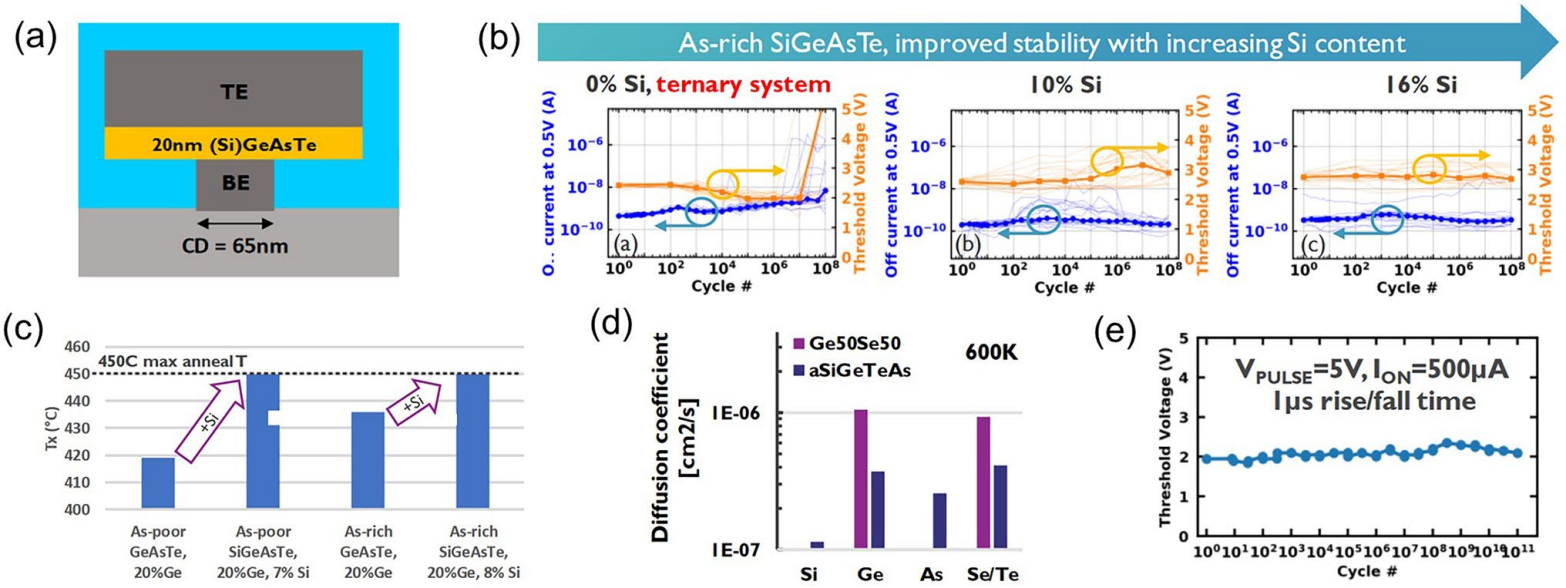
Ge–Te–As OTS selectors. **a** Device structure (65-nm plug). **b** Endurance of As-rich Ge–As–Te–Si OTS devices with different Si content. **c** Crystallization temperature, moving from Ge–Te–As to Ge–Te–As–Si system increases > 450 °C. **d** Comparison of calculated element diffusion coefficient at 600 K for GeSe and Si–Ge–As–Te. **e** Endurance of 10^11^ cycles achieved Ge–Te–As. Adapted with permission from [72]. Copyright 2019, IEEE

Besides C/N/Si doping in Ge–Te OTS cells, the toxic As elements were often incorporated. In 2019, Garbin et al*.* provided a comprehensive analysis of the effects of the As/Te ratio, Ge and Si content in the Ge–Te–As–Si system on device performance (Fig. 14). Increasing the As/Te ratio raised the crystallization temperature [72]. The addition of Ge to AsTe reduced the mobility gap, and when the Ge content in Ge–Te–As exceeded 20 at.%, the crystallization temperature increased to 450 °C. However, the mobility gap decreased and leakage current increased. The addition of Si increased the mobility gap, raised the crystallization temperature, increased the threshold voltage (*V*_th_) to a range of 2.3–2.9 V, and reduced leakage current to 10 nA. In the Ge–Te–As–Si system, strong bonds were formed, resulting in a temperature-stable amorphous network with excellent endurance (10^11^ cycles).

**Fig. 15:**
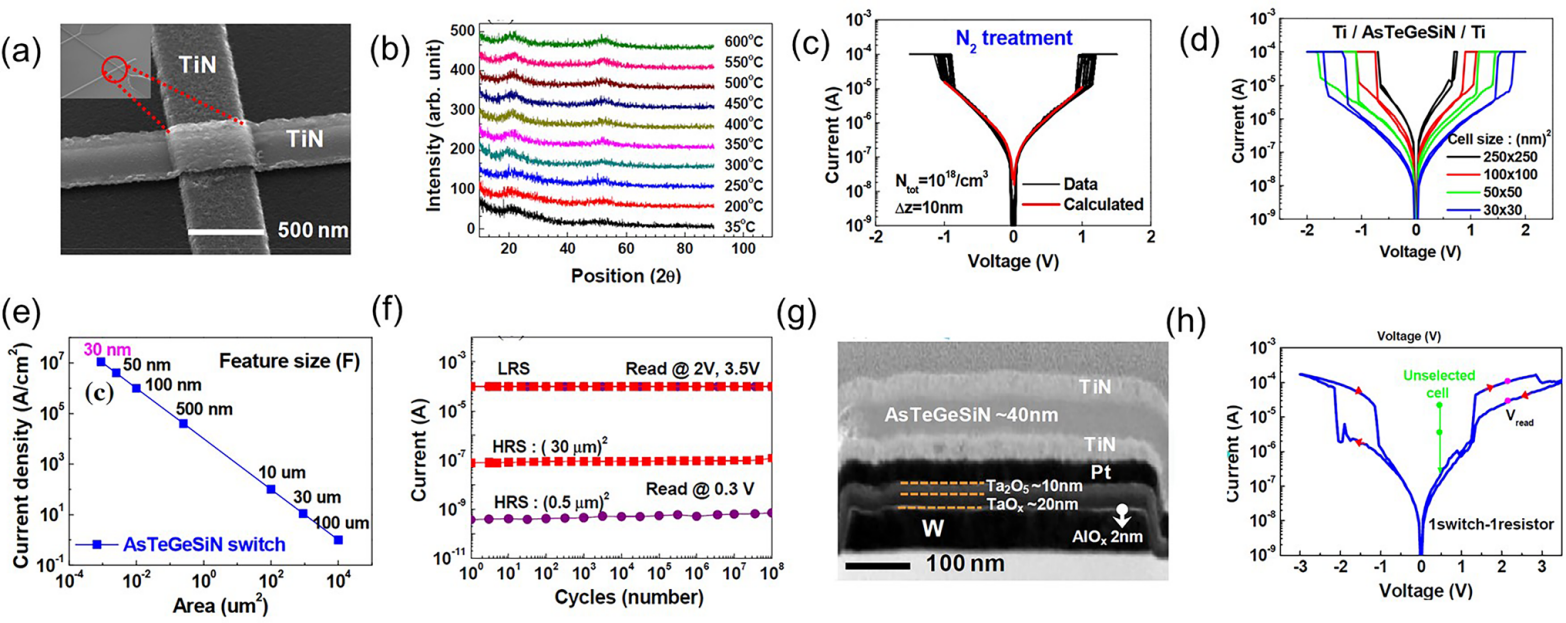
Ge–Te–As–Si–N OTS selector and 1S1R cell. **a** Device structure with 30 nm–100 um size. **b** Thermal stability above 600 °C. **b** DC *I*–*V* curves. **c** DC *I*–*V* curves of the selector with varying sizes. **e** Scaling behavior. **f** Endurance. **g** 1S1R structure with Ta_2_O_5_/TaO_*x*_ memory cell. **h** DC *I*–*V* curves of the 1S1R cell. Adapted with permission from [73]. Copyright 2012, IEEE

Lee et al*.* further enhanced the thermal stability to above 500 °C by introducing nitrogen (N) into the Ge–Te–As–Si and solved the performance degradation with repeated cycling and reliability after BEOL process (Fig. 15) [73]. A *I*_on_ of 0.1 mA was obtained, which were found to be size-dependent. For a 30 nm cell, a *J*_on_ > 11 MA cm^−2^ was found. Their cycling performance was shown to be greater than 10^8^ cycles. Also, they demonstrated a 1S1R memory cell using a Ta_2_O_5_/TaO_x_ resistance memory with the GeTeAsSiN selector device.

Beside the Ge–Te-based OTS materials mentioned above, there are some binary Te-based OTS materials worth mentioning, including Si-doped, C-doped, B-doped, and Al-doped Te; most of them were first reported by Kazuhiro Ohba and Hiroaki Sei from Sony corporation in 2015 [74]. In their filed OTS patents, they also investigated N, O, Mg, Ga, Y etc. incorporation, for thermal stability enhancement and leakage current reduction, and multi-element doping. They further noticed that the insertion of 2–5 nm high-resistance layer between the OTS layer and electrode, like SiO_*x*_, AlO_*x*_, MgO_*x*_, SiN, and HfO_*x*_, could greatly reduce the *I*_off_ to sub-nA and even pA. For Si–Te OTS materials, Velea et al*.* mentioned the crystallization temperature of Si–Te system increased significantly with the decrease of Te content, it could reach 400 °C when Te < 50 at.% [65]. Similar results were found in Koo et al*.*’s work [75]. The difference was that the electrical performance of Si–Te reported in the former paper was seriously degraded, the selectivity of other Si–Te components was less than 5 except for SiTe_6_ (~ 100), while Koo et al*.* reported that Si–Te device had good performance, including an *I*_on_ of 5 × 10^−4^A, *I*_off_ of ~ 1 nA, thermal stability up to 400 °C, and endurance up to 10^8^ cycles.

**Fig. 16:**
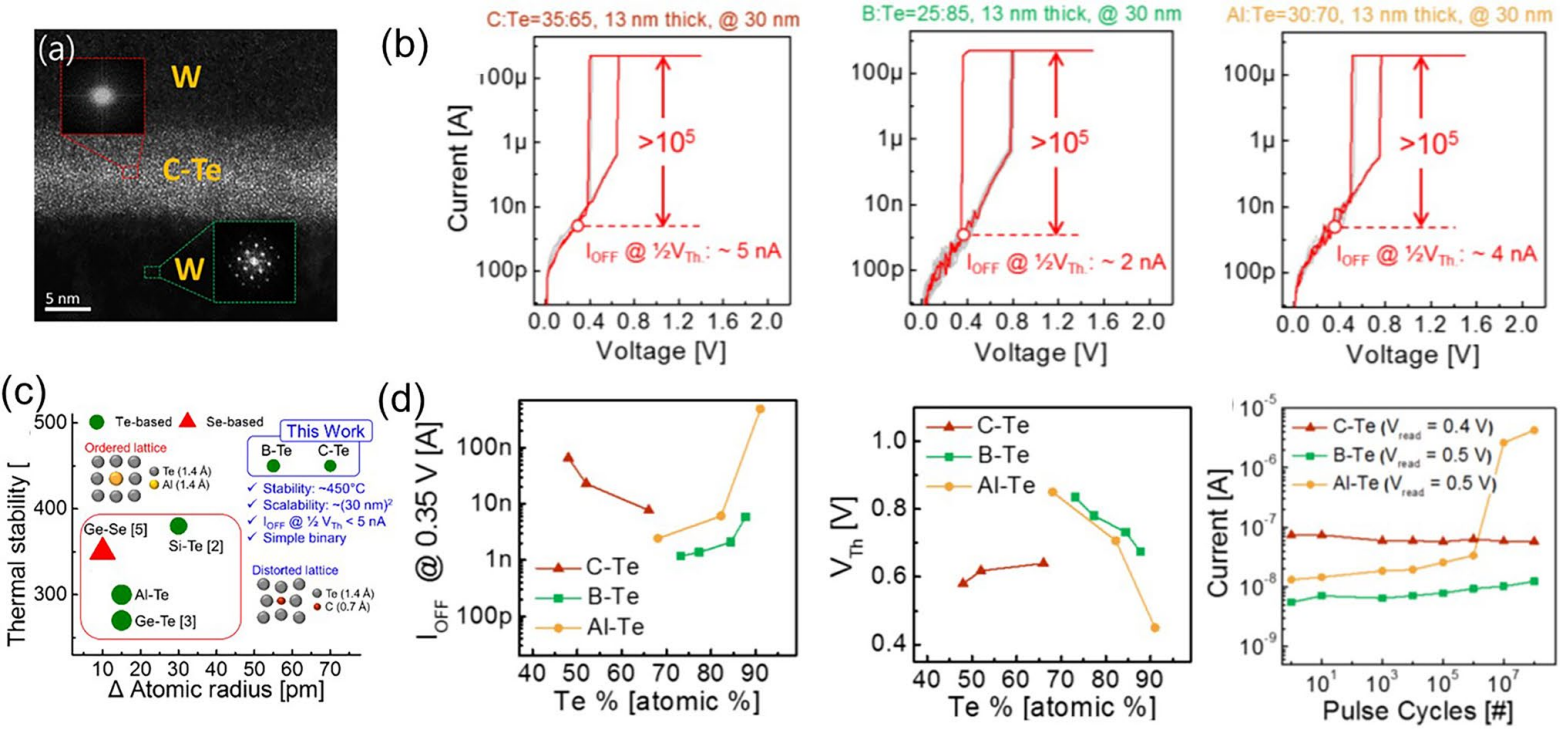
C–Te, B–Te, and Al–Te OTS selectors. **a** Device structure with 30–150 nm size. **b** DC *I*–*V* curves. **c** Relation between thermal stability and atomic radius difference. **d** *I*_off_, *V*_th_ and endurance. Adapted with permission from [76]. Copyright 2018, IEEE

The C–Te system is another Te-based OTS material that has been extensively reported recently. In 2018, Chekol et al*.* chose carbon (C) as a component in the C–Te OTS device [77], which had a smaller atomic radius compared to Ge and Si. The C–Te OTS device exhibited a switching ratio greater than 10^5^, a current density higher than 11 MA cm^−2^, an *I*_off_ current of approximately 1 nA, high endurance of around 10^9^ cycles, and thermal stability exceeding 450 °C (Fig. 16b). The *V*_th_ of C–Te was relatively small (*V*_th_ = 0.64 V, *V*_hold_ = 0.36 V). C–Te was stable when the C content was between 25 and 55 at.%. As the C content increased, *V*_th_ decreased, while leakage current increased. In 2018, Yoo et al*.* reported the B–Te system [78], which exhibited a selectivity of 10^5^, *I*_on_ of 5 × 10^−4^A, *I*_off_ less than 10 nA, thermal stability up to 450 °C, and endurance of 10^8^ cycles (Fig. 16b). In the same year, Yoo et al*.*’s comprehensive research showed that in B–Te and Al–Te-based devices [76], the decrease of Te ratio resulted in increased *V*_th_ and decreased *I*_off_ (Fig. 16d). The endurance of devices based on C–Te and B–Te could switch > 10^8^ cycles in maintaining low *I*_off_, while the Al–Te-based device showed *I*_off_ degradation after 10^7^ cycles (Fig. 16d**)**.

Table 3 summarizes the device performances of Te-based OTS selectors. Te-based OTS selectors present a *J*_on_ ranging from 0.44 MA cm^−2^ to 55 MA cm^−2^, an *I*_off_ ranging from 10^–7^ A to 10^–9^ A, a *V*_th_ ranging from 0.75 V to 2.2 V, and a *V*_hold_ ranging from 0.3 V to 1.5 V, and an endurance ranging from 600 to 10^11^ cycles. Although Ge–Te OTS materials show lower than 250 °C thermal stability, the incorporation of light atoms (with a smaller radius) or the replacing of Ge by these atoms, forming strong bond and robust amorphous network, significantly improve the thermal stability above 400 °C, enabling the withstanding of the BEOL process in the memory fabrication.

**Table 3:** Performance summary and comparison of Te-based OTS devices

Materials		Feature size/nm	Selectivity	*J*_on_/MA cm^−2^	*I*_off_/A	*V*_th_/V	*V*_hold_/V	Speed/ns	Endurance	Thermal stability/°C
B–Te [76]		30	10^5^	~ 55	~ 10^–8^	~ 0.75	~ 0.3	~ 2	10^8^	> 450
Si–Te [75]		100	10^6^	10	~ 1 × 10^–9^	~ 1.2	~ 1	~ 2	10^8^	> 400
C–Te [77]		30	10^5^	11	5 × 10^–9^	~ 0.64	~ 0.36	~ 2	> 10^8^	> 450
GeTe_6_ [64]		60	10^5^	~ 1.8	–	~ 1.6	~ 0.7	–	600	–
Ge–Te–C [35]		30	10^5^	~ 24.8	3 × 10^–9^	1.32	0.62	–	> 10^11^	< 300
Ge–Te–C–N [70]		~ 40	> 10^4^	~ 8	3 × 10^–9^	~ 1.4	~ 1	–	> 4 × 10^11^	400
Ge–Te–Si–N–C [34]		~ 32	10^4^	12.4	~ 10^–8^	1.7	~ 1	–	> 10^10^	> 450
Ge–Te–As [79]		110	10^5^	17	3 × 10^–8^	~ 0.75	~ 0.6	~ 10	–	–
Ge–Te–As–Si–N [80]		30	10^3^	11	~ 10^–7^	~ 1.8	~ 1.5	~ 4	10^8^	> 450
Ge–Te–As–Se–Si [51]		350	10^4^	0.44	1.9 × 10^–9^	~ 2.2	~ 1.5	~ 50	10^10^	> 350

### S-based OTS

**Fig. 17:**
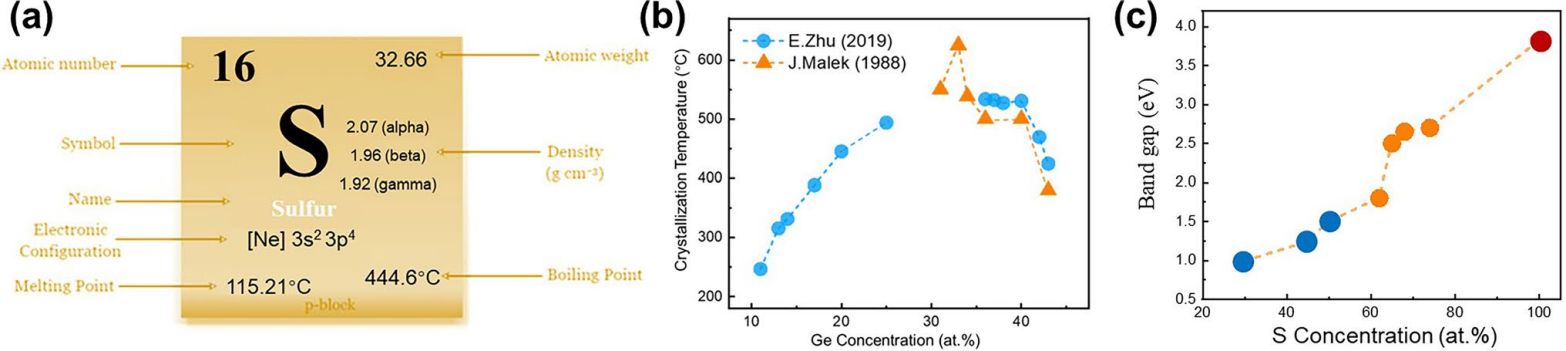
Elemental S and Ge–S properties. **a** Element sulfur’s properties. **b** Crystallization temperatures and **c** the mobility gap of Ge–S alloys [81–83]

As the most representative element in the chalcogen, the 16th element sulfur (S) has been the subject of numerous investigations, regarding its crystal structure [84–89], electronic structure [84, 85, 90–92], thermodynamic properties [84], and other physical characteristics (Fig. 17a). The crystallization temperature of pure S is about 0 °C and the melting point is 115 °C [93]. In 2020, Jia et al*.* first reported S-based OTS materials [66], GeS, and then Ge–S alloys were systematically studied. Since the atomic radius of S atoms was just 0.88 Å, > 0.3 Å smaller than Se and Te atoms, the Ge–S compounds exhibited high crystallization temperatures, 380 °C for GeS and exceeding 600 °C for GeS_2_ [92]. The mobility gap of Ge–S system increased with an elevated concentration of S, for S-rich Ge–S, the mobility gap ranged from 2.6 V to 3.4 eV and it could reach 3.8 eV in pure S (Fig. 17b, c) [94]. Consequently, Ge–S-based OTS device exhibited a rather low *I*_off_. The Ge–S bond energy is 266 kJ mol^−1^ [66], indicating a strong bond that enables the material to withstand higher temperatures and larger passing current.

**Fig. 18:**
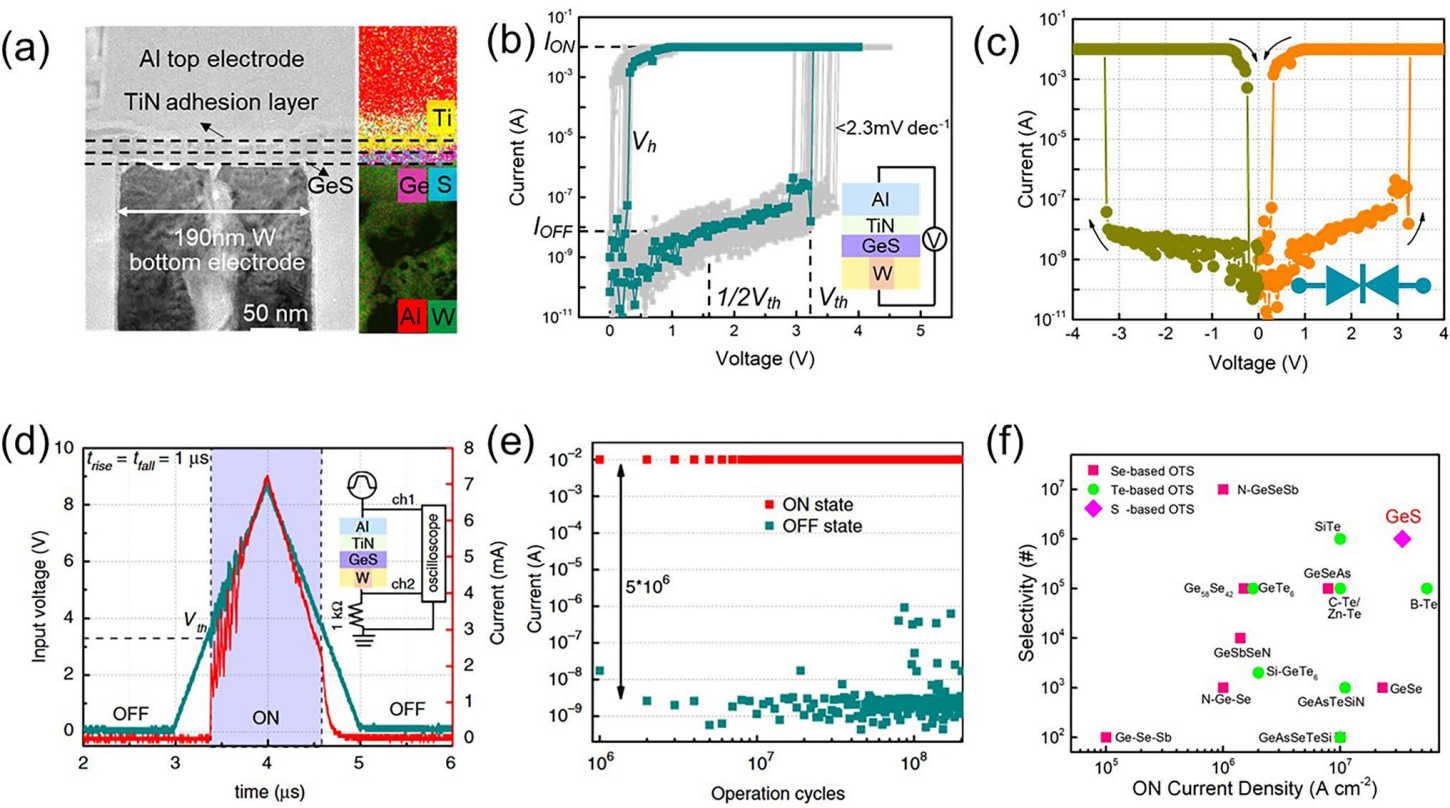
GeS OTS selectors. **a** Device structure with 190-nm plug. **b** DC *I*–*V* curves. **c** DC bi-directional curves. **d** AC speed test. **e** Endurance > 10^8^. **f** Comparison of selectivity and *J*_on_ of reported OTS devices [66]. Adapted with permission from [66]. Copyright 2020, Springer Nature

In 2020, Jia et al*.* discovered novel GeS OTS devices from the perspective of constructing a more stable amorphous system [66]. The device consisted of W bottom electrode with a diameter of 190 nm, Al top electrode, and the 10-nm-thick GeS layer sandwiched between them (Fig. 18a). Due to strong Ge–S bond and wide mobility gap of GeS (~ 1.5 eV), the GeS selector exhibited high drive current ~ 10 mA (*J*_on_ = 34 MA cm^−2^) and low leakage current ~ 10 nA (Fig. 18b). The *V*_th_ in the selector was ~ 3.2 V, while the *V*_hold_ was ~ 0.3 V (Fig. 18b). The GeS OTS device demonstrated favorable bi-directional selectivity and scalability (Fig. 18c), with a high switching speed (*t*_on_ ~ 10 ns, *t*_off_ ~ 100 ns) (Fig. 18d), an on/off ratio of approximately 10^6^ and endurance exceeding 10^8^ (Fig. 18e). In terms of thermal stability, the device maintained switching performance after annealing at 350 °C. Considering the trade-off between selectivity ratio and high driving current, GeS devices demonstrated remarkable performances, when compared to Se and Te selectors (Fig. 18f).

**Fig. 19:**
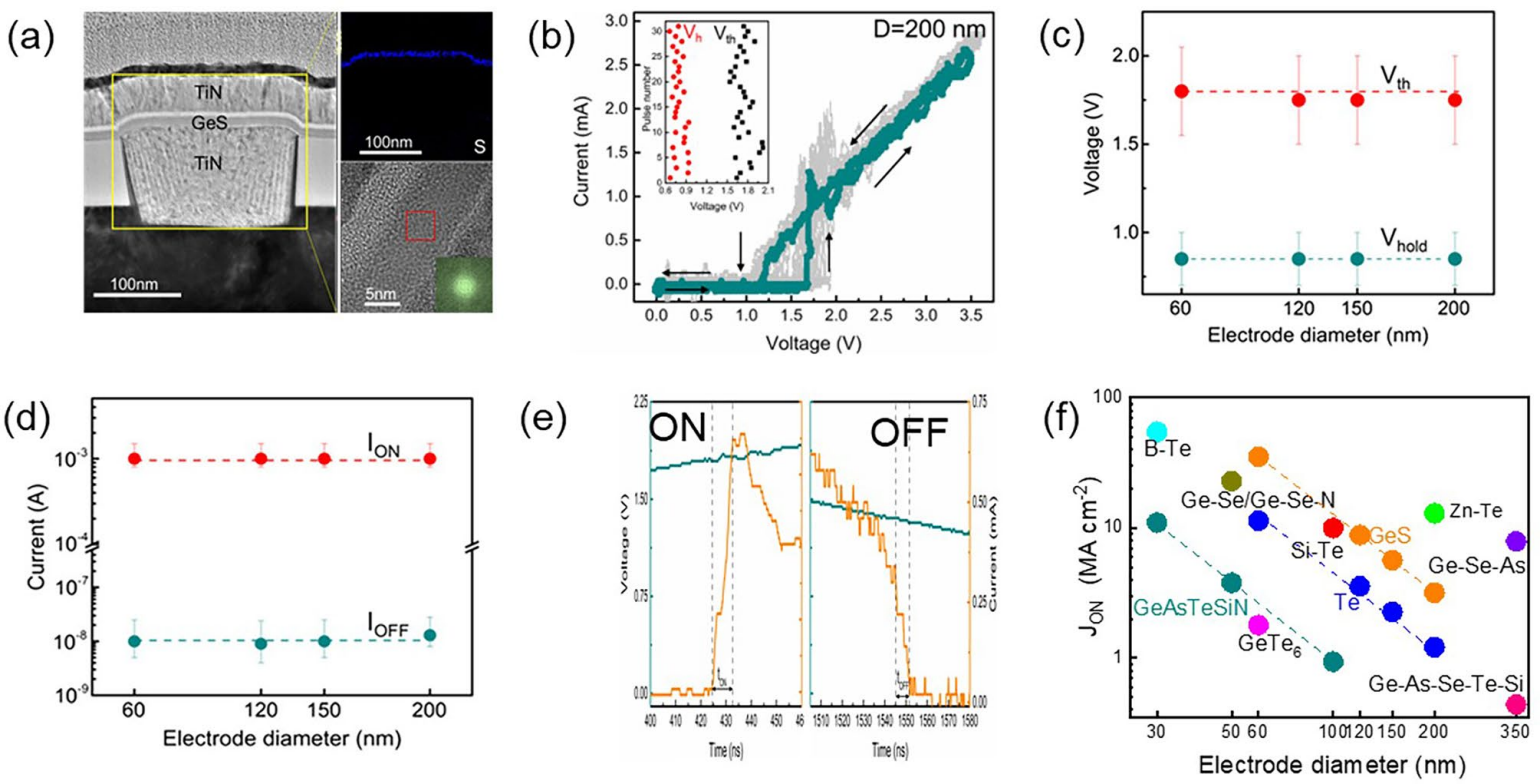
GeS OTS device with TiN electrodes. **a** Device structure with 60–200-nm plug. **b** AC *I*–*V* sweeps. **c**, **d** *V*_th_, *V*_hold_, *I*_on_ and *I*_off_ dependence on the electrode size. **e** On/Off speed; **f** Comparison of the *J*_on_ of the GeS OTS cell with other Te- and Se-based selectors [95]. Adapted with permission from [95]. Copyright 2021, John Wiley and Sons

It is worth noting that the top electrode in that paper was made of Al, which would cause the diffusion of Al atoms and form conductive channels in the material layer, thus affecting the switching process. In the subsequent report [95], the author reported GeS OTS devices with both TiN top/bottom electrodes (Fig. 19a). The GeS device with TiN electrodes (Fig. 19a) showed a lower *V*_th_ of 1.75 V and a higher* V*_hold_ of 0.75 V (Fig. 19b, c), the selector exhibited an *I*_on_ of 1 mA, and large current density ~ 35.4 MA cm^−2^ in 60 nm-sized devices (Fig. 19d). Noticeably, the off-speed in this selector is more than 10 times faster (~ 7 ns) than the previous one (Fig. 19e), indicating that the diffusion of Al slowed down the switching process (also increased the *I*_on_), but this effect did not lead to significant changes in the main switching mechanism of the device, which was still an OTS switching. Compared with other selectors, GeS OTS selector still had obvious advantages in the drive current density and could be further improved with the feature size scaling down (Fig. 19f).

**Fig. 20:**
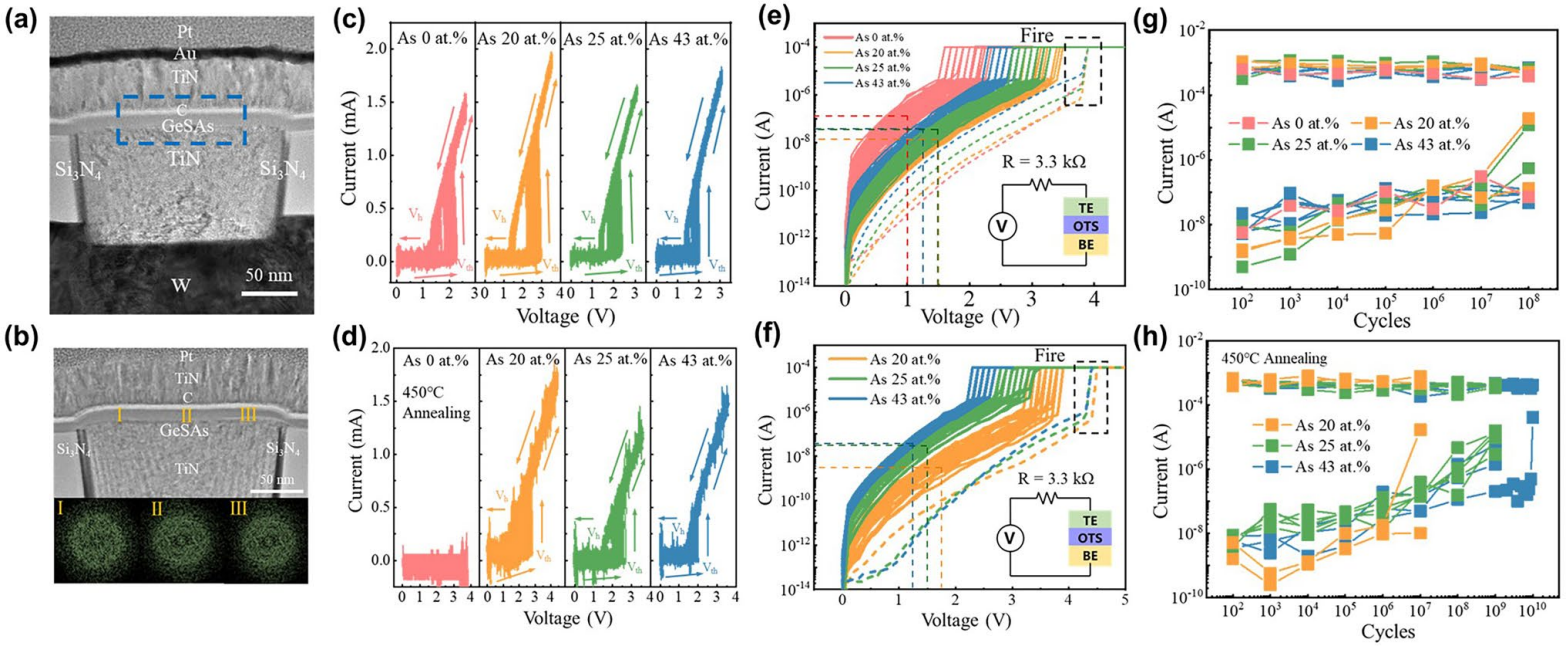
GeSAs OTS selectors. **a**, **b** Device before/after 450 °C annealing. **c**, **d** *I*–*V* curves of Ge–S–As devices before/after 450 °C annealing. **e**, **f** DC *I*–*V* curves of devices before/after 450 °C annealing. **g**, **h** Endurance performance of Ge–S–As before/after 450 °C annealing. Adapted with permission from [96]. Copyright 2023, Springer Nature

In 2023, Wu et al*.* reported the Ge–S–As OTS device with various As concentrations and systematically elaborated the key role of As element in OTS switching [96]. They discovered that incorporation of As into GeS brought a more than 100 °C increase in crystallization temperature (> 450 °C) (Fig. 20a, b), improving the switching repeatability and prolonging the device endurance (~ 10^10^ cycles), which was attributed to the strengthened As-S bonds and sluggish atomic migration after As incorporation. The addition of As reduced the leakage current by more than an order of magnitude (Fig. 20e, f) and significantly suppressed the operational voltage drift, ultimately enabling a BEOL-compatible OTS selector with a *V*_th_ of 2 V, an *I*_on_ higher than 12 MA cm^−2^, an on/off ratio over 10^4^, a speed about 10 ns, and an endurance approaching 10^10^ cycles after 450 ℃ annealing (Fig. 20h).

**Fig. 21:**
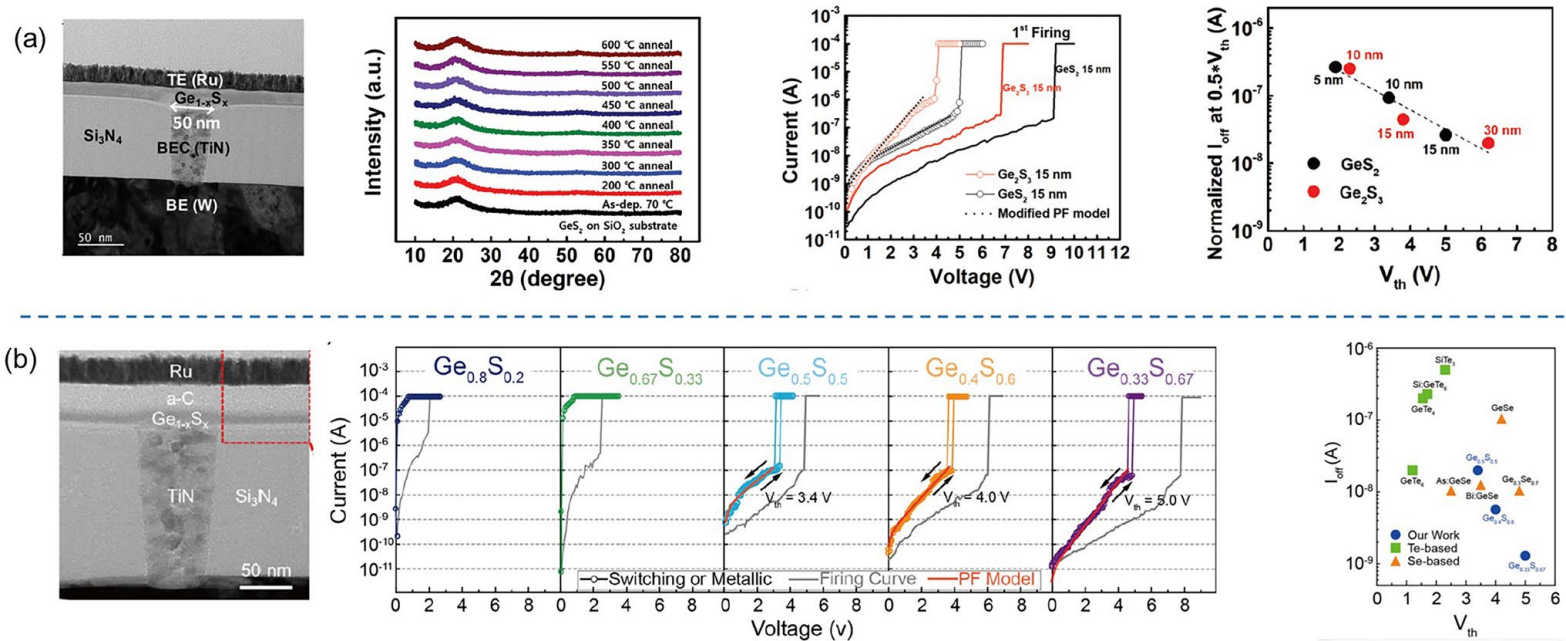
Ge–S OTS selector. **a** Device structure with 50-nm plug; Thermal stability of GeS_2_ above 600 °C; DC *I*–*V* curves of GeS_2_ and Ge_2_S_3_ cells; *I*_off_. Adapted with permission from [94]. Copyright 2021, Royal Society of Chemistry. **b** Device structure with 60-nm plug; DC *I* *V* curves of Ge–S devices; *I*_off_ and *V*_th_ comparisons [53, 94, 97]. Adapted with permission from [97]. Copyright 2023, Elsevier

In 2021, Kim et al*.* fabricated GeS_2_ and Ge_2_S_3_-based OTS devices using the plasma-enhanced atomic layer deposition (PE-ALD) [94]. Through comprehensive considerations of factors such as growth temperature and purging time of the GeCl_4_ precursor, the team discovered that PE-ALD Ge_1-*x*_S_*x*_, utilizing an H_2_S/Ar plasma reactant, exhibited a self-limiting growth behavior within an ALD window. As a result, the team successfully fabricated GeS_2_ and Ge_2_S_3_ films by ALD, which exhibited thermal stability up to 600 °C (Fig. 21a). The 15-nm-thick GeS_2_ device exhibited an *I*_on_ of 0.1 mA, a *V*_th_ of approximately 5 V and a *I*_off_ of 20 nA. The trade-off relationship between the *V*_th_ (1.9–6.2 V) and the normalized* I*_off_ (20–250 nA) was observed by scaling down the film thickness from 30 to 5 nm (Fig. 21a). Surprisingly, Ge_2_S_3_ OTS devices required lower *V*_th_ owing to lower trap density according to the Poole–Frenkel fitting (Fig. 21a).

In 2023, Lee et al*.* from the same group continued the exploration of Ge_1−*x*_S_*x*_ series OTS devices [97], which revealed that Ge-rich compositions (0.2 ≤ *x* < 0.5) exhibited metallic behavior due to Ge crystallization caused by Joule heating. On the other hand, devices with S-rich compositions (0.5 ≤ *x* ≤ 0.67) demonstrated OTS behaviors (Fig. 21b). As the S content increased, the material’s mobility gap widened from 1.65 eV for Ge_2_S to 3.45 eV for GeS_2_. Consequently, the devices exhibited increased *V*_th_ (ranging from 3.4 V to 5 V), reduced *I*_off_, with the lowest reaching 1.3 nA (Fig. 21b). The endurance of the devices reached up to 10^9^ cycles, and the devices exhibited thermal stability up to 600 °C. Note that mobility gap values of Ge–S alloys in this work was > 1 eV larger than reported film samples which may be due to the material contamination [97].

**Fig. 22:**
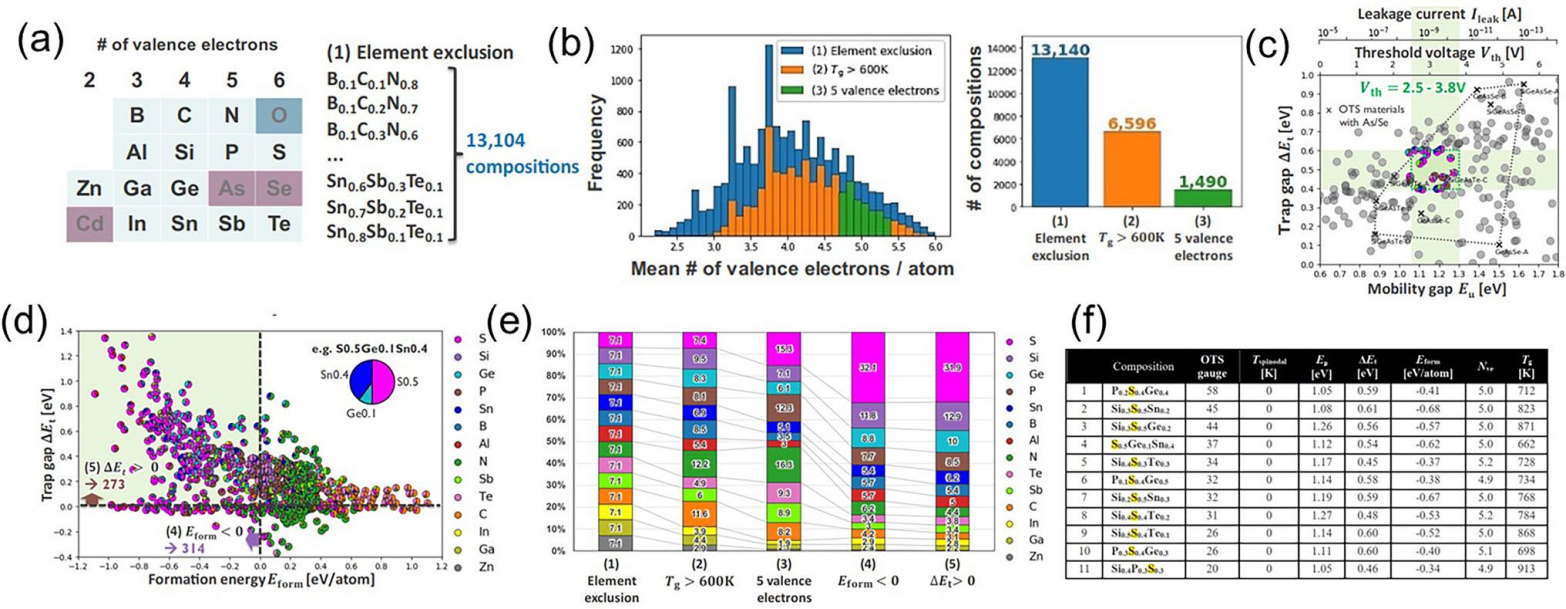
Screening environment-friendly OTS materials. **a** The first screening filter of element exclusion for new OTS materials. **b** The screened compositions for the valence electrons number per atom *N*_ve_, the glass-transition temperature (*T*_*g*_) and the 5 valence-electron rule. **c** Mobility gap *E*_μ_ and trap gap Δ*E*_t_. **d** Formation energy *E*_form_ and trap gap Δ*E*_*t*_. **e** Element breakdown of screened compositions. **f** Summary of 11 promising OTS compositions of selector materials [98]. Adapted with permission from [98]. Copyright 2022, IEEE

In 2022, Matsubayashi et al*.* conducted high-throughput calculations to screen environment-friendly ternary OTS materials [98]. The first screening filter was element exclusion to narrow down the combinations. They focused on the 14 elements: B, C, N, Al, Si, P, S, Zn, Ga, Ge, In, Sn, Sb, and Te, the ternary combination of which generated about thirteen thousand compositions (10% atomic fraction step, Fig. 22a). The second screening filter was the glass-transition temperature > 600 K, required for BEOL process, by which about six thousand compositions left. The third screening filter was the 5 valence-electron rule to populate the antibonding state (unstable) and activate the OTS behavior (Fig. 22b). For the about 1500 compositions that passed the above screening, they computed the formation energies and the electronic properties from first-principles. After considering formation energy < 0 eV atom^−1^ (chemical stability), low leakage current, immunity to phase demixing, application-specific trap/mobility gaps and change in polarizability, they finally identified 11 promising OTS materials (Fig. 22f), including P_0.2_S_0.4_Ge_0.4_, Si_0.3_S_0.5_Sn_0.2_, Si_0.3_S_0.5_Ge_0.2_ etc. Clearly, all these candidates were S-based compounds, indicating the application potential of S-based OTS materials [98, 99].

**Fig. 23:**
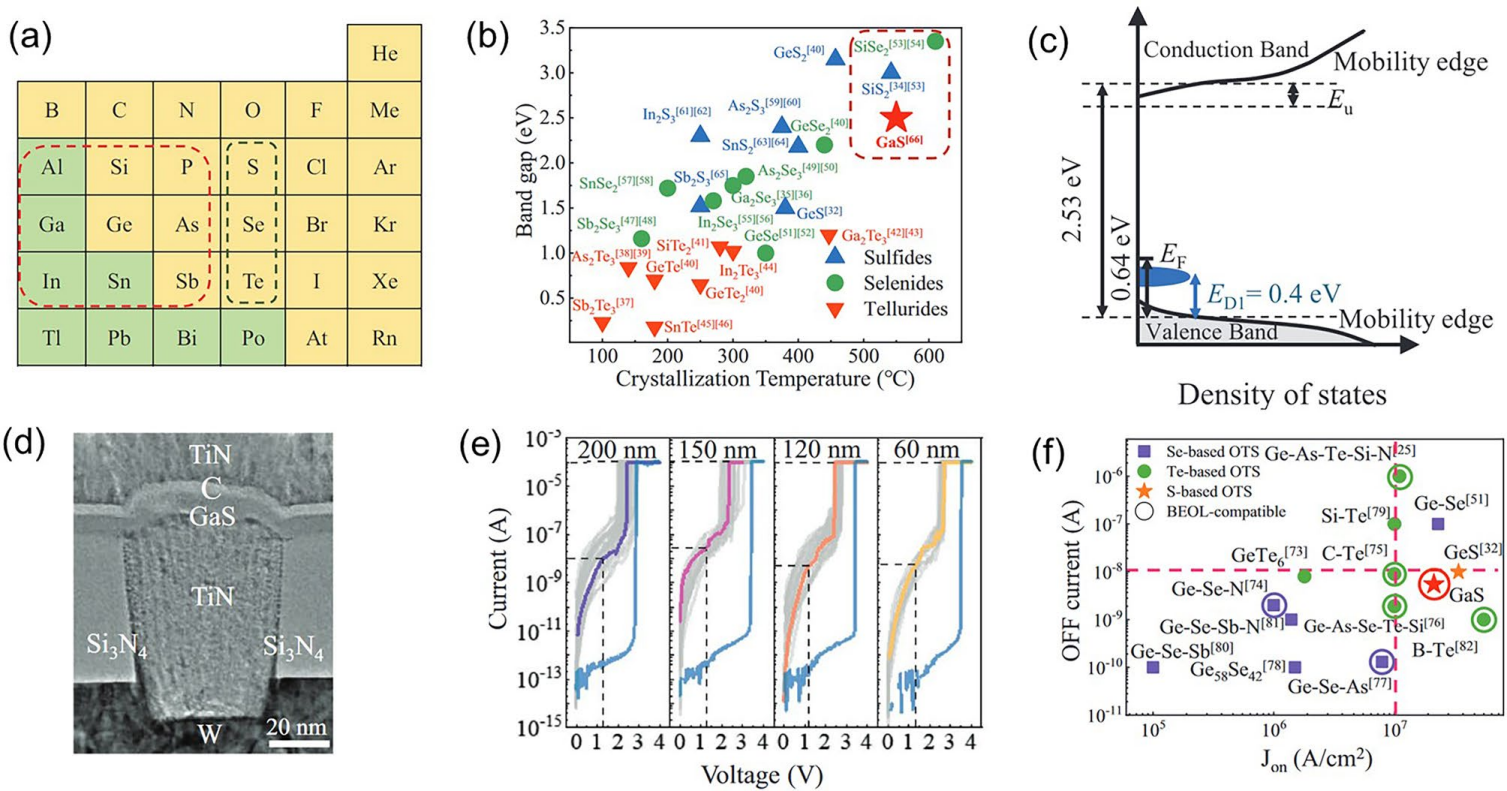
GaS OTS selectors. **a** Pick out the cation and anion from the periodic table. **b** Band gap and crystallization temperature of these binary chalcogenides. **c** Experimentally reconstructed qualitative energy band schematic. **d** Device structure with 60-nm plug. **e** DC *I*–*V* curves. **f** Comparison of the *I*_off_ and *J*_on_ with other reported selectors [100]. Adapted with permission from [100]. Copyright 2022, John Wiley and Sons

Different from the above computational screening, Wu et al*.* performed a systematic materials screening of chalcogenides from the periodical table of elements using three essential criteria—few elements (easy fabrication), wide mobility gap (low *I*_off_), and thermal stability > 400 °C (withstand BEOL process) [100]. The anions were S, Se, or Te, while cations of these chalcogenides were from group III to V located at metal–semiconductor boundary (Fig. 23a), which were widely used in phase change materials. After the above three essential criteria filtering, Ga–S was selected as the final research target due to its high mobility gap of ~ 2.53 eV and high crystallization temperature of ~ 550 °C (Fig. 23b). Photothermal deflection spectroscopy experiment detected high-density traps in GaS materials required for OTS behavior (Fig. 23c). Further device results confirmed that Ga–S-based devices exhibited typical OTS switching behaviors, with a high drive current density *J*_on_ of ~ 21.23 MA cm^−2^, a low *I*_off_ of ~ 10 nA, and a *V*_th_ of ~ 2.5 V (Fig. 23d-f).

Table 4 summarizes the device performances of S-based OTS selectors. As expected, S-based OTS selectors present large a *J*_on_ ranging from 5 MA cm^−2^ to 34 MA cm^−2^, an *I*_off_ ranging from 10^–8^ A to 10^–9^ A, a *V*_th_ ranging from 2 V to 5 V, and an endurance > 10^8^ cycles. A high thermal stability of > 350 °C and even 600 °C was also obtained. Clearly, Se-, Te-, and S-based OTS materials have their own advantages and limitations. The actual optimization process involves integrating these advantages and overcoming their shortcomings. This is why many OTS materials are combinations of these three sub-systems. Indeed, multi-component OTS materials have many performance advantages, but the increasing complexity of their compositions also brings numerous challenges. The complex multi-component systems make it difficult to achieve atomic-level compositional uniformity in OTS material thin films, and precise control of element ratios is also challenging. Consequently, complex multi-component systems are not easily prepared using advanced conformal processes such as ALD, which hinders the application of these materials in high-density 3D vertical architecture.

**Table 4:** Performance summary and comparison of S-based OTS devices

Materials	Feature size/nm	Selectivity	*J*_on_/MA cm^−2^	*I*_off_/A	*V*_th_/V	*V*_hold_/V	Speed/ns	Endurance	Thermal stability/°C
GeS [66]	190	10^6^	34	10^–8^	~ 3.2	~ 1.5	10	10^8^	> 350
GeS [95]	60	10^5^	35.4	10^–8^	~ 2	~ 1.3	7	–	–
GeS [97]	60	10^4^	3.54	2 × 10^–8^	3.4	–	10–20	10^9^	> 600
Ge_2_S_3_ [97]	60	10^5^	3.54	~ 1 × 10^–8^	4	–	12–20	10^9^	> 600
GeS_2_ [97]	60	10^6^	3.54	1 × 10^–9^	5	–	15–20	10^9^	> 600
Ge_2_S_3_(PE-ALD) [94]	50	10^4^	~ 5	4.5 × 10^–8^	4	–	–	–	> 600
GeS_2_ (PE-ALD) [94]	50	10^4^	~ 5	2 × 10^–8^	5	–	–	–	> 600
Ge–S–As [96]	60	10^4^	12	< 10^–8^	~ 2	~ 1.5	10	~ 10^10^	> 450
Ga–S [100]	60	10^4^	21.23	10^–8^	~ 2	~ 1.5	–	–	~ 500

## Switching Mechanisms of OTS

### OTS Models

Since the discovery of OTS materials, many models were developed to explain the threshold switching phenomenon [16, 29, 101–110]. For instance, Kroll et al. proposed a *thermal runaway model* [103, 104] that explains the OTS as the result of a Joule heating process, which triggers a positive feedback loop for carrier generation and leads to an exponential increase of the conductivity. Nevertheless, the thermal model could not capture one of the most relevant feature of OTS: negative differential resistance (NDR) [111, 112]. While the thermal models were successfully applied to describe the NDR in single-crystal Si-doped YIG [113], in crystalline, large-gap semiconductors the NDR is not the result of an ovonic threshold switch. By the early 1980s, the thermal model was overthrown by the electric-field-driven OTS models [101, 114] that will be discussed below.

**Fig. 24:**
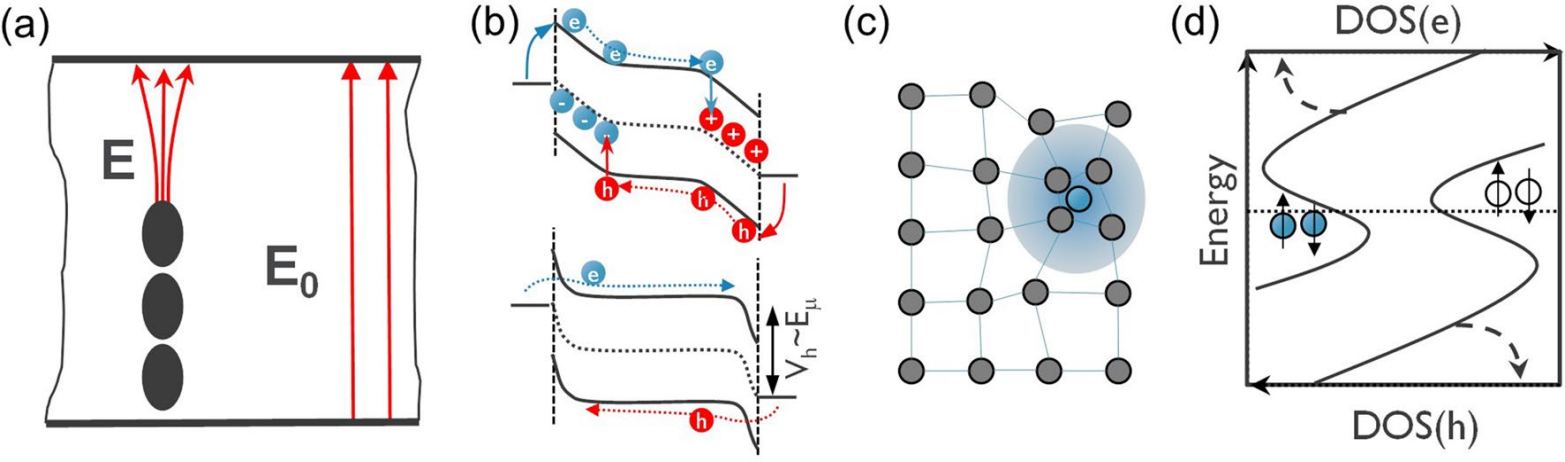
**a** Field-induced nucleation model—the nucleation of conductive crystallite is in a highly resistive amorphous matrix that concentrates the electric field and drives the filament growth until the device is being shunted. Adapted with permission from [107]. Copyright 2007, AIP Publishing. **b** Carrier injection model—*e/h* free carriers are injected on both sides, neutralizing VAPs, which do not interfere anymore with the electronic transport. Holding voltage corresponds to the mobility gap energy. Adapted with permission from [115]. Copyright 1973, IEEE. **c** Small polaron model—trapped charge carriers modify the local atomic bonding environment. This bond rearrangement modifies the local morphology of the materials and reduces the carrier mobility considerably. Above a critical quasi-particle density, the destructive interference of the atomic displacements does not hamper the carrier transport any longer. **d** Density of States (DOS) for the 2-electron and 2-hole vs. their one-particle energies in amorphous matrix, where rare, special, local negative-U centers showing strong polaron effects provide its Fermi level pinning. Adapted with permission from [110]. Copyright 2012, AIP Publishing

In PCM materials, OTS is the precursor mechanism of the ON switching that induces a local Joule heating process and the eventual crystallization of the amorphous into a high-resistance state phase. In this framework, Karpov et al*. *[105–107] introduced a *field-induced nucleation model* that assumes that the crystalline growth section, described as being a conductive cylinder in Fig. 24a, proceeds only once past a critical nucleus size of the filament. If the nucleus size is smaller than the critical size, the crystal seeds revert back into their amorphous phase upon field removal. This model is able to describe the drift dynamics of the threshold voltage upon device operation, highlighting the need to consider the amorphous matrix relaxation when dealing with drift dynamics. Its disadvantage is that it is not directly linked to the electronic properties of the material and hence does not properly account for its conductivity [23]. However, if one makes abstraction of the conductive cylinder/crystal grain/rod as to regard it as a (meta) stable (Ohmic) conductive local atomic percolation path, it contains an important ingredient for the universal OTS model; namely the bi/metastable state of the amorphous matrix and its associated relaxation time that defines the material parameter drift dynamics.

On the other hand, electronic switching models [102, 115–120] have been proved to be the most successful in describing the OTS conductivity and hence their associated threshold switching. In those models, the atomic bonding relaxation is not explicitly considered. However, many models consider the electron–phonon interactions or polaron relaxation implicitly. For instance, Ovshinsky [16] originally proposed an electronic mechanism at the origin of the threshold switching. The conduction was supposed to be phonon-assisted hopping near the Fermi level and not due to excited carrier conduction in the bands. The *carrier injection model* [115, 116] describes the conduction to be initiated when electrons and holes injected from cathode/anode compensate the positive/negative valence-alternation pairs (VAP) [101, 121–123] throughout the layer. The injected free carriers are transported in the mobility edges of the density of states. The holding voltage, therefore, reflects the mobility gap of the material (Fig. 24b), a correlation which was actually observed in a set of nine OTS materials [124]. Adler et al*.* [101] interpreted the threshold switching as originating from an impact ionization (II) process and formally described the NDR as being a competition between a carrier generation process (driven by both electric field and carrier densities) and a Shockley–Hall–Read (SHR) recombination. The model takes into consideration the electronic structure of the amorphous materials and together with the VAP theory [121], gives a detailed interpretation of the changes that (might) occur at the atomic/electronic level. It is worth noting that the model considers a local bond rearrangement/coordination change that takes place upon charge trapping on defects, which is a short-range (intimate valence–alternation pair) mechanism and does not require any atomic drift or diffusion when the material switches on or off. In other words, the impact of atomic relaxation upon defect charging was only implicitly hinted. Redaelli et al*.* [102, 117] improved this description by adding an avalanche-like multiplication phenomenon to the list of carrier-generation mechanisms.

Emin’s [125] *small polaron model* of subthreshold conduction was motivated by small measured Hall mobilities. From that perspective, the switching corresponds to the moment when the density of small polarons increases sufficiently to reach a steady-state threshold, namely when adjacent polarons show destructive interference and cancel-out the atomic displacements of other polarons (Fig. 24c), which breaks the carrier-phonon interaction. This model was challenged by high quality Hall measurements [126]. Also, since in the amorphous matrix the strong small-polaron interactions can occur at rare, special and local bonding arrangements [110], the polaron-like band transport is prohibited. Nevertheless, the model highlights the role of local atomic relaxations that impact on the electronic conduction and therefore on the OTS mechanism. For instance, the 2 electron-2 hole “intimate pairs” will pin the Fermi level (Fig. 24d) and the switching on can be successfully described by soft atomic potentials that exhibit the double-well potential signature with two states [110, 127].

**Fig. 25:**
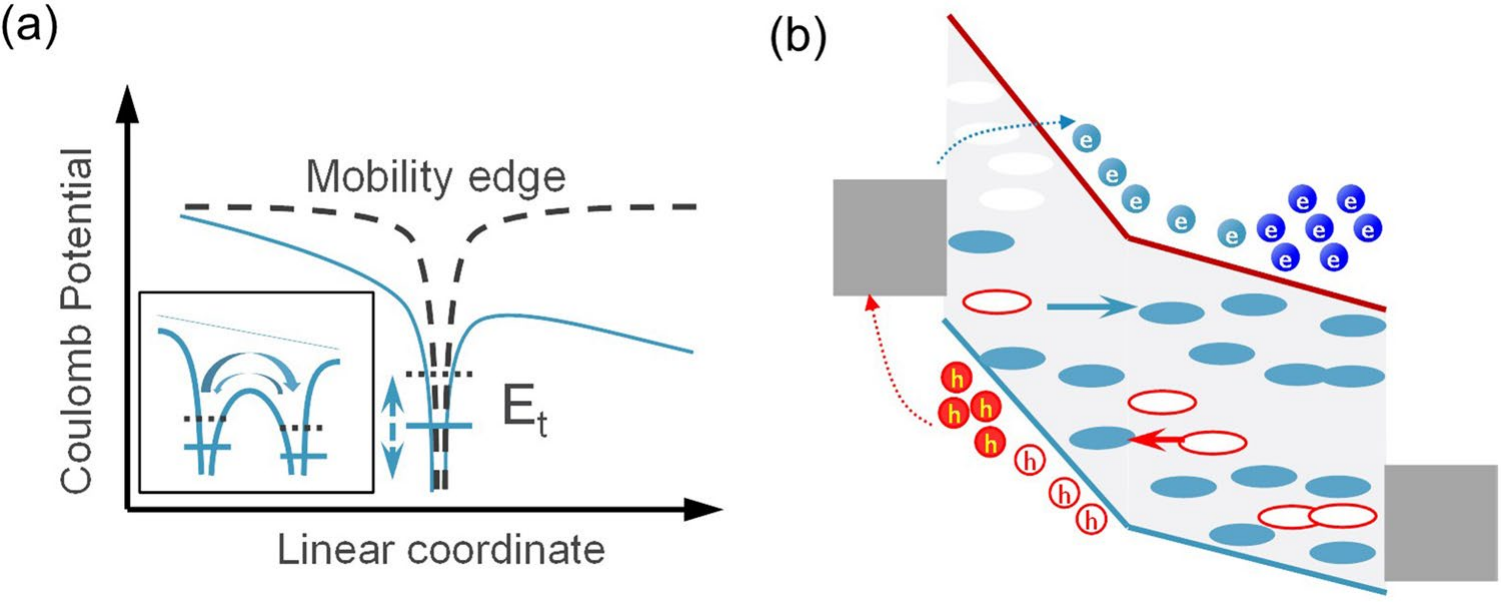
**a** Poole–Frenkel model capture-emission depends on the trap energy depth (relative to the mobility edges) and density (inset: denser traps interact and the emission barrier reduced not only by the field, but also by the trap proximity), local phonon-related relaxation energy (vertical dashed arrow) can also be accounted for. Emission time is exponential on the trap depth; therefore, the effective carrier mobility increases in the direction of the electric field. This simple model takes the average trap properties. Adapted with permission from [119]. Copyright 2008, American Physical Society. **b** At threshold field, the carrier injection results in a nonuniform internal electric field, higher at the cathode, non-equilibrium trap occupation, generation of secondary electrons with high mobility and the OTS can be sustained afterward, even at lower fields. Adapted with permission from [120]. Copyright 2023, John Wiley and Sons

Ielmini et al. [118, 119] introduced a *high-field Poole–Frenkel (PF) transport threshold switching model*, where the traps are located deep in the mobility gap (Fig. 25a) and trigger the switching of the *I*_*on*_ current at a certain non-equilibrium occupation of higher-energy traps. This results in a substantial nonuniformity of the electric field in the amorphous matrix. The model includes the implicit relaxation of the atomic matrix, irrespective of its physical origins (polaron relaxation or thermal vibrational contributions) to the trap energy through thermally-assisted hopping or tunneling mechanisms. The capacity of this model to describe the complete current–voltage signature contributed to its popularity.

A revisited version of the impact ionization and PF models was recently introduced by Fantini et al. [120] in a *bipolar avalanche model*, which describes the conduction as PF by majority holes at low electric field. These evolve into bipolar avalanche multiplication and ultimately the threshold switching is being triggered by this electron–hole cooperation, leading to a larger electron mobility compared to the hole one (Fig. 25b).

Buscemi et al. [108] introduced a *hydrodynamic-trap-assisted-tunneling (TAT) model* that considers the occurrence of temporary and progressive localization-delocalization of the states, creating conductive channels inside the insulating matrix. The transition rates are increased upon the switching voltage as function of the excess energy from the hydrodynamic-TAT model solution. Aside from unclear atomic-electronic interaction mechanisms, the last two models are capable to reproduce most (if not all) experimental measurements of the OTS devices.

Degraeve et al. [127] used a *two-state model* with a double-well potential to describe the metastable percolation path/cluster/channel in the threshold switching. This approach makes abstraction of the exact details of the physical mechanism, but allows for a completely analytical solution for any arbitrary defect cluster. Thus, the model is capable of capturing technologically important phenomena like cycle-to-cycle and device-to-device variations within an acceptable computation time. Apart from electronic effects, the two-state model also considers self-heating. In particular, the switch-off is identified as temperature-controlled.

In the following, first-principles evidences are provided to unravel the nature of electronic properties and the nature of mobility gap traps in amorphous OTS chalcogenide materials. Their interaction with the electric field is discussed and the accompanying polaron relaxation mechanism that occurs upon charge redistribution in the amorphous systems. Finally, a discussion on the most plausible OTS mechanisms is given.

### Electronic Structure of Disordered Materials

**Fig. 26:**
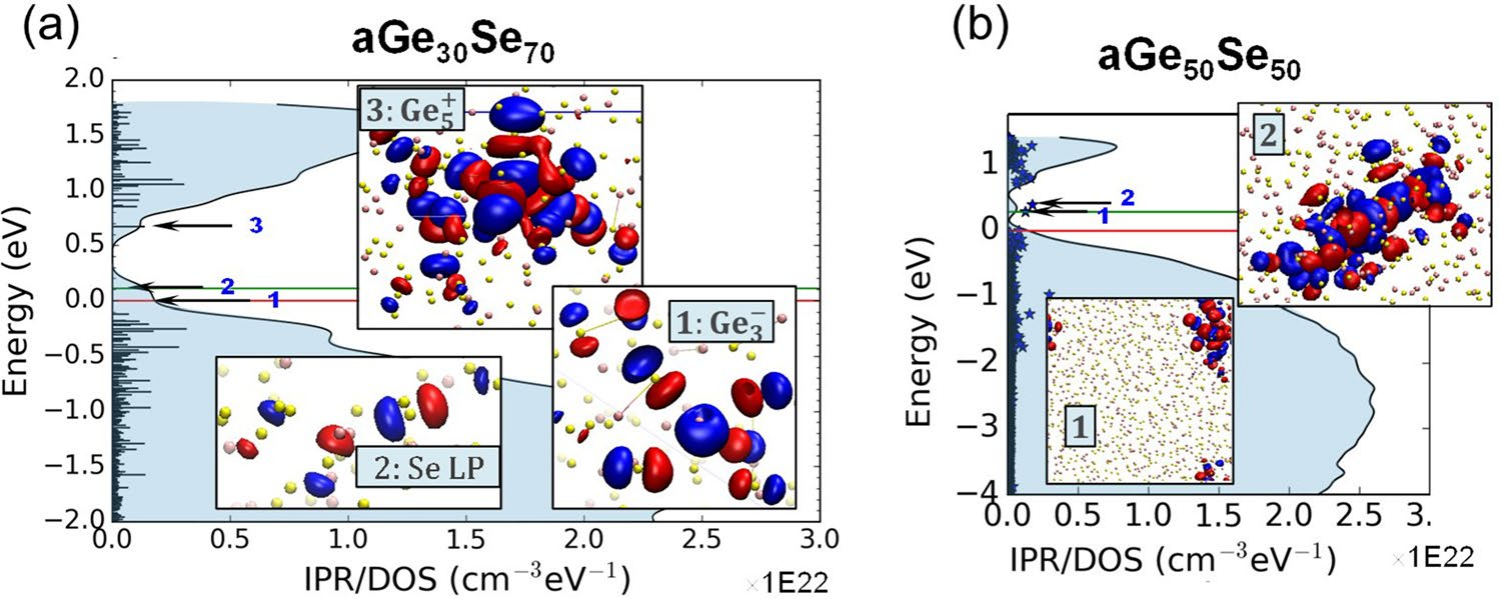
**a** Typical DOS in 2 × 2 × 2 nm Se-rich aGe_30_Se_70_ model with few Se-LP, Ge_3_^−^/Ge_5_^+^ VAP gap/tail states representations as insets. **b** Typical DOS in 3 × 3 × 3 nm Ge-rich aGe_50_Se_50_ model with few representations of gap/tail states. The red line denotes the Fermi level, the green line-the first empty state. The Se lone pairs and non-tetrahedral/co-linear bonds around Ge are typical for the mobility gap traps. Adapted with permission from [129]. Copyright 2020, John Wiley and Sons

In analogy to crystalline semiconducting materials, the electronic structure of glassy/amorphous semiconducting materials can be described by a gap between the valence and conduction (mobility) edges (in contrast to bands in crystals), with additional tail and/or gap states in-between that are localized in space. These gap/tail states can act as electron or hole traps (or defects) [128]. Traditionally, the electronic structure and possible transport mechanisms are discussed in terms of these electronic structure defects. Kastner et al. [121] introduced labels for the under-coordinated ${C}_{1}^{-}$ and over-coordinated ${C}_{3}^{+}$ chalcogenide atoms to represent the defect states that accepted/donated charges, calling them valence-alternation pair (VAP). In the same framework, over-/under-coordination can be extended to the elements of the group V (pnictogens 2P_3_ = P_4_^+^ + P_2_^−^) and IV (tetragons 2T_4_ = T_5_^+^ + T_3_^−^) [129–131] of the periodic table. The length of the trap localization is of the order of 1.5–2 nm [131]. This implies that atomistic models must meet a minimum size requirement. Typically, for density-functional-theory (DFT) simulations to be affordable, a model size of ~ 2 nm results in a trap concentration of ~ 10^21^ cm^−3^. This is a rather large concentration, compared to ranges measured of 10^18^–10^19^ cm^−3^. Despite this artificially high trap concentration, DFT simulations provide a glimpse at the electronic signature of the chalcogenide materials. Investigating various compositions of Ge_*x*_Se_*y*_, with x:y = [50/50 Ge-rich, 30/70 Se-rich], Clima et al*.* [129, 130] observed over-coordinated Ge_5_^+^ (electronic traps) and under-coordinated Ge_3_^−^ (hole traps) VAP states or Se non-bonding lone pairs (LP) (hole traps) localized states in Se-rich compositions (Fig. 26a), whereas in Ge-rich models there were mostly Ge–Ge chains of atoms, playing the role of electron/hole traps (Fig. 26b). It was shown that even within the fixed-atoms approximation, the electric field has a strong influence on the energy and localization length of the tail states.

**Fig. 27:**
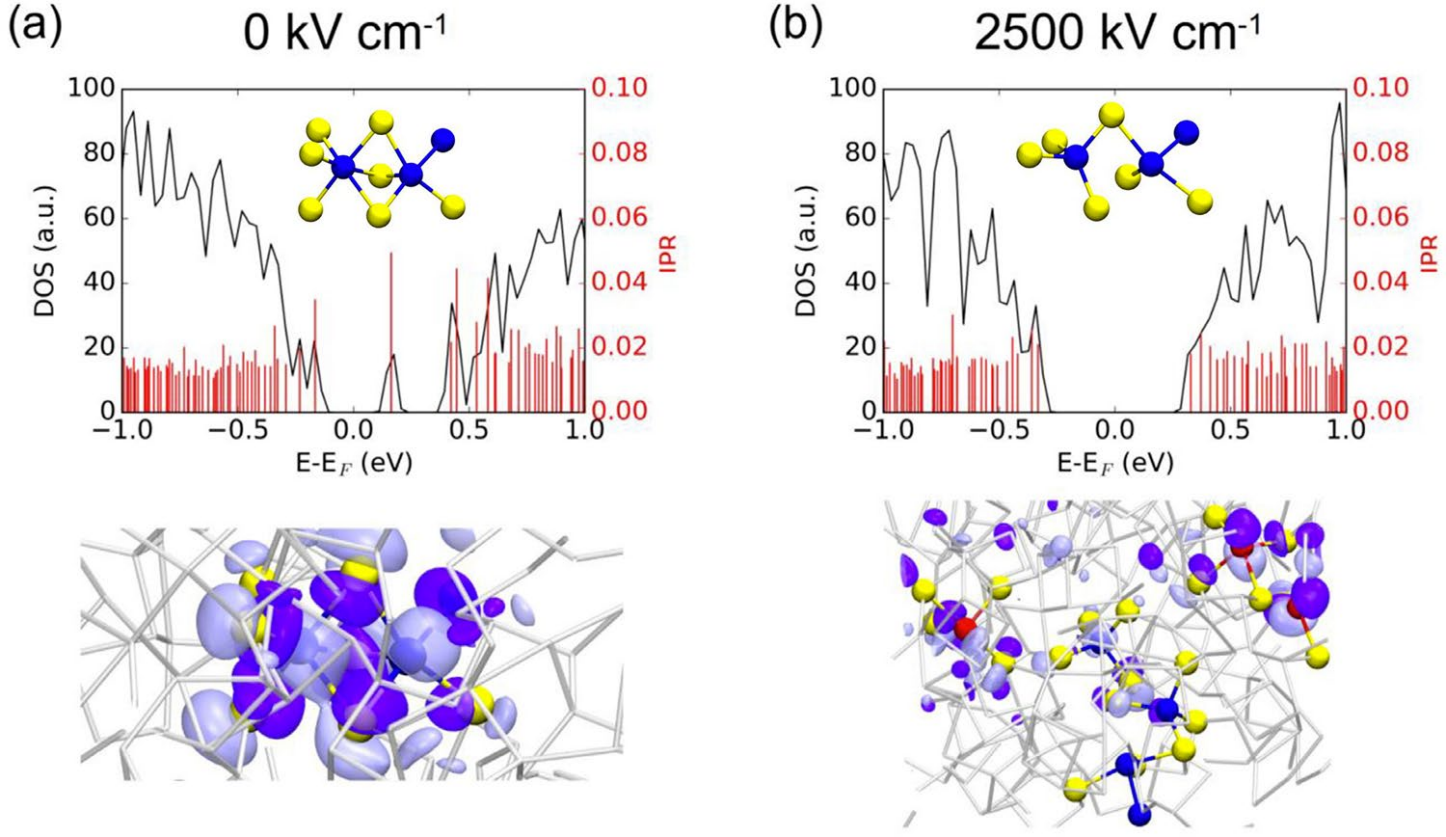
Density of States (DOS), Inverse Participation Ratio (IPR-degree of localization) and local atomic coordination for the **a** localized mid-gap state in the absence of electric field and **b** under 2500 kV/cm field. Adapted with permission from [134]. Copyright 2022, Elsevier

An applied electric field can promote electrons to higher energy states. If the higher-energy states are of antibonding character, the local bonding will be weakened and become unstable after this electronic excitation. The probability of encountering an electron promotion to the antibonding state is high in amorphous chalcogenides that have on average five valence electrons per atom (the 5 valence-electron rule [132, 133]). Konstantinou et al. [134] elaborated in details on the effect of the electric field on the mid-gap states in amorphous Ge_2_Sb_2_Te_5_ chalcogenide. It has been shown (Fig. 27) that 1–2.5 MV cm^−1^ applied electric fields are capable to break/reconnect and change the local coordination around the atoms hosting the localized mid-gap electronic state. This removes the trap from the mid-gap by transforming it into a delocalized conduction-edge state. This local instability is key in activating the OTS mechanism, which reflects the transition from insulating to conducting state of the material [135–137]. The work clearly shows that considering the effect of the electric field on the electronic structure defect in the gap (inherently linked to the local bonding/coordination) is required, when describing the OTS mechanism.

### Polaron Relaxation

At this level, it is interesting to understand what happens to the gap defect levels when they are getting populated. An important thing to describe the charge transport mechanism is to consider the atomic relaxation that inherently occurs during trap (de)occupation process. Nardone et al. [110] gave an elegant description of the strong interaction between the charged carriers and local atomic bonding in chalcogenide glasses. They argue that a high DOS in the mobility gap indicates that localized states in the mobility gap could provide with sufficient screening of the external field by forming a dipole layer by means of a redistribution of localized electrons [110]. This redistribution is accompanied by a set of local atomic deformations, similar to the lattice polaron relaxation but spatially localized in the amorphous matrix. In terms of atomic bonding, it can be pictured as if certain weakly bonding atomic morphologies are forming small “liquid-like” regions that got “frozen” in the solidified glass. In another context, these regions are known under the name of excitation lines or space–time bubbles [138]. Experimental evidences are pointing to the negative correlation (Hubbard/U [122]) energy nature of the amorphous chalcogenides. In other words, these doubly-occupied localized states are stabilized energetically by a polaron relaxation mechanism and single occupation exists only as excited states. These are supported by the lack of ESR signal at non-cryogenic temperatures. The Fermi level pinning with an activation energy for the conduction that is half the mobility gap supports the view that a strong polaron interaction is characteristic to the chalcogenide glasses that sets them apart from other amorphous semiconductors [110]. All these factors favor the 2e/2 h excitation model for the polaron conduction, that can well be described by a two-state model with conduction through a percolation channel [127].

Although unequivocally proving that the strong polaron interaction is responsible for electronic transport is challenging, we want to highlight in the following the importance of local structural relaxations upon trap charging by giving a few supporting theoretical findings. Raty et al*.* [137] studied with first-principles electronic excitations of the OTS glasses. Their results showed structural reordering, bond alignment, and appearance of local structural motifs that are characteristic of meta-valent bonds [138–140]. Next, the Born effective charges (BEC) of certain atoms increase drastically upon electronic excitation. These changes lead to large DOS/dielectric constant changes, hence reflecting a modulation of the conductivity of the material [141]. Noé et al*.* [136] argue that the bond alignment/delocalization is expected to introduce more conductive channels at the Fermi level of the amorphous OTS materials. Based on a BEC increasing upon trap charging, an OTS-gauge factor was defined to assess the material’s ability to undergo a threshold switch for theoretical screening purposes [98]. Another illustration of the impact of the polaron relaxation model in amorphous Ge_50_Se_50_ was given by Slassi et al*.* [142] whose results stressed the dramatic changes in the mobility gap undertaken by the amorphous matrix.

These models illustrate that atomic relaxation mechanisms are driving part of the electronic structure change taking place during the threshold switching process. That, together with the thermal Joule heating effects during switching or longer time operation of the device, leads to bond readjustments and therefore to a relaxation of the amorphous matrix with an eventual resistance/threshold voltage drift [105, 129, 143].

### Conduction-Switching Mechanisms Discussion

*Off-state conduction:* Nardone et al. [110] analyzed various conduction mechanisms in amorphous chalcogenides. They identified, in combination with experimental data, the most plausible electronic conduction mechanisms, namely: Poole–Frenkel ionization [118–120], field-induced delocalization of tail states, optimum channel hopping in thin films, optimum channel field emission, percolation band conduction [127], or transport through crystalline inclusions. The latter might be relevant for PCM materials, but it can safely be excluded for non-crystallizing OTS materials. Next, space-charge limited currents are only plausible in thick films and low temperatures conditions, whereas Schottky emission and classical hopping conduction (tunneling from trap to trap) were deemed very unlikely to occur. Finally, the multi-phonon trap-assisted tunneling (TAT) [108] was successfully used in describing the off conductivity.

*Switching:* electronic models describe the switching process as being a non-equilibrium condition for the trap occupation that alter the electronic structure [101, 102, 115–120]. A more physical description of the threshold switching mechanism requires considering both electronic and ionic dynamics events: when the mobility gap defects/traps get populated, a local atomic bond alignment/rearrangement (polaron relaxation) leads to different trap position in energy and to a spatial (de)localization [110, 129, 131, 136, 137, 139, 144]. As a consequence, the charge transport properties change dramatically, but reversibly. Most OTS switching models describe the same phenomenon at different abstraction level using different frameworks. For instance, the small polaron model describes the onset of the conduction at the polaron density that leads to destructive interference of atomic bond deformations of nearby polarons, whereas in PF model, the same phenomenon can be described by energy-aligned close in space traps with small hopping barrier that would effectively transform the traps into a delocalized sub-band. Also, the phonon-assisted hydrodynamic-TAT model considers the local polaron relaxation with the energetic load-relaxation of the traps upon charging, whereas the two-state model accounts for it with a double-well potential between localized and charged/relaxed/delocalized states. The level of description is a trade-off between physical precision and computing speed. Important practical phenomena like device-to-device variability can only be captured when abstraction of the atomistic mechanism is taken. Consequently, it is not straightforward to dismiss certain models over others since they are all mostly complementary to each other, if not the same on the grand scale. While one could argue that many aspects of the conductivity and switching are accurately described mathematically by selecting one or combining several models (see, for instance, Fantini et al*.* [120] for the latest/ most comprehensible model on OTS switching), there is no definitive proof of the exact physical mechanism behind the phenomenon. It could also be that several different conduction mechanisms are active at different electric fields or in different materials. The exact atomic/electronic physical mechanisms are still difficult to prove experimentally and claim a full understanding of.

*On-state conduction:* In terms of spatial distribution of the current, some models treat the conductivity to occur in confined space of the device (filamentary/percolation), while others consider the conduction to be bulk-limited [118, 119]. However, when the device size is reduced, the bulk mechanism can be applied successfully to the confined space as well. Percolation conduction (optimum channel hopping, (sub)band conduction in variable trap densities) is naturally suiting amorphous materials with a conductivity field dependence as that of the PF conductivity [110]. Next, concepts based on a band conduction at the (modified) mobility edge or an impact ionization/avalanche multiplication also describe well the *on*-state conductivity. Indeed, the *on* state is not stable in absence of a flowing electrical current; as a consequence, the trap occupation state and delocalization are determined by the trapping/de-trapping dynamics. Then the rate of incoming electrons to maintain an average-full state of all the traps in the material is not sufficient, the atomic configuration around the trapping sites relaxes, leading to a charge localization and breaking the conductive cluster/percolation path/channel.

To summarize, in the subthreshold regime there is a trap-limited current that can be described by more than a single plausible physical mechanism. Electric field and charge injections affect not only the occupation, but also the localization in space and in energy of the gap traps. At threshold switching, a non-equilibrium trap occupation (possibly also delocalization) occurs, which leads to a metastable state of the (cluster of) traps that conduct the carriers leading to either a (sub)band charge transport and/or also to an impact ionization/avalanche multiplication. A mix of both electronic and hole types of conductivities is likely to take place. The metastable state of the traps is retained by the electrical current until it is not sufficient to maintain them in the high-energy state (charged state), since the de-trapping rate is out of equilibrium with the trapping one and the system reverses back to the initial state (uncharged traps). Thermal effects on atomic relaxation are important ingredients in changing the conductive properties of the percolation path in the OTS material, which define the long-term evolution of the conductive path (aging, endurance, parameter drift).

## Applications of OTS

When OTS devices were initially proposed in 1960s, their primary application purpose was to serve as a new type of rectifier device, aiming to replace conventional diodes [20, 20, 101]. However, due to challenges related to material and fabrication compatibility, OTS selectors did not gain significant advantages over diodes in the early stages of development. The latter, together with MOSFET, dominated the electrical semiconductor in the latter half of the twentieth century. Entering the twenty-first century, as the semiconductor process node advanced, conventional diode and MOSFET-based memory technology, that is, DRAM and Flash, nearly approached the physical limit, high-density emerging memories thereby were highly desired. The OTS device, as a promising selector unit in high-density arrays, regained wide research attention and ultimately achieved successful industrial applications in 2017 [11]. Besides being a switching selector, the recently reported OTS-only self-selecting memory in 2021 is a milestone for the OTS device, aiming to achieve low-cost and high-density memory [145]. Moreover, with the rise of the concepts such as neuromorphic computing and artificial intelligence (AI), there is an increasing interest in exploring the hardware platforms and devices that serve as carriers for computational theories [146]. The OTS device, with their excellent device consistency and superior scalability, also holds significant value in the field of neuromorphic computing. In this section, these potential applications of the OTS device are discussed in detail.

### 3D Memory

With the deepening research on next-generation fast-speed and high-density memories, the application of OTS in 3D novel memory, particularly in 3D PCM, has gradually become one of the major directions for OTS devices. The remarkable advantage of OTS, with substrate-free two-terminal structure, lies in its extremely high area utilization. Compared to MOSFET (> 8F^2^) and bipolar switches (5F^2^), the combination of OTS and PCM in a 1S1R configuration can achieve 4F^2^, which is a critical requirement for 3D high-density stacking-a key aspect of miniaturization. Over the past 20 years, PCM technology not only underwent aggressively size scaling from 180 nm to 20 nm, but also experienced a revolutionary evolution of the selector cell from the initial silicon-based MOSFET and bipolar junction transistor (BJT) to the OTS technology, successfully enabling the increasing capacity from 4 Mb to 256 Gb [11, 12, 25–28].

**Fig. 28:**
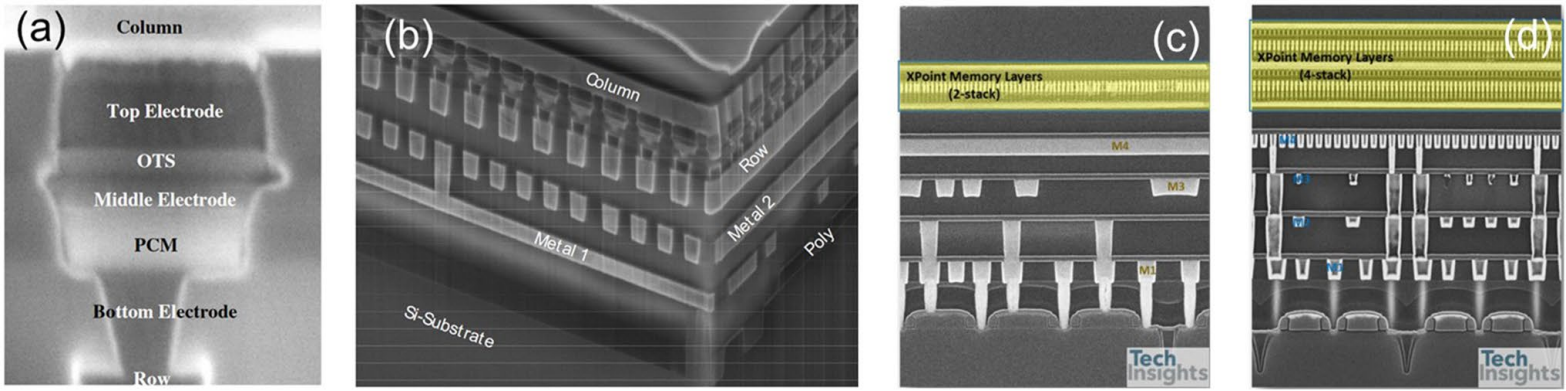
1S1R structure in PCM. **a** Vertically integrated memory cell of 1S1R. **b** One layer of the PCM array fully integrated with CMOS [11, 12]. Adapted with permission from [11, 12]. Copyright 2009/2020, IEEE. **c** First-generation 2-deck 3D Xpoint array. **d** Second-generation 3D XPoint with 4-deck array [147]

In 2009, Intel’s research team first reported the 1S1R 3D stacking technology [11] (Fig. 28a, b). In August 2015, Intel and Micron jointly announced the 3D Xpoint chip based on “bulk change” technology (a novel PCM device structure). In 2017, Intel introduced the Optane series chips based on 3D PCM technology to the market, including Optane solid-state drives (SSD) for the enterprise market and Optane flash memory for the consumer market. These chips were manufactured using mainstream 20-nm process technology, with a two-layer stacking. The optimized Ge–Se–As–Si OTS materials was believed to be used in the PCM-based Optane, achieving a storage density of 0.62 Gb mm^−2^ (Fig. 28c). This density was approximately 4.5 times higher than that of DRAM at the same 20-nm process node, with 91.4% of the core area occupied by the memory array [147]. In October 2019, Micron introduced the X100 NVMe enterprise SSD based on 3D PCM technology. Currently, 3D PCM with cross-point memory array has achieved four layers and 256 Gb per die in the second generation (Fig. 28d). The two generations of Intel’s Optane memory, which are based on the 3D Xpoint structure, are the outstanding application of OTS devices. Although the next generation of Optane memory will not be introduced in the short term due to commercial and market reasons, the successful commercialization of the two generations of Optane memory serves as strong evidence for the application of OTS devices in memory technology.

Process complexity and cost are the main factors behind the current suspension of 3D Xpoint business. Moreover, as the number of stacking layers continues to increase, the cost per bit will significantly increase rather than decrease, due to the high dependence on lithography. The persistently high cost issue will be one of the challenges that the 3D Xpoint structure will inevitably face in future [148]. Therefore, the concept of using vertically integrated “true” 3D structures for high-density storage is gradually gaining attention. Although there is currently no fixed standard for vertical memory architecture, we can be certain that in vertical structures similar to 3D NAND, OTS materials will continue to play a role as the “select layer” integrated with the memory cell [149, 150]. Furthermore, the OTS device can be applied not only to PCM but also to other emerging memories, like resistance random access memory (RRAM), and still holds great application prospects in 3D storage technology.

### Self-Selecting Memory

**Fig. 29:**
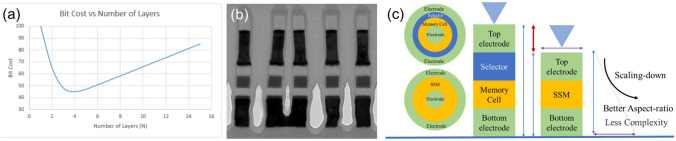
**a** The bit cost versus number of layers [148]. **b** An example image of leaning and the resulting bridges in 3D XPoint structure. Adapted with permission from [151]. Copyright 2022, IEEE. **c** Concept of SSM (Self-Selecting Memory) in vertical and plane structure

As aforementioned, the 3D Xpoint structure will face increasing manufacturing costs as the number of layer increase (Fig. 29a) [148]. This issue could potentially be addressed by adopting vertical architecture. However, it is foreseeable that for the current 1S1R structure, there are inherent process difficulties in precisely controlling the composition and ensuring atomic-level homogeneity in the film. In addition, the traditional 1S1R structure requires a memory cell to be connected in series with an independent selector, resulting in a small overall aspect ratio, which will bring about process problems such as “leaning” involving the overall strength of the device as the process node scales down (Fig. 29b) [55, 152, 153]. To address these issues, a new technology path known as “self-selecting” has emerged, aiming to converge the selector and memory into a unit without the need for another separate device [154–157]. Memory devices based on this architecture are referred to as “self-selecting memory (SSM)” (Fig. 29c). This concept goes beyond the self-select of the memory cell in 0T1R structure and includes the implicit concept of self-memory in the 1T0S structure. To realize this technological concept, the measurable parameters used to represent the stored data must be designed to ensure the stability, repeatability, and a large enough difference between two (or more) states to enhance the data retention and maintain the access window.

**Fig. 30:**
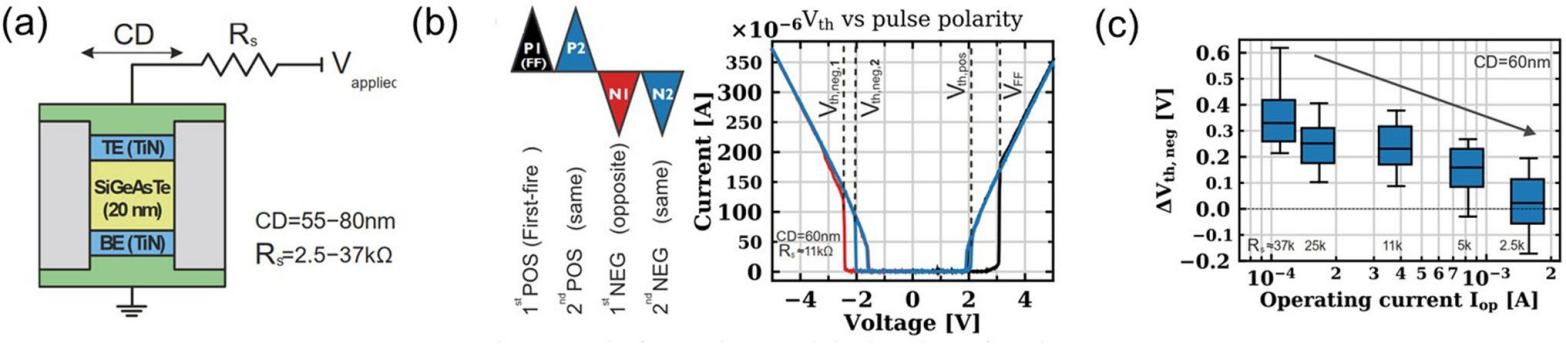
Ge–Te–As–Si OTS-based SSM. **a** Device Structure. **b** *I*–*V* curves, indicating the polarity effect in OTS device. **c** Negative versus *I*_op_ with different resistance, the Δ*V*_th_ increased with the resistance rising [145]. Adapted with permission from [145]. Copyright 2021, IEEE

The OTS selector, being typical volatile device, is impractical for storing data using the conventional binary *on/off* states. Interestingly, *V*_th_ in OTS selector were found to be influenced by many factors such as device structure variations, the amplitude of applied pulses [158], pulse width [159], ramp rate [160], and relaxation time [161]. Ravsher et al*.* in late 2021, observed the control of the *V*_th_ of OTS device (composed of Ge–Te–As–Si) by the polarity of the applied voltage, more specifically, a systematic and stable changed with the alterations in polarity of the applied voltage (Fig. 30) [145]. The value of *V*_th_ in the current operation was closely affected by the polarity of the previous pulse, resulting in the generation of two relatively stable *V*_th_ levels, *V*_th1_ and *V*_th2_, within the same pulse direction. In this work, the negative polarity *V*_th_ difference (Δ*V*_th_ =|*V*_th1_−*V*_th2_|) was 260 mV, while the external resistance increased, further enhancing the Δ*V*_th_ (Fig. 30c). Exploiting this stable Δ*V*_th_, which could persist for at least 1000 s (~ 17 min) as reported, enabled the independent utilization of OTS device for binary storage device, called OTS-only SSM.

**Fig. 31:**
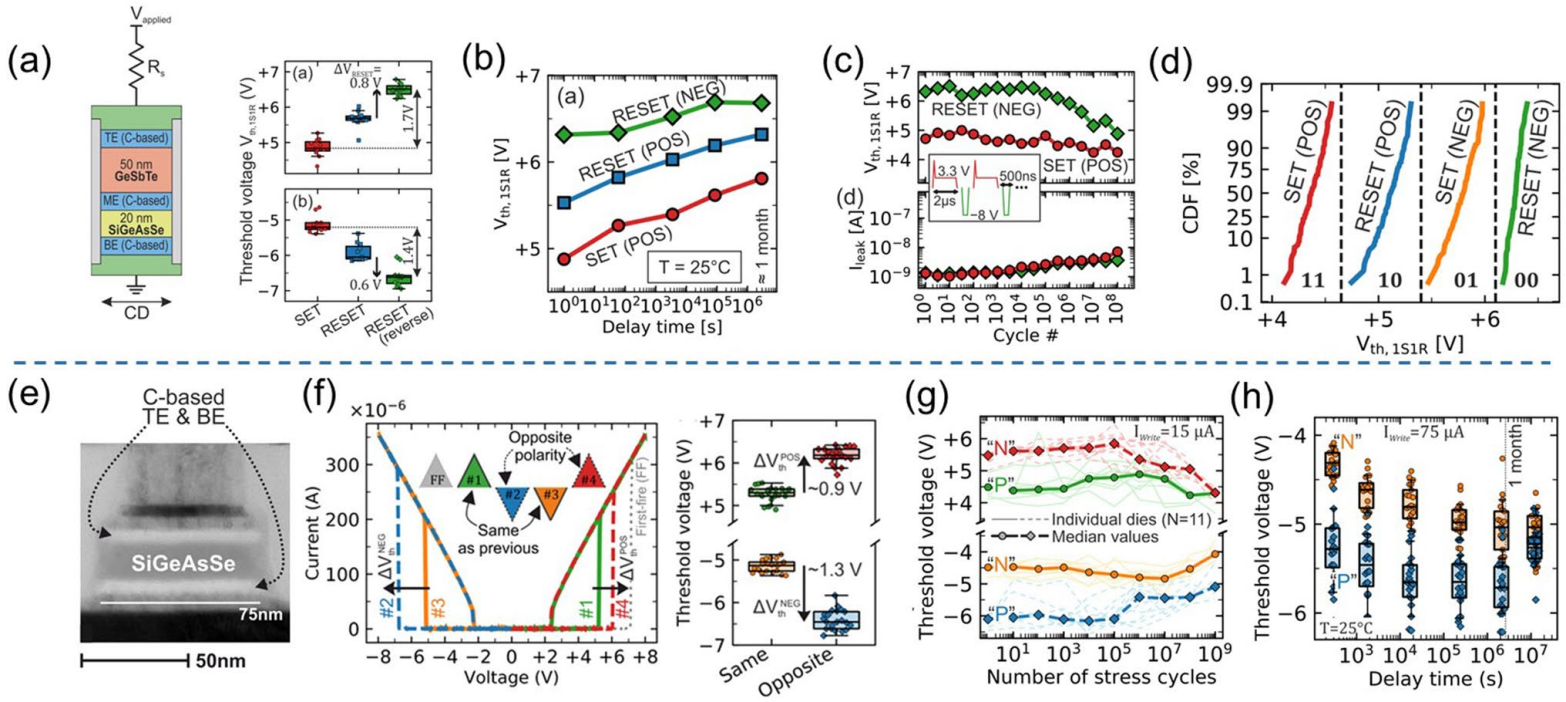
Ge–Se–As–Si 1S1R cell and SSM. **a** Structure of the 1S1R cell and the distribution of its margin window. **b** > 1-month Retention. **c** Endurance over 10^8^ cycles. **d** Four storage levels induced by the polarity effect. Adapted with permission from [162]. Copyright 2022, IEEE. **e** SSM with 75 nm feature size. **f** *I*–*V* curves and the distribution of negative and positive Δ*V*_th_ [162, 163]. **g** Endurance characteristics under bipolar stress. **h** Retention measurement. Adapted with permission from [163]. Copyright 2023, IEEE

Subsequently, the research team reported on the optimized device in mid-2022 [162], focusing on their application in the 1S1R structure. The device employed Ge–Se–As–Si OTS material. The improved device exhibited a further increase in the Δ*V*_th_, ~ 1 V, and the positive Δ*V*_th_ became more pronounced. The SSM, which was in series with a Ge–Sb–Te PCM cell (Fig. 31a), aimed to enhance the performance of traditional 1S1R cell by offering double SET states and RESET states, each with a distinct difference. By employing voltage operation with different polarities, the originally small margin window (0.85 V) was effectively enlarged to ~ 1.7 and ~ 1.4 V. The 1S1R cell demonstrated a rather long retention reaching up to 1 month @ 25 °C (Fig. 31b) and an endurance over 10^8^ cycles (Fig. 31c). Additionally, the significant difference among the four states indicated the potential for multi-level storage with four resistance states and even more (Fig. 31d). If the PCM itself has the potential to achieve multi-level storage, then the polarity operation of OTS devices may enable doubling of the storage states.

Recently, Ge–Se–As–Si-based SSM device with C-based electrodes was reported by the same research team [163]. The device exhibited a larger positive Δ*V*_th_ of ~ 0.9 V and negative Δ*V*_th_ of ~ 1.3 V (Fig. 31f), an ultralow writing current < 15 µA and a high endurance > 10^8^ cycles (Fig. 31g). However, the SSM device showed some limitations in data retention. With a write operation performed at 75 μA, the window could be reliably maintained for 1 month @ 25 °C (Fig. 31h), and nearly disappeared after five months. If a lower operating current ~ 15 μA was employed, the data retention of the device further decreased to ~ 10 days, indicating that the SSM device cannot currently be considered a typical non-volatile memory without compositional and structural optimizations. However, it can be utilized as a “DRAM-like” memory with a longer refresh period, and the non-destructive “Read” operation can be used for refresh operation.

**Fig. 32:**
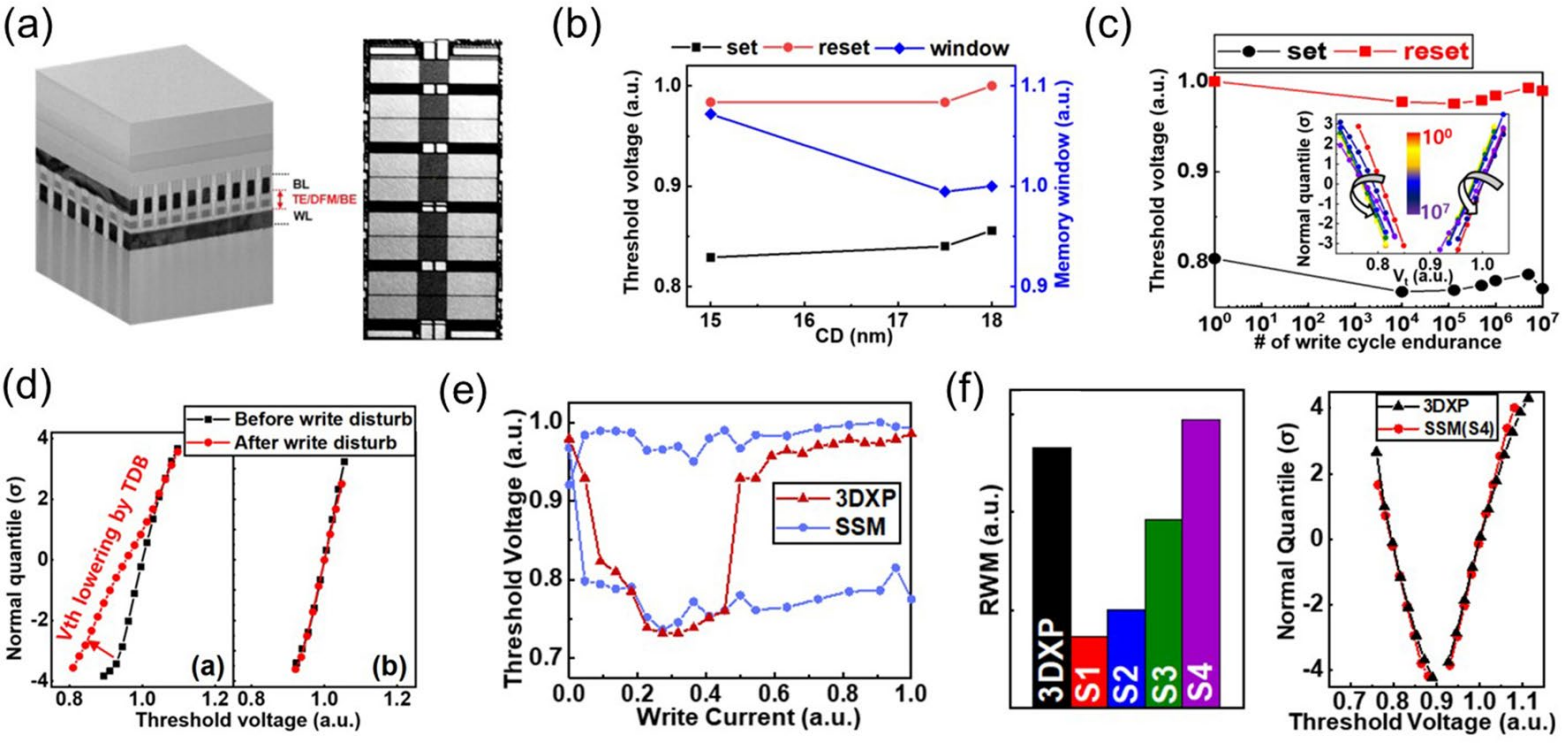
SSM prototype. **a** Structure of the SSM array. **b** Variations of *V*_th_ and the memory window with the device scaling down. **c** Endurance and the distribution of *V*_th_. **d** Distribution of *V*_th_ before and after the driven thermal disturbance. **e** *V*_th_–*I* curves between 3D Xpoint and SSM. **f** Comparison of the margin window with 3D Xpoint [151]. Adapted with permission from [151]. Copyright 2022, IEEE

In 2022, Hong et al*.* [151] from SK Hynix fabricated a prototype of a 32 Mb SSM (Fig. 32a), strongly confirming the feasibility of engineering SSM for practical applications. The simplified device structure provided a larger aspect ratio, enabling it to maintain a good switching window even at the 15-nm process node (Fig. 32b). The device exhibited high endurance exceeding 10^7^ cycles (Fig. 32c) and a rather low *I*_op_ (Fig. 32e). The unique structure of the SSM effectively suppressed the increasingly significant thermal disturbance in downscaled PCM (Fig. 32d). Through four generations of product iterations, the margin window of the prototype has slightly surpassed that of PCM in 3D Xpoint (Fig. 32f).

In summary, the innovative SSM technology offers a promising path for future application of the OTS device. Additionally, the endurance of OTS devices, resulting from their switching behavior, replaces the repetitive amorphous–crystalline phase transition in PCM, leading to significantly improve the endurance. Furthermore, the use of OTS device’s threshold current/voltage instead of PCM’s operating current/voltage eliminates the need for melting the chalcogenide material, resulting in a notable reduction in energy consumption.

### Neuromorphic Computing

In the traditional von Neumann architecture, which separates storage and computation, there is an inherent “memory wall” problem, which becomes more pronounced as the process node scales down [5, 164]. With the recent resurgence of AI technologies, a series of bio-inspired neural computing architectures have gathered significant attention due to their potential for achieving ultra-low power consumption and large-scale, highly parallel and high-speed computing. The neural computing platform and its devices, as the physical embodiments of these neural computing architectures, have naturally become a focal point of research. The physical realization of artificial neurons, synapses, and other bio-inspired devices is one of the key challenges in neuromorphic computing [165–172]. Mainstream novel computing architectures include spiking neural networks (SNNs), deep neural networks (DNNs), and in-memory computing. For a long time, the physical implementation of these computing architectures relied on combinations of CMOS and non-volatile memory (e.g., PCM, RRAM) [165–168, 173–175]. Early on, OTS played a role as a selector, primarily in combination with the memory, to suppress leakage currents, reduce power consumption, and restrain crosstalk, which is particularly crucial in large-scale arrays [146]. However, selector unit did not directly serve as artificial neurons and synapses themselves at that time.

The spiking neural networks exhibit remarkable potential in reducing the computational energy consumption due to their event-based and data-driven nature. However, the lack of a general learning rule hampers their ability to construct the large-scale networks. Recent reports suggest that SNNs can be constructed by transforming pre-trained artificial neural networks (ANNs). In this transformation, the neuron units are simplified to integrate and fire (I&F) neurons, which integrate input synaptic current and charge the membrane potential [176–179]. Once the membrane potential reaches the threshold voltage, the neuron generates spikes to the next synapse layer and resets the membrane potential. This characteristic aligns well with threshold switching devices [169], as traditional CMOS-based neurons suffer from large size and high power consumption [180–183].

**Fig. 33:**
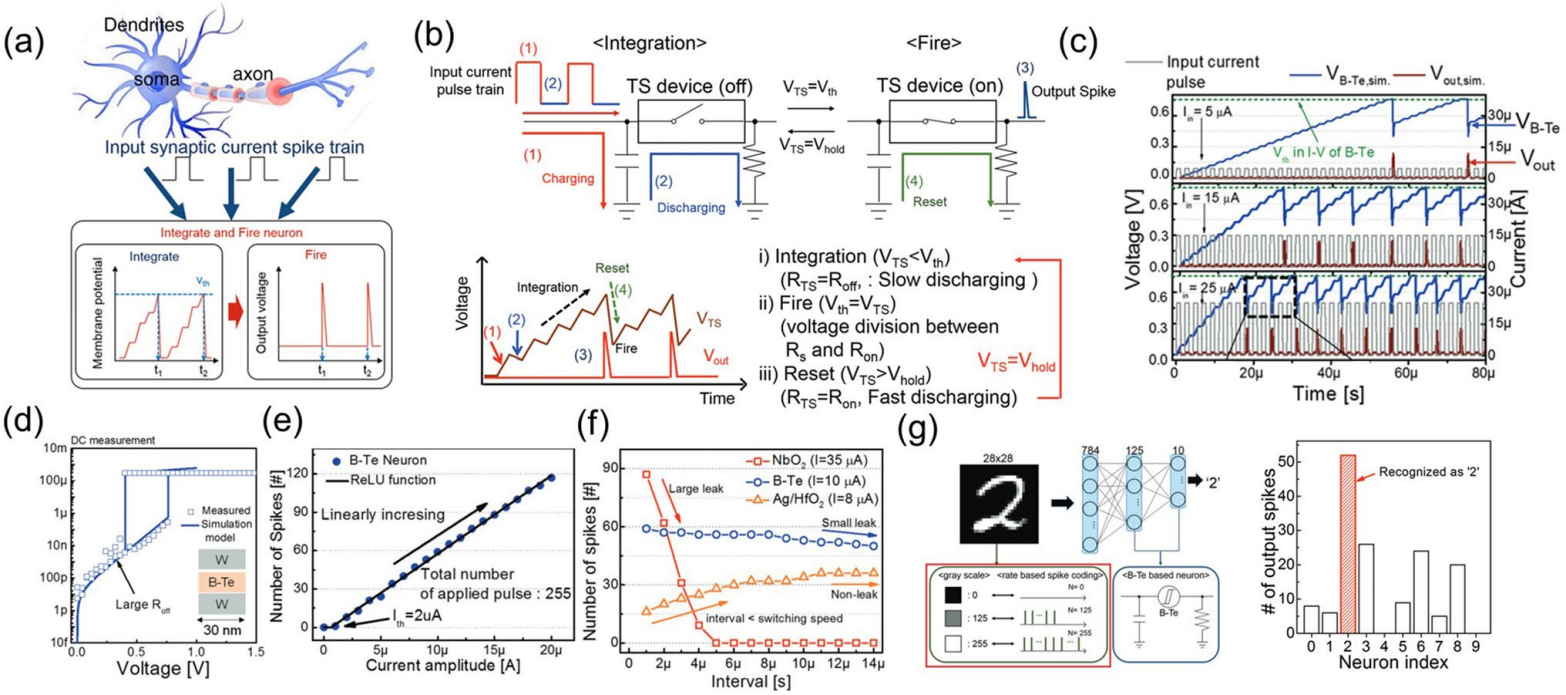
Integrate and fire (I&F) neuron based on B–Te. **a** Concept of integrate and fire (I&F) neuron. **b** Schematic and Operation principle of I&F neuron. **c** *V–t* curves of neurons based on B–Te under different input current. **d** *I*–*V* curves of B-Te; **e** Number of spikes versus input current amplitude in B–Te selector, indicating the typical ReLU function. **f** Number of spikes as a function of pulse interval. **g** MNIST classification and the result of recognition [66, 184]. Adapted with permission from [184]. Copyright 2019, John Wiley and Sons

Hence, the selectors, including NbO_2_-based MIT devices, B–Te-based OTS devices, and Ag/HfO_2_-based ionic-diffusion devices, have been investigated by Lee et al. as potential components for I&F neurons [184]. The study revealed that the B–Te OTS device exhibited excellent comprehensive performance, showcasing a low *I*_off_ of ~ 5 nA (Fig. 33d) and a short switching time of < 10 μs. These features contribute to improved leakage behavior and signal responsiveness in the operation of I&F neurons based on B–Te OTS devices. The relationship between the output pulse count and input current of the B–Te OTS device followed a typical rectified linear unit (ReLU) function (Fig. 33e). Additionally, the device demonstrated a higher operating bandwidth and more stable response characteristics in the relationship between output pulse count and input signal frequency tests (Fig. 33f). Remarkably, the B–Te OTS device also exhibited outstanding energy efficiency, with power consumption reducing to 30 pJ per spike. Subsequently, the research team employed I&F neurons based on B–Te OTS devices to process frequency-encoded ANN data in an SNN for handwritten recognition tests, achieving an acceptable recognition rate ~ 33% (Fig. 33g).

Lee et al. reported an artificial neuron device that consisted of an OTS selector and a few passive electrical components, capable of mimicking various behaviors of a biological neuron, including I&F, rate coding, short-term plasticity (STP), and chaotic activity [185]. They utilized a Ge–Se OTS device to construct an I&F neuron for testing, which exhibited favorable spike-frequency adaptation. Additionally, they documented the device’s response characteristics in a chaotic system. The energy consumption per spike was estimated to be approximately 1.2 nJ per spike. Subsequently, by combining the OTS-based neuron device with reservoir computing techniques, they successfully performed spoken-digit recognition tasks with a significant level of accuracy ~ 94%.

**Fig. 34:**
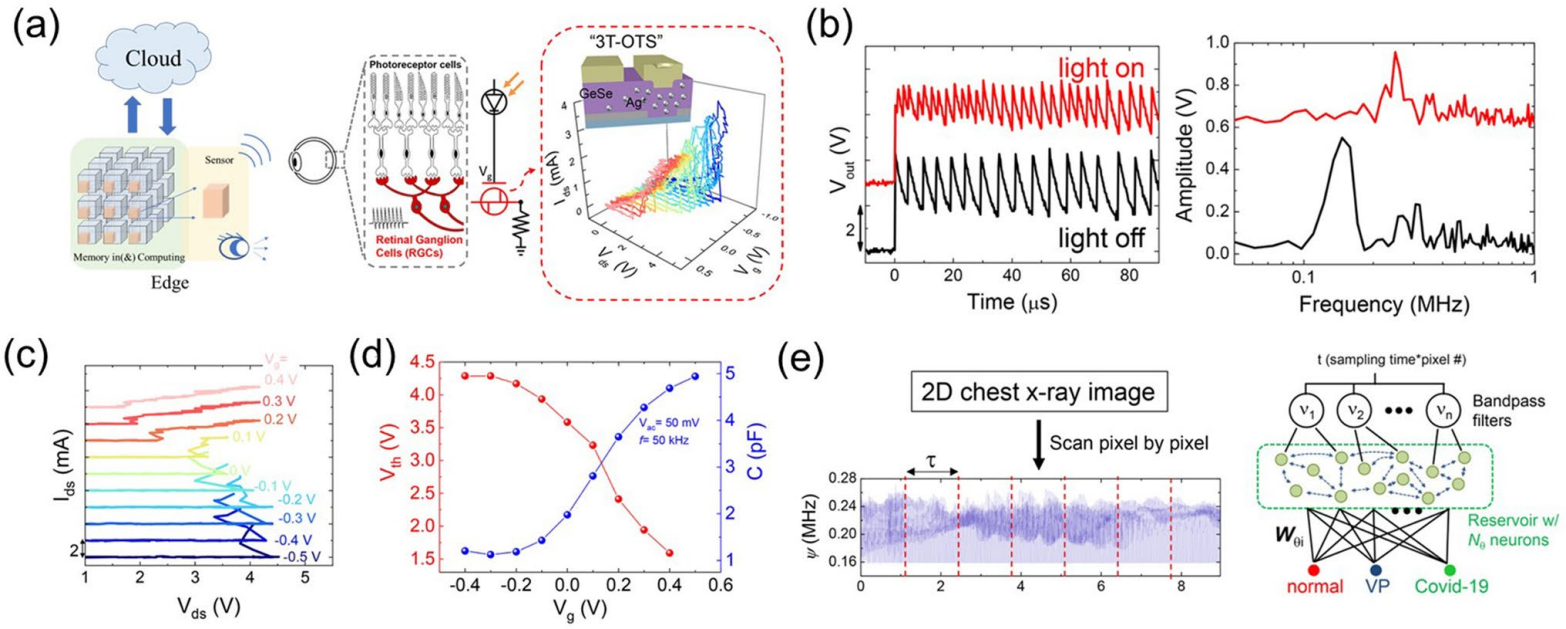
3 T-OTS device as an artificial RGC. **a** Concept of edge- and cloud- computation, the diagram of RGC and an artificial RGC. **b** Response waveform of an artificial RGC with/without illumination, and the FFT spectra. **c** Variations of *V*_th_ under the modulation of *V*_g_. **d** *V*_th_ and *C* versus *V*_g_. **e** Classification of chest X-ray images between normal and other virus [189]. Adapted with permission from [189]. Copyright 2022, American Chemical Society

The significance of sensing, memory, and computation integrated technology is increasingly evident [186–188]. In this domain, OTS device also found its potential applications. Lee et al. reported on the development of artificial sensory neurons based on a novel three-terminal OTS (3 T-OTS) [189]. The device utilized the Ge–Se–Ag system and allowed for *V*_th_ modulation through gate control. By integrating with a photodiode, they constructed an artificial retinal ganglion cell (RGC) (Fig. 34). This artificial RGC, combined with the reservoir-computing technique, was employed for classifying chest X-ray images into normal, viral pneumonia, and COVID-19 infections, achieving a recognition accuracy of approximately 86.5%.

**Fig. 35:**
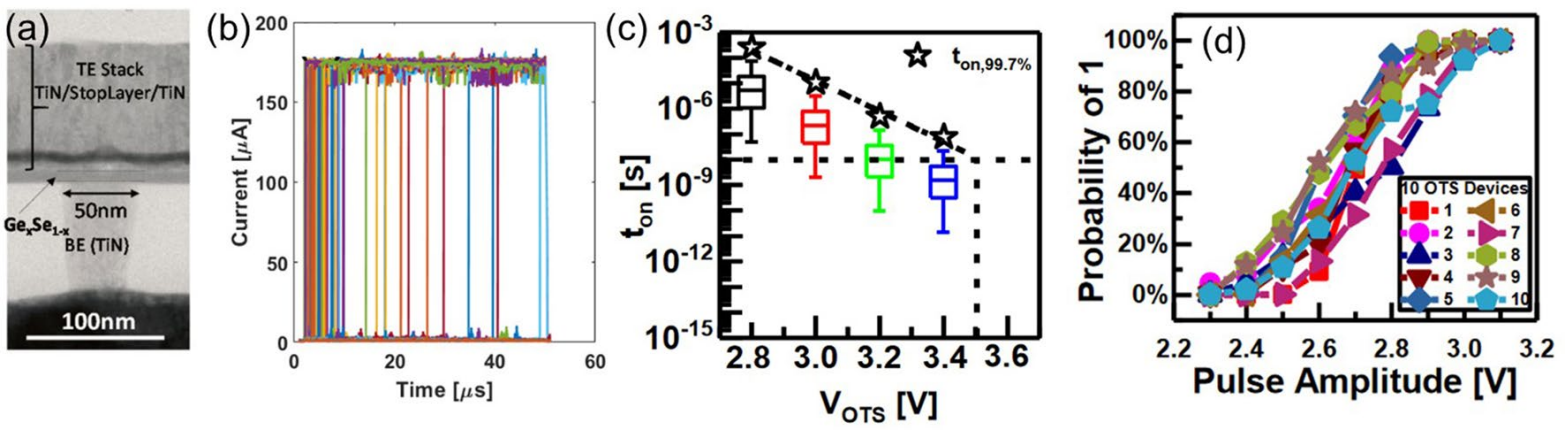
Ge–Se-based true random number generator. **a** Device structure with 50-nm plug. **b** Distribution of *t*_delay-on_ at 2.8 V. **c** Distribution of *t*_delay-on_ at different biases. **d** Occurrence rate of “1” at different pulse amplitudes measured in 10 devices [196]. Adapted with permission from [196]. Copyright 2021, IEEE

In addition, random number generation is another vibrant application area, with significant importance in disciplines such as communication, computer simulation, and cryptography. Random numbers generated using the inherent stochastic properties of physical devices possess true unpredictability and non-repeatability [190, 191]. Hence, the development of true random number generators (TRNGs) based on device characteristics at the hardware level has become a prominent research focus [192–195]. Chai et al. conducted a study on TRNGs constructed using Ge–Se-based OTS device. The research revealed that the delay time (on/off) at a constant voltage follows the Weibull distribution (Fig. 35b), enabling the extraction and extrapolation of the switching probability dependence on pulse waveform, bias, and time (Fig. 35c). This feature facilitated parameter quantization and yielded highly random results. The generated 10^4^-bit sequence successfully passed 12 tests in the NIST statistical test suite. Compared to RNGs composed of traditional non-volatile memories, the TRNG based on Ge–Se OTS devices exhibited a faster generation speed [196].

The volatile nature of OTS devices makes them well-suited for constructing non-weight storage elements in neuromorphic devices. Their typical switching behaviors have been predominantly utilized in the construction of ReLU functions for processing frequency-encoded data in current research. It should be noted that, at present, the training of weights often relies on preprocessing and input from external systems rather than being directly integrated within the OTS devices. While there are still many challenges to be addressed, OTS devices, with their favorable electrical performance and excellent scalability, hold promise for playing an important role in future neuromorphic computing.

## References

[1] D. Reinsel, J. Gantz, J. Rydning, *Data Age 2025: The Digitization of the World: From Edge to Core* (International Data Corporation (IDC), 2018). https://www.seagate.com/files/www-content/our-story/trends/files/idc-seagate-dataage-whitepaper.pdf.

[2] G.W. Burr, R.S. Shenoy, K. Virwani, P. Narayanan, A. Padilla et al., Access devices for 3D crosspoint memory. J. Vac. Sci. Technol. B **32**(4), 040802 (2014). 10.1116/1.4889999

[3] Q. Xia, J.J. Yang, Memristive crossbar arrays for brain-inspired computing. Nat. Mater. **18**, 309–323 (2019). 10.1038/s41563-019-0291-x 30894760 10.1038/s41563-019-0291-x

[4] R. Calarco, F. Arciprete, Keep it simple and switch to pure tellurium. Science **374**, 1321 (2021). 10.1126/science.abm7316 34882479 10.1126/science.abm7316

[5] M. Si, H.-Y. Cheng, T. Ando, G. Hu, P.D. Ye, Overview and outlook of emerging non-volatile memories. MRS Bull. **46**, 946–958 (2021). 10.1557/s43577-021-00204-2

[6] G. Servalli, A 45nm generation Phase Change Memory technology. in *2009 IEEE International Electron Devices Meeting (IEDM)*. Baltimore, MD, USA. 1–4 (2009). 10.1109/IEDM.2009.5424409

[7] G.W. Burr, K. Virwani, R.S. Shenoy, A. Padilla, M. BrightSky et al., Large-scale (512kbit) integration of multilayer-ready access-devices based on mixed-ionic-electronic-conduction (MIEC) at 100% yield. in *2012 Symposium on VLSI Technology (VLSIT)*. Honolulu, HI, USA. 41–42 (2012). 10.1109/VLSIT.2012.6242451

[8] K.L. Chopra, Current-controlled negative resistance in thin niobium oxide films. Proc. IEEE **51**, 941–942 (1963). 10.1109/PROC.1963.2339

[9] S. Kumar, J.P. Strachan, R.S. Williams, Chaotic dynamics in nanoscale NbO_2_ Mott memristors for analogue computing. Nature **548**, 318–321 (2017). 10.1038/nature23307 28792931 10.1038/nature23307

[10] R. Midya, Z. Wang, J. Zhang, S.E. Savel’ev, C. Li et al., Anatomy of Ag/*Hafnia*-based selectors with 10^10^ nonlinearity. Adv. Mater. **29**, 1604457 (2017). 10.1002/adma.201604457 10.1002/adma.201604457 28134458

[11] D. Kau, S. Tang, I.V. Karpov, R. Dodge, B. Klehn et al., A stackable cross point phase change memory. in *2009 IEEE International Electron Devices Meeting (IEDM)*. Baltimore, MD, USA. 1–4 (2009). 10.1109/IEDM.2009.5424263

[12] A. Fazio, Advanced technology and systems of cross point memory. in *2020 IEEE International Electron Devices Meeting (IEDM)*. San Francisco, CA, USA. 24.1.1–24.1.4 (2020). 10.1109/IEDM13553.2020.9371976

[13] W.R. Northover, A.D. Pearson, US Patent 3117013 (1964).

[14] R.H. Dennard, US Patent 3387286 (1967).

[15] S.R. Ovshinsky, US Patent 3271591 (1966).

[16] S.R. Ovshinsky, Reversible electrical switching phenomena in disordered structures. Phys. Rev. Lett. **21**, 1450–1453 (1968). 10.1103/physrevlett.21.1450

[17] D.L. Nelson, Ovonic device applications. J. Non Cryst. Solids **2**, 528–539 (1970). 10.1016/0022-3093(70)90166-3

[18] N. Yamada, E. Ohno, K. Nishiuchi, N. Akahira, M. Takao, Rapid-phase transitions of GeTe-Sb_2_Te_3_ pseudobinary amorphous thin films for an optical disk memory. J. Appl. Phys. **69**, 2849–2856 (1991). 10.1063/1.348620

[19] M. Zhu, K. Ren, L. Liu, S. Lv, X. Miao et al., Direct observation of partial disorder and zipperlike transition in crystalline phase change materials. Phys. Rev. Mater. **3**, 033603 (2019). 10.1103/physrevmaterials.3.033603

[20] H. Iwasaki, Y. Ide, M. Harigaya, Y. Kageyama, I. Fujimura, Completely erasable phase change optical disk. Jpn. J. Appl. Phys. **31**, 461 (1992). 10.1143/jjap.31.461

[21] M. Zhu, W. Song, P.M. Konze, T. Li, B. Gault et al., Direct atomic insight into the role of dopants in phase-change materials. Nat. Commun. **10**, 3525 (2019). 10.1038/s41467-019-11506-0 31388013 10.1038/s41467-019-11506-0 PMC6684653

[22] E.J. Evans, J.H. Helbers, S.R. Ovshinsky, Reversible conductivity transformations in chalcogenide alloy films. J. Non Cryst. Solids **2**, 334–346 (1970). 10.1016/0022-3093(70)90149-3

[23] S. Lai, T. Lowrey, OUM—A 180 nm nonvolatile memory cell element technology for stand alone and embedded applications. in *International Electron Devices Meeting. Technical Digest* (Cat. No.01CH37224). Washington, DC, USA. 36.5.1–36.5.4 (2001). 10.1109/IEDM.2001.979636

[24] M. Gill, T. Lowrey, J. Park, Ovonic unified memory—a high-performance nonvolatile memory technology for stand-alone memory and embedded applications. in *2002 IEEE International Solid-State Circuits Conference. Digest of Technical Papers* (Cat. No.02CH37315). San Francisco, CA, USA. 202–459 (2002). 10.1109/ISSCC.2002.993006

[25] F. Bedeschi, C. Resta, O. Khouri, B. E., L. Costa et al., An 8Mb demonstrator for high-density 1.8V Phase-Change Memories. in *2004 Symposium on VLSI Circuits. Digest of Technical Papers*. Honolulu, HI, USA. 442–445 (2004). 10.1109/VLSIC.2004.1346644

[26] M.J. Kang, T.J. Park, Y.W. Kwon, D.H. Ahn, Y.S. Kang et al., PRAM cell technology and characterization in 20 nm node size. in *2011 International Electron Devices Meeting*. Washington, DC, USA. 3.1.1–3.1.4 (2011). 10.1109/IEDM.2011.6131478

[27] J.H. Oh, J.H. Park, Y.S. Lim, H.S. Lim, Y.T. Oh et al., Full integration of highly manufacturable 512 Mb PRAM based on 90 nm technology. in *2006 International Electron Devices Meeting*. San Francisco, CA, USA. 1–4 (2006). 10.1109/IEDM.2006.346905

[28] M. Zhu, K. Ren, Z. Song, Ovonic threshold switching selectors for three-dimensional stackable phase-change memory. MRS Bull. **44**, 715–720 (2019). 10.1557/mrs.2019.206

[29] Y. Choi, I. Song, M.-H. Park, H. Chung, S. Chang et al., A 20nm 1.8 V 8 Gb PRAM with 40MB/s program bandwidth. in *2012 IEEE International Solid-State Circuits Conference*. San Francisco, CA, USA. 46–48 (2012). 10.1109/ISSCC.2012.6176872

[30] J. Park, J. Yoo, J. Song, C. Sung, H. Hwang, Hybrid selector with excellent selectivity and fast switching speed for X-point memory array. IEEE Electron Device Lett. **39**, 1171–1174 (2018). 10.1109/LED.2018.2845878

[31] A. Verdy, G. Navarro, V. Sousa, P. Noe, M. Bernard et al., Improved electrical performance thanks to Sb and N doping in Se-rich GeSe-based OTS selector devices. in *2017 IEEE International Memory Workshop (IMW)*. Monterey, CA, USA. 1–4 (2017). 10.1109/IMW.2017.7939088

[32] N.S. Avasarala, B. Govoreanu, K. Opsomer, W. Devulder, S. Clima et al., Doped GeSe materials for selector applications. in *2017 47th European Solid-State Device Research Conference (ESSDERC)*. Leuven, Belgium. 168–171 (2017). 10.1109/ESSDERC.2017.8066618

[33] H.-S.P. Wong, H.-Y. Lee, S. Yu, Y.-S. Chen, Y. Wu et al., Metal–oxide RRAM. Proc. IEEE **100**, 1951–1970 (2012). 10.1109/JPROC.2012.2190369

[34] E. Ambrosi, C.-H. Wu, H.-Y. Lee, C.-M. Lee, C.-F. Hsu et al., Reliable low voltage selector device technology based on robust SiNGeCTe arsenic-free chalcogenide. IEEE Electron Device Lett. **43**, 1673–1676 (2022). 10.1109/LED.2022.3203146

[35] C.H. Wu, C.M. Lee, Y.S. Chen, H.Y. Lee, E. Ambrosi et al., Low-voltage (~ 1.3V), arsenic free threshold type selector with ultra high endurance (> 10^11^) for high density 1S1R memory array. in *2021 Symposium on VLSI Technology*. Kyoto, Japan. 1–2 (2021). https://ieeexplore.ieee.org/document/9508722

[36] L. Wang, J. Wen, Z. Liu, J. Chen, H. Tong et al., Thermally stable and high-speed Ge-Te based ovonic threshold switching selector with a Ge intercalated structure. IEEE Electron Device Lett. **44**, 1096–1099 (2023). 10.1109/LED.2023.3272884

[37] L. Wang, Z. Liu, Z. Zhang, J. Chen, J. Wen et al., A refresh operation method for solving thermal stability issues and improving endurance of ovonic threshold switching selectors. J. Mater. Chem. C **11**, 5411–5421 (2023). 10.1039/D3TC00448A

[38] N.S. Avasarala, G.L. Donadio, T. Witters, K. Opsomer, B. Govoreanu et al., Half-threshold bias I_off_ reduction down to nA range of thermally and electrically stable high-performance integrated OTS selector, obtained by Se enrichment and N-doping of thin GeSe layers. in *2018 IEEE Symposium on VLSI Technology*. Honolulu, HI, USA. 209–210 (2018). 10.1109/VLSIT.2018.8510680

[39] S.-D. Kim, H.-W. Ahn, S.Y. Shin, D.S. Jeong, S.H. Son et al., Effect of Ge concentration in Ge_*x*_Se_1-__*x*_ chalcogenide glass on the electronic structures and the characteristics of ovonic threshold switching (OTS) devices. ECS Solid State Lett. **2**, Q75–Q77 (2013). 10.1149/2.001310ssl

[40] S. Jia, N. Shi, J. Shen, R. Wu, Q. Liu et al., Valence band structure of chalcogenide obtained by X-ray photoelectron spectroscopy and etching technique. Phys. Status Solidi B **258**(7), 2100038 (2021). 10.1002/pssb.202100038

[41] M. Xu, M. Xu, X. Miao, Deep machine learning unravels the structural origin of mid-gap states in chalcogenide glass for high-density memory integration. InfoMat **4**, e12315 (2022). 10.1002/inf2.12315

[42] A. Verdy, G. Navarro, M. Bernard, S. Chevalliez, N. Castellani et al., Carbon electrode for Ge-Se-Sb based OTS selector for ultra low leakage current and outstanding endurance. in *2018 IEEE International Reliability Physics Symposium (IRPS)*. 6D.4–1–6D.4–6 (2018). 10.1109/IRPS.2018.8353635

[43] X. Li, Z. Yuan, S. Lv, S. Song, Z. Song, Extended endurance performance and reduced threshold voltage by doping Si in GeSe-based ovonic threshold switching selectors. Thin Solid Films **734**, 138837 (2021). 10.1016/j.tsf.2021.138837

[44] E.P. O’Reilly, J. Robertson, M.J. Kelly, The structure of amorphous GeSe and GeTe. Solid State Commun. **38**, 565–568 (1981). 10.1016/0038-1098(81)90941-8

[45] G.I. Kim, J. Shirafuji, Time of flight measurement of carrier mobility in Ge_*x*_Se_1-__*x*_ glasses. Jpn. J. Appl. Phys. **17**, 1789–1794 (1978). 10.1143/jjap.17.1789

[46] T.T. Nang, M. Okuda, T. Matsushita, S. Yokota, A. Suzuki, Electrical and optical properties of Ge_*x*_Se_1-__*x*_ amorphous thin films. Jpn. J. Appl. Phys. **15**, 849–853 (1976). 10.1143/jjap.15.849

[47] O. Uemura, Y. Sagara, T. Satow, The amorphous structure of the Ge-Se system. Phys. Stat. Sol. **26**(1), 99–103 (1974). 10.1002/pssa.2210260108

[48] S.Y. Shin, J.M. Choi, J. Seo, H.W. Ahn, Y.G. Choi et al., The effect of doping Sb on the electronic structure and the device characteristics of Ovonic Threshold Switches based on Ge-Se. Sci. Rep. **4**, 7099 (2014). 10.1038/srep07099 25403772 10.1038/srep07099 PMC4235286

[49] J. Keukelier, K. Opsomer, W. Devulder, S. Clima, L. Goux et al., Tuning of the thermal stability and ovonic threshold switching properties of GeSe with metallic and non-metallic alloying elements. J. Appl. Phys. **130**(16), 165103 (2021). 10.1063/5.0055861

[50] H.Y. Cheng, W.C. Chien, I.T. Kuo, C.W. Yeh, L. Gignac et al., Ultra-high endurance and low I_OFF_ selector based on AsSeGe chalcogenides for wide memory window 3D stackable crosspoint memory. in *2018 IEEE International Electron Devices Meeting (IEDM)*. San Francisco, CA, USA. 37.3.1–37.3.4 (2018). 10.1109/IEDM.2018.8614580

[51] H.Y. Cheng, W.C. Chien, I.T. Kuo, E.K. Lai, Y. Zhu et al., An ultra high endurance and thermally stable selector based on TeAsGeSiSe chalcogenides compatible with BEOL IC Integration for cross-point PCM. in *2017 IEEE International Electron Devices Meeting (IEDM)*. San Francisco, CA, USA. 2.2.1–2.2.4 (2017). 10.1109/IEDM.2017.8268310

[52] H.Y. Cheng, W.C. Chien, I.T. Kuo, C.H. Yang, Y.C. Chou et al., Optimizing AsSeGe chalcogenides by dopants for extremely low I_OFF_, high endurance and low V_th_ drift 3D crosspoint memory. in *2021 IEEE International Electron Devices Meeting (IEDM)*. San Francisco, CA, USA. 28.6.1–28.6.4 (2021). 10.1109/IEDM19574.2021.9720704

[53] S. Jun, S. Seo, S. Park, T.H. Kim, M. Lee et al., Amorphous Ge-Se-S chalcogenide alloys via post plasma sulfurization of atomic layer deposition GeSe for ovonic threshold switch applications. J. Alloys Compd. **947**, 169514 (2023). 10.1016/j.jallcom.2023.169514

[54] B. Govoreanu, G.L. Donadio, K. Opsomer, W. Devulder, V.V. Afanas’ev et al., Thermally stable integrated Se-based OTS selectors with > 20 MA/cm^2^ current drive, > 3.10^3^ half-bias nonlinearity, tunable threshold voltage and excellent endurance. in *2017 Symposium on VLSI Technology*. Kyoto, Japan. T92–T93 (2017). 10.23919/VLSIT.2017.7998207

[55] W.-C. Chien, C.-W. Yeh, R.L. Bruce, H.-Y. Cheng, I.T. Kuo et al., A study on OTS-PCM pillar cell for 3-D stackable memory. IEEE Trans. Electron Devices **65**, 5172–5179 (2018). 10.1109/TED.2018.2871197

[56] M. Chen, K.A. Rubin, R.W. Barton, Compound materials for reversible, phase-change optical data storage. Appl. Phys. Lett. **49**, 502–504 (1986). 10.1063/1.97617

[57] A. Deneuville, J.P. Keradec, P. Gerard, A. Mini, DC electrical, optical and photoelectrical properties of Ge_x_Te_1−x_ amorphous thin films. Solid State Commun. **14**, 341–346 (1974). 10.1016/0038-1098(74)90914-4

[58] B. Kramer, K. Maschke, L.D. Laude, Electronic spectra of trigonal and disordered phases of tellurium and selenium. I. Theory. Phys. Rev. B **8**, 5781–5793 (1973). 10.1103/physrevb.8.5781

[59] F.A. Blum, B.C. Deaton, Properties of the group VI B elements under pressure. II. semiconductor-to-metal transition of tellurium. Phys. Rev. **137**, A1410–A1417 (1965). 10.1103/physrev.137.a1410

[60] J. Shen, S. Jia, N. Shi, Q. Ge, T. Gotoh et al., Elemental electrical switch enabling phase segregation-free operation. Science **374**, 1390–1394 (2021). 10.1126/science.abi6332 34882462 10.1126/science.abi6332

[61] C. Kim, N. Hur, J. Yang, S. Oh, J. Yeo et al., Atomic layer deposition route to scalable, electronic-grade van der waals Te thin films. ACS Nano **17**, 15776–15786 (2023). 10.1021/acsnano.3c03559 37432767 10.1021/acsnano.3c03559

[62] Y. Koo, H. Hwang, Zn_1-__*x*_Te_*x*_ ovonic threshold switching device performance and its correlation to material parameters. Sci. Rep. **8**, 11822 (2018). 10.1038/s41598-018-30207-0 30087380 10.1038/s41598-018-30207-0 PMC6081415

[63] S. Lee, J. Lee, S. Kim, D. Lee, D. Lee et al., Mg-Te OTS selector with low i_off_ (<100pA), Fast Switching Speed (τd = 7 ns), and high thermal stability (400 ℃/30 min) for X-point memory applications. in *2021 Symposium on VLSI Technology*. Kyoto, Japan. 1–2 (2021). https://ieeexplore.ieee.org/document/9508648

[64] M. Anbarasu, M. Wimmer, G. Bruns, M. Salinga, M. Wuttig, Nanosecond threshold switching of GeTe_6_ cells and their potential as selector devices. Appl. Phys. Lett. **100**(14), 143505 (2012). 10.1063/1.3700743

[65] A. Velea, K. Opsomer, W. Devulder, J. Dumortier, J. Fan et al., Te-based chalcogenide materials for selector applications. Sci. Rep. **7**, 8103 (2017). 10.1038/s41598-017-08251-z 28808294 10.1038/s41598-017-08251-z PMC5556072

[66] S. Jia, H. Li, T. Gotoh, C. Longeaud, B. Zhang et al., Ultrahigh drive current and large selectivity in GeS selector. Nat. Commun. **11**, 4636 (2020). 10.1038/s41467-020-18382-z 32934210 10.1038/s41467-020-18382-z PMC7493911

[67] M. Anbarasu, S. Asokan, Low electric field, easily reversible electrical *set* and *reset* processes in a Ge_15_Te_83_Si_2_ glass for phase change memory applications. J. Appl. Phys. **109**(8), 084517 (2011). 10.1063/1.3574659

[68] C. Mihai, F. Sava, I.D. Simandan, A.C. Galca, I. Burducea et al., Structural and optical properties of amorphous Si-Ge-Te thin films prepared by combinatorial sputtering. Sci. Rep. **11**, 11755 (2021). 10.1038/s41598-021-91138-x 34083613 10.1038/s41598-021-91138-x PMC8175571

[69] L. Wang, W. Cai, D. He, Q. Lin, D. Wan et al., Performance improvement of GeTe_x_-based ovonic threshold switching selector by C doping. IEEE Electron Device Lett. **42**, 688–691 (2021). 10.1109/LED.2021.3064857

[70] E. Ambrosi, C.H. Wu, H.Y. Lee, P.C. Chang, C.F. Hsu et al., Low variability high endurance and low voltage arsenic-free selectors based on GeCTe. in *2021 IEEE International Electron Devices Meeting (IEDM)*. San Francisco, CA, USA. 28.5.1–28.5.4 (2021). 10.1109/IEDM19574.2021.9720628

[71] E. Ambrosi, C.H. Wu, H.Y. Lee, C.F. Hsu, C.M. Lee et al., Engineering defects in pristine amorphous chalcogenides for forming-free low voltage selectors. in *2022 International Electron Devices Meeting (IEDM)*. San Francisco, CA, USA. 18.7.1–18.7.4 (2022). 10.1109/IEDM45625.2022.10019553

[72] D. Garbin, W. Devulder, R. Degraeve, G.L. Donadio, S. Clima et al., Composition optimization and device understanding of Si-Ge-As-Te ovonic threshold switch selector with excellent endurance. in *2019 IEEE International Electron Devices Meeting (IEDM)*. San Francisco, CA, USA. 35.1.1–35.1.4 (2019). 10.1109/IEDM19573.2019.8993547

[73] M.-J. Lee, D. Lee, H. Kim, H.-S. Choi, J.-B. Park et al., Highly-scalable threshold switching select device based on chaclogenide glasses for 3D nanoscaled memory arrays. in *2012 International Electron Devices Meeting*. San Francisco, CA, USA. 2.6.1–2.6.3 (2012). 10.1109/IEDM.2012.6478966

[74] O. Kazuhiro and H. Kanagawa, US Patent 20160336378A (2015).

[75] Y. Koo, K. Baek, H. Hwang, Te-based amorphous binary OTS device with excellent selector characteristics for x-point memory applications. in *2016 IEEE Symposium on VLSI Technology*. Honolulu, HI, USA. 1–2 (2016).

[76] J. Yoo, Y. Koo, S.A. Chekol, J. Park, J. Song et al., Te-based binary OTS selectors with excellent selectivity (> 10^5^), endurance (> 10^8^) and thermal stability (> 450 ℃). in *2018 IEEE Symposium on VLSI Technology*. Honolulu, HI, USA. 207–208 (2018). 10.1109/VLSIT.2018.8510681

[77] S.A. Chekol, J. Yoo, J. Park, J. Song, C. Sung et al., A C-Te-based binary OTS device exhibiting excellent performance and high thermal stability for selector application. Nanotechnology **29**, 345202 (2018). 10.1088/1361-6528/aac9f5 29863485 10.1088/1361-6528/aac9f5

[78] J. Yoo, D. Lee, J. Park, J. Song, H. Hwang, Steep slope field-effect transistors with B-Te-based ovonic threshold switch device. IEEE J. Electron Devices Soc. **6**, 821–824 (2018). 10.1109/JEDS.2018.2856853

[79] T. Kim, D. Lee, J. Kim, H. Sohn, Firing voltage reduction in thermally annealed Ge–As–Te thin film with ovonic threshold switching. J. Vac. Sci. Technol. B **38**(3), 032213 (2020). 10.1116/1.5144736

[80] M.J. Lee, D. Lee, S.H. Cho, J.H. Hur, S.M. Lee et al., A plasma-treated chalcogenide switch device for stackable scalable 3D nanoscale memory. Nat. Commun. **4**, 2629 (2013). 10.1038/ncomms3629 24129660 10.1038/ncomms3629

[81] E. Zhu, Y. Liu, X. Sun, G. Yin, Q. Jiao et al., Correlation between thermo-mechanical properties and network structure in Ge_*x*_S_100–__*x*_ chalcogenide glasses. J. Non Cryst. Solids X **1**, 100015 (2019). 10.1016/j.nocx.2019.100015

[82] C.A. Mead, Energy gap in sulphur. Phys. Lett. **11**, 212–213 (1964). 10.1016/0031-9163(64)90410-x

[83] J. Málek, L. Tichý, J. Klikorka, Crystallization kinetics of Ge_*x*_S_1−__*x*_ glasses. J. Therm. Anal. **33**, 667–672 (1988). 10.1007/BF02138571

[84] K. Tanaka, Y. Kasanuki, A. Odajima, Physical properties and photoinduced changes of amorphous Ge–S films. Thin Solid Films **117**, 251–260 (1984). 10.1016/0040-6090(84)90355-9

[85] Z. Černošek, E. Černošková, M. Frumar, K. Swiatek, A non-dangling bond model of paramagnetic defects in Ge-S glasses. Phys. Status Solidi B **192**(1), 181–192 (1995). 10.1002/pssb.2221920121

[86] D.D. Vaughn 2nd., R.J. Patel, M.A. Hickner, R.E. Schaak, Single-crystal colloidal nanosheets of GeS and GeSe. J. Am. Chem. Soc. **132**, 15170–15172 (2010). 10.1021/ja107520b 20942394 10.1021/ja107520b

[87] A. Bytchkov, G.J. Cuello, S. Kohara, C.J. Benmore, D.L. Price et al., Unraveling the atomic structure of Ge-rich sulfide glasses. Phys. Chem. Chem. Phys. **15**, 8487–8494 (2013). 10.1039/c3cp50536g 23615750 10.1039/c3cp50536g

[88] S. Chakraborty, P. Boolchand, Topological origin of fragility, network adaptation, and rigidity and stress transitions in especially homogenized nonstoichiometric binary Ge_*x*_S_*100–x*_ glasses. J. Phys. Chem. B **118**, 2249–2263 (2014). 10.1021/jp411823j 24471439 10.1021/jp411823j

[89] A. Bouzid, S. Le Roux, G. Ori, M. Boero, C. Massobrio, Origin of structural analogies and differences between the atomic structures of GeSe_4_ and GeS_4_ glasses: a first principles study. J. Chem. Phys. **143**, 034504 (2015). 10.1063/1.4926830 26203033 10.1063/1.4926830

[90] H. Kan, Electronic structure of amorphous germanium sulphides. J. Non Cryst. Solids **312–314**, 566–569 (2002). 10.1016/s0022-3093(02)01781-7

[91] D. Foix, H. Martinez, A. Pradel, M. Ribes, D. Gonbeau, XPS valence band spectra and theoretical calculations for investigations on thiogermanate and thiosilicate glasses. Chem. Phys. **323**, 606–616 (2006). 10.1016/j.chemphys.2005.10.037

[92] R.R. Romanyuk, Charge carrier transfer in amorphous (GeS)_1-x_Bi_x_ films. Chem. Met. Alloys **6**, 200–204 (2013). 10.30970/cma6.0272

[93] Y. Chen, R. Wang, X. Shen, J. Wang, T. Xu, New methods versus old questions: crystallization kinetics of S, Se, and Te. Cryst. Growth Des. **19**, 1103–1110 (2019). 10.1021/acs.cgd.8b01608

[94] M. Kim, Y. Kim, M. Lee, S.M. Hong, H.K. Kim et al., PE-ALD of Ge_1−__*x*_S_*x*_ amorphous chalcogenide alloys for OTS applications. J. Mater. Chem. C **9**, 6006–6013 (2021). 10.1039/D1TC00650A

[95] S. Jia, H. Li, Q. Liu, Z. Song, M. Zhu, Scalability of sulfur-based ovonic threshold selectors for 3D stackable memory applications. Phys. Status Solidi RRL **15**(6), 2100084 (2021). 10.1002/pssr.202100084

[96] R. Wu, R. Gu, T. Gotoh, Z. Zhao, Y. Sun et al., The role of arsenic in the operation of sulfur-based electrical threshold switches. Nat. Commun. **14**, 6095 (2023). 10.1038/s41467-023-41643-6 37773231 10.1038/s41467-023-41643-6 PMC10542328

[97] M. Lee, S. Lee, M. Kim, S. Lee, C. Won et al., Ge_1–__*x*_S_x_ chalcogenide alloys for OTS applications using magnetron sputtering. J. Alloys Compd. **930**, 167409 (2023). 10.1016/j.jallcom.2022.167409

[98] D. Matsubayashi, S. Clima, T. Ravsher, D. Garbin, R. Delhougne et al., OTS physics-based screening for environment-friendly selector materials. in *2022 International Electron Devices Meeting (IEDM)*. San Francisco, CA, USA. 8.6.1–8.6.4 (2022). 10.1109/IEDM45625.2022.10019445

[99] S. Clima, D. Matsubayashi, T. Ravsher, D. Garbin, R. Delhougne et al., In silico screening for As/Se-free ovonic threshold switching materials. npj Comput. Mater. **9**, 96 (2023). 10.1038/s41524-023-01043-2

[100] R. Wu, S. Jia, T. Gotoh, Q. Luo, Z. Song et al., Screening switching materials with low leakage current and high thermal stability for neuromorphic computing. Adv. Electron. Mater. **8**, 2200150 (2022). 10.1002/aelm.202200150

[101] D. Adler, M.S. Shur, M. Silver, S.R. Ovshinsky, Threshold switching in chalcogenide-glass thin films. J. Appl. Phys. **51**, 3289–3309 (1980). 10.1063/1.328036

[102] A. Redaelli, A. Pirovano, A. Benvenuti, A.L. Lacaita, Threshold switching and phase transition numerical models for phase change memory simulations. J. Appl. Phys. **103**(11), 111101 (2008). 10.1063/1.2931951

[103] D.M. Kroll, Theory of electrical instabilities of mixed electronic and thermal origin. II. Switching as a nucleation process. Phys. Rev. B **11**, 3814–3821 (1975). 10.1103/physrevb.11.3814

[104] D.M. Kroll, Theory of electrical instabilities of mixed electronic and thermal origin. Phys. Rev. B **9**, 1669–1706 (1974). 10.1103/physrevb.9.1669

[105] V.G. Karpov, Y.A. Kryukov, I.V. Karpov, M. Mitra, Field-induced nucleation in phase change memory. Phys. Rev. B **78**, 052201 (2008). 10.1103/physrevb.78.052201

[106] V.G. Karpov, Y.A. Kryukov, S.D. Savransky, I.V. Karpov, Nucleation switching in phase change memory. Appl. Phys. Lett. **90**(12), 123504 (2007). 10.1063/1.2715024

[107] I.V. Karpov, M. Mitra, D. Kau, G. Spadini, Y.A. Kryukov et al., Fundamental drift of parameters in chalcogenide phase change memory. J. Appl. Phys. **102**(12), 124503 (2007). 10.1063/1.2825650

[108] F. Buscemi, E. Piccinini, L. Vandelli, F. Nardi, A. Padovani et al., A HydroDynamic model for trap-assisted tunneling conduction in ovonic devices. IEEE Trans. Electron Devices **70**, 1808–1814 (2023). 10.1109/TED.2023.3242229

[109] N. Saxena, A. Manivannan, Ultrafast threshold switching dynamics in phase-change materials. Phys. Status Solidi RRL **16**(9), 2200101 (2022). 10.1002/pssr.202200101

[110] M. Nardone, M. Simon, I.V. Karpov, V.G. Karpov, Electrical conduction in chalcogenide glasses of phase change memory. J. Appl. Phys. **112**(7), 071101 (2012). 10.1063/1.4738746

[111] T. Kaplan, D. Adler, Thermal effects in amorphous-semiconductor switching. Appl. Phys. Lett. **19**, 418–420 (1971). 10.1063/1.1653754

[112] T. Kaplan, D. Adler, Electrothermal switching in amorphous semiconductors. J. Non Cryst. Solids **8–10**, 538–543 (1972). 10.1016/0022-3093(72)90189-5

[113] T. Kaplan, D.C. Bullock, D. Adler, D.J. Epstein, Thermally induced negative resistance in Si-doped YIG. Appl. Phys. Lett. **20**, 439–441 (1972). 10.1063/1.1654007

[114] M. Wimmer, M. Salinga, The gradual nature of threshold switching. New J. Phys. **16**, 113044 (2014). 10.1088/1367-2630/16/11/113044

[115] A.E. Owen, J.M. Robertson, Electronic conduction and switching in chalcogenide glasses. IEEE Trans. Electron Devices **20**, 105–122 (1973). 10.1109/T-ED.1973.17617

[116] G.C. Vezzoli, P.J. Walsh, L.W. Doremus, Threshold switching and the on-state in non-crystalline chalcogenide semiconductors. J. Non Cryst. Solids **18**, 333–373 (1975). 10.1016/0022-3093(75)90138-6

[117] P. Fantini, A. Pirovano, D. Ventrice, A. Redaelli, Experimental investigation of transport properties in chalcogenide materials through 1∕f noise measurements. Appl. Phys. Lett. **88**(26), 263506 (2006). 10.1063/1.2215621

[118] D. Ielmini, Y. Zhang, Analytical model for subthreshold conduction and threshold switching in chalcogenide-based memory devices. J. Appl. Phys. **102**(5), 054517 (2007). 10.1063/1.2773688

[119] D. Ielmini, Threshold switching mechanism by high-field energy gain in the hopping transport of chalcogenide glasses. Phys. Rev. B **78**, 035308 (2008). 10.1103/physrevb.78.035308

[120] P. Fantini, N. Polino, A. Ghetti, D. Ielmini, Threshold switching by bipolar avalanche multiplication in ovonic chalcogenide glasses. Adv. Electron. Mater. **9**, 2300037 (2023). 10.1002/aelm.202300037

[121] M. Kastner, D. Adler, H. Fritzsche, Valence-alternation model for localized gap states in lone-pair semiconductors. Phys. Rev. Lett. **37**, 1504–1507 (1976). 10.1103/physrevlett.37.1504

[122] P.W. Anderson, Model for the electronic structure of amorphous semiconductors. Phys. Rev. Lett. **34**, 953–955 (1975). 10.1103/physrevlett.34.953

[123] R.A. Street, N.F. Mott, States in the gap in glassy semiconductors. Phys. Rev. Lett. **35**, 1293–1296 (1975). 10.1103/physrevlett.35.1293

[124] S. Clima, T. Ravsher, D. Garbin, R. Degraeve, A. Fantini et al., Ovonic threshold switch chalcogenides: connecting the first-principles electronic structure to selector device parameters. ACS Appl. Electron. Mater. **5**, 461–469 (2023). 10.1021/acsaelm.2c01458

[125] D. Emin, Current-driven threshold switching of a small polaron semiconductor to a metastable conductor. Phys. Rev. B **74**, 035206 (2006). 10.1103/physrevb.74.035206

[126] J. Luckas, Electronic transport in amorphous phase-change materials (Doctoral dissertation, Paris 11, 2012).

[127] R. Degraeve, T. Ravsher, S. Kabuyanagi, A. Fantini, S. Clima et al., Modeling and spectroscopy of ovonic threshold switching defects. in *2021 IEEE International Reliability Physics Symposium (IRPS)*. Monterey, CA, USA. 1–5 (2021). 10.1109/IRPS46558.2021.9405114

[128] N.F. Mott, Conduction in non-crystalline materials. Philos. Mag. **19**, 835–852 (1969). 10.1080/14786436908216338

[129] S. Clima, D. Garbin, K. Opsomer, N.S. Avasarala, W. Devulder et al., Ovonic threshold-switching Ge_*x*_Se_*y*_ chalcogenide materials: stoichiometry, trap nature, and material relaxation from first principles. Phys. Status Solidi RRL **14**(5), 1900672 (2020). 10.1002/pssr.201900672

[130] S. Clima, D. Garbin, W. Devulder, J. Keukelier, K. Opsomer et al., Material relaxation in chalcogenide OTS SELECTOR materials. Microelectron. Eng. **215**, 110996 (2019). 10.1016/j.mee.2019.110996

[131] S. Clima, B. Govoreanu, K. Opsomer, A. Velea, N.S. Avasarala et al., Atomistic investigation of the electronic structure, thermal properties and conduction defects in Ge-rich Ge_*x*_Se_1−*x*_ materials for selector applications. in *2017 IEEE International Electron Devices Meeting (IEDM)*. San Francisco, CA, USA. 4.1.1–4.1.4 (2017). 10.1109/IEDM.2017.8268323

[132] M. Luo, M. Wuttig, The dependence of crystal structure of Te-based phase-change materials on the number of valence electrons. Adv. Mater. **16**, 439–443 (2004). 10.1002/adma.200306077

[133] H. Li, J. Robertson, Materials selection and mechanism of non-linear conduction in chalcogenide selector devices. Sci. Rep. **9**, 1867 (2019). 10.1038/s41598-018-37717-x 30755641 10.1038/s41598-018-37717-x PMC6372668

[134] K. Konstantinou, F.C. Mocanu, J. Akola, S.R. Elliott, Electric-field-induced annihilation of localized gap defect states in amorphous phase-change memory materials. Acta Mater. **223**, 117465 (2022). 10.1016/j.actamat.2021.117465

[135] A.V. Kolobov, P. Fons, J. Tominaga, Understanding phase-change memory alloys from a chemical perspective. Sci. Rep. **5**, 13698 (2015). 10.1038/srep13698 26323962 10.1038/srep13698 PMC4555180

[136] P. Noé, A. Verdy, F. d’Acapito, J.-B. Dory, M. Bernard et al., Toward ultimate nonvolatile resistive memories: the mechanism behind ovonic threshold switching revealed. Sci. Adv. **6**, eaay2830 (2020). 10.1126/sciadv.aay2830 32158940 10.1126/sciadv.aay2830 PMC7048425

[137] J.-Y. Raty, P. Noé, Ovonic threshold switching in Se-rich Ge_*x*_Se_1–__*x*_ glasses from an atomistic point of view: the crucial role of the metavalent bonding mechanism. Phys. Status Solidi RRL **14**(5), 2070024 (2020). 10.1002/pssr.202070024

[138] D. Chandler, J.P. Garrahan, Dynamics on the way to forming glass: bubbles in space-time. Annu. Rev. Phys. Chem. **61**, 191–217 (2010). 10.1146/annurev.physchem.040808.090405 20055676 10.1146/annurev.physchem.040808.090405

[139] J.-Y. Raty, M. Schumacher, P. Golub, V.L. Deringer, C. Gatti et al., A quantum-mechanical map for bonding and properties in solids. Adv. Mater. **31**, e1806280 (2019). 10.1002/adma.201806280 30474156 10.1002/adma.201806280

[140] M. Zhu, O. Cojocaru-Mirédin, A.M. Mio, J. Keutgen, M. Küpers et al., Unique bond breaking in crystalline phase change materials and the quest for metavalent bonding. Adv. Mater. **30**, e1706735 (2018). 10.1002/adma.201706735 29572962 10.1002/adma.201706735

[141] K. Shportko, S. Kremers, M. Woda, D. Lencer, J. Robertson et al., Resonant bonding in crystalline phase-change materials. Nat. Mater. **7**, 653–658 (2008). 10.1038/nmat2226 18622406 10.1038/nmat2226

[142] A. Slassi, L.-S. Medondjio, A. Padovani, F. Tavanti, X. He et al., Device-to-materials pathway for electron traps detection in amorphous GeSe-based selectors. Adv. Electron. Mater. **9**, 2201224 (2023). 10.1002/aelm.202201224

[143] A. Pirovano, A.L. Lacaita, F. Pellizzer, S.A. Kostylev, A. Benvenuti et al., Low-field amorphous state resistance and threshold voltage drift in chalcogenide materials. IEEE Trans. Electron Devices **51**, 714–719 (2004). 10.1109/TED.2004.825805

[144] J.Y. Raty, W. Zhang, J. Luckas, C. Chen, R. Mazzarello et al., Aging mechanisms in amorphous phase-change materials. Nat. Commun. **6**, 7467 (2015). 10.1038/ncomms8467 26105012 10.1038/ncomms8467

[145] T. Ravsher, R. Degraeve, D. Garbin, A. Fantini, S. Clima et al., Polarity-dependent threshold voltage shift in ovonic threshold switches: challenges and opportunities. in *2021 IEEE International Electron Devices Meeting (IEDM)*. San Francisco, CA, USA. 28.4.1–28.4.4 (2021). 10.1109/IEDM19574.2021.9720649

[146] G.W. Burr, R.M. Shelby, A. Sebastian, S. Kim, S. Kim et al., Neuromorphic computing using non-volatile memory. Adv. Phys. X **2**, 89–124 (2017). 10.1080/23746149.2016.1259585

[147] Jeongdong Choe, *Memory Process, Design and Architecture: Today and Tomorrow* (TechInsights, 2017).

[148] J. H. Yoon, R. Godse, G. Tressler, H. Hunter, *3D-NAND Scaling & 3D-SCM-Implications to Enterprise Storage*. Flash Memory Summit, 2017.

[149] H.-Y. Cheng, F. Carta, W.-C. Chien, H.-L. Lung, M.J. BrightSky, 3D cross-point phase-change memory for storage-class memory. J. Phys. D Appl. Phys. **52**, 473002 (2019). 10.1088/1361-6463/ab39a0

[150] T. Kim, S. Lee, Evolution of phase-change memory for the storage-class memory and beyond. IEEE Trans. Electron Devices **67**, 1394–1406 (2020). 10.1109/ted.2020.2964640

[151] S. Hong, H. Choi, J. Park, Y. Bae, K. Kim et al., Extremely high performance, high density 20nm self-selecting cross-point memory for Compute Express Link. in *2022 International Electron Devices Meeting (IEDM)*. San Francisco, CA, USA. IEEE, 18.6.1–18.6.4 (2022). 10.1109/IEDM45625.2022.10019415

[152] C.W. Yeh, W.C. Chien, R.L. Bruce, H.Y. Cheng, I.T. Kuo et al., High endurance self-heating OTS-PCM pillar cell for 3D stackable memory. in *2018 IEEE Symposium on VLSI Technology*. Honolulu, HI, USA. 205–206 (2018). 10.1109/VLSIT.2018.8510621

[153] Y.K. Lee, C. Yoo, W. Kim, J.W. Jeon, C.S. Hwang, Atomic layer deposition of chalcogenides for next-generation phase change memory. J. Mater. Chem. C **9**, 3708–3725 (2021). 10.1039/D1TC00186H

[154] B. Govoreanu, D. Crotti, S. Subhechha, L. Zhang, Y.Y. Chen et al., A-VMCO: a novel forming-free, self-rectifying, analog memory cell with low-current operation, nonfilamentary switching and excellent variability. in *2015 Symposium on VLSI Technology (VLSI Technology)*. Kyoto, Japan. T132–T133, (2015). 10.1109/VLSIT.2015.7223717

[155] K.M. Kim, J. Zhang, C. Graves, J.J. Yang, B.J. Choi et al., Low-power, self-rectifying, and forming-free memristor with an asymmetric programing voltage for a high-density crossbar application. Nano Lett. **16**, 6724–6732 (2016). 10.1021/acs.nanolett.6b01781 27661260 10.1021/acs.nanolett.6b01781

[156] J.H. Yoon, S. Yoo, S.J. Song, K.J. Yoon, D.E. Kwon et al., Uniform self-rectifying resistive switching behavior via preformed conducting paths in a vertical-type Ta_2_O_5_/HfO_2-__*x*_ structure with a sub-μm^2^ cell area. ACS Appl. Mater. Interfaces **8**, 18215–18221 (2016). 10.1021/acsami.6b05657 27347693 10.1021/acsami.6b05657

[157] K. Jeon, J. Kim, J.J. Ryu, S.J. Yoo, C. Song et al., Self-rectifying resistive memory in passive crossbar arrays. Nat. Commun. **12**, 2968 (2021). 10.1038/s41467-021-23180-2 34016978 10.1038/s41467-021-23180-2 PMC8137934

[158] Y.-C. Chen, C.F. Chen, C.T. Chen, J.Y. Yu, S. Wu et al., An access-transistor-free (0T/1R) non-volatile resistance random access memory (RRAM) using a novel threshold switching, self-rectifying chalcogenide device. in *IEEE International Electron Devices Meeting*. Washington, DC, USA. 37.4.1–37.4.4 (2003). 10.1109/IEDM.2003.1269425

[159] J. Yoo, I. Karpov, S. Lee, J. Jung, H.S. Kim et al., Threshold voltage drift in Te-based ovonic threshold switch devices under various operation conditions. IEEE Electron Device Lett. **41**, 191–194 (2020). 10.1109/LED.2019.2957860

[160] Z. Chai, W. Zhang, R. Degraeve, S. Clima, F. Hatem et al., Dependence of switching probability on operation conditions in Ge_*x*_Se_1–__*x*_ ovonic threshold switching selectors. IEEE Electron Device Lett. **40**, 1269–1272 (2019). 10.1109/LED.2019.2924270

[161] S. Kabuyanagi, D. Garbin, A. Fantini, S. Clima, R. Degraeve et al., Understanding of tunable selector performance in Si-Ge-As-Se OTS devices by extended percolation cluster model considering operation scheme and material design. in *2020 IEEE Symposium on VLSI Technology*. Honolulu, HI, USA. 1–2 (2020). 10.1109/VLSITechnology18217.2020.9265011

[162] T. Ravsher, D. Garbin, A. Fantini, R. Degraeve, S. Clima et al., Enhanced performance and low-power capability of SiGeAsSe-GeSbTe 1S1R phase-change memory operated in bipolar mode. in *2022 IEEE Symposium on VLSI Technology and Circuits (VLSI Technology and Circuits)*. Honolulu, HI, USA. 312–313 (2022). 10.1109/VLSITechnologyandCir46769.2022.9830199

[163] T. Ravsher, D. Garbin, A. Fantini, R. Degraeve, S. Clima et al., Self-rectifying memory cell based on SiGeAsSe ovonic threshold switch. IEEE Trans. Electron Devices **70**, 2276–2281 (2023). 10.1109/TED.2023.3252491

[164] M.A. Zidan, J.P. Strachan, W.D. Lu, The future of electronics based on memristive systems. Nat. Electron. **1**, 22–29 (2018). 10.1038/s41928-017-0006-8

[165] T. Tuma, A. Pantazi, M. Le Gallo, A. Sebastian, E. Eleftheriou, Stochastic phase-change neurons. Nat. Nanotechnol. **11**, 693–699 (2016). 10.1038/nnano.2016.70 27183057 10.1038/nnano.2016.70

[166] M. Le Gallo, A. Sebastian, R. Mathis, M. Manica, H. Giefers et al., Mixed-precision in-memory computing. Nat. Electron. **1**, 246–253 (2018). 10.1038/s41928-018-0054-8

[167] S. Ambrogio, P. Narayanan, H. Tsai, R.M. Shelby, I. Boybat et al., Equivalent-accuracy accelerated neural-network training using analogue memory. Nature **558**, 60–67 (2018). 10.1038/s41586-018-0180-5 29875487 10.1038/s41586-018-0180-5

[168] V. Joshi, M. Le Gallo, S. Haefeli, I. Boybat, S.R. Nandakumar et al., Accurate deep neural network inference using computational phase-change memory. Nat. Commun. **11**, 2473 (2020). 10.1038/s41467-020-16108-9 32424184 10.1038/s41467-020-16108-9 PMC7235046

[169] D. Kuzum, R.G. Jeyasingh, B. Lee, H.S. Wong, Nanoelectronic programmable synapses based on phase change materials for brain-inspired computing. Nano Lett. **12**, 2179–2186 (2012). 10.1021/nl201040y 21668029 10.1021/nl201040y

[170] S.H. Jo, T. Chang, I. Ebong, B.B. Bhadviya, P. Mazumder et al., Nanoscale memristor device as synapse in neuromorphic systems. Nano Lett. **10**, 1297–1301 (2010). 10.1021/nl904092h 20192230 10.1021/nl904092h

[171] M. Di Ventra, Y.V. Pershin, The parallel approach. Nat. Phys. **9**, 200–202 (2013). 10.1038/nphys2566

[172] Z. Cheng, C. Ríos, W.H.P. Pernice, C.D. Wright, H. Bhaskaran, On-chip photonic synapse. Sci. Adv. **3**, e1700160 (2017). 10.1126/sciadv.1700160 28959725 10.1126/sciadv.1700160 PMC5617375

[173] S.B. Eryilmaz, D. Kuzum, S. Yu, H.-S P. Wong, Device and system level design considerations for analog-non-volatile-memory based neuromorphic architectures. in *2015 IEEE International Electron Devices Meeting (IEDM)*. Washington, DC, USA. 4.1.1–4.1.4 (2015). 10.1109/IEDM.2015.7409622

[174] K.K. Likharev, Hybrid CMOS/nanoelectronic circuits: opportunities and challenges. J. Nanoelectron. Optoelectron. **3**, 203–230 (2008). 10.1166/jno.2008.301

[175] W. Senn, S. Fusi, Convergence of stochastic learning in perceptrons with binary synapses. Phys. Rev. E Stat. Nonlinear Soft Matter Phys. **71**, 061907 (2005). 10.1103/PhysRevE.71.061907 10.1103/PhysRevE.71.061907 16089765

[176] Z. Wang, S. Joshi, S. Savel’ev, W. Song, R. Midya et al., Fully memristive neural networks for pattern classification with unsupervised learning. Nat. Electron. **1**, 137–145 (2018). 10.1038/s41928-018-0023-2

[177] D. Liang, G. Indiveri, A neuromorphic computational primitive for robust context-dependent decision making and context-dependent stochastic computation. IEEE Trans. Circuits Syst. II Express Briefs **66**, 843–847 (2019). 10.1109/TCSII.2019.2907848

[178] S. Hwang, J.-J. Lee, M.-W. Kwon, M.-H. Baek, T. Jang et al., Analog complementary metal-oxide-semiconductor integrate-and-fire neuron circuit for overflow retaining in hardware spiking neural networks. J. Nanosci. Nanotechnol. **20**, 3117–3122 (2020). 10.1166/jnn.2020.17390 31635655 10.1166/jnn.2020.17390

[179] B. Rueckauer, S.-C. Liu, Conversion of analog to spiking neural networks using sparse temporal coding. in *2018 IEEE International Symposium on Circuits and Systems (ISCAS)*. Florence, Italy. 1–5 (2018). 10.1109/ISCAS.2018.8351295

[180] H. Lim, H.-W. Ahn, V. Kornijcuk, G. Kim, J.Y. Seok et al., Relaxation oscillator-realized artificial electronic neurons, their responses, and noise. Nanoscale **8**, 9629–9640 (2016). 10.1039/C6NR01278G 27103542 10.1039/c6nr01278g

[181] W. Yi, K.K. Tsang, S.K. Lam, X. Bai, J.A. Crowell et al., Biological plausibility and stochasticity in scalable VO_2_ active memristor neurons. Nat. Commun. **9**, 466 (2018). 10.1038/s41467-018-07052-w 30405124 10.1038/s41467-018-07052-w PMC6220189

[182] M.D. Pickett, G. Medeiros-Ribeiro, R.S. Williams, A scalable neuristor built with Mott memristors. Nat. Mater. **12**, 114–117 (2013). 10.1038/nmat3510 23241533 10.1038/nmat3510

[183] X. Zhang, W. Wang, Q. Liu, X. Zhao, J. Wei et al., An artificial neuron based on a threshold switching memristor. IEEE Electron Device Lett. **39**, 308–311 (2018). 10.1109/LED.2017.2782752

[184] D. Lee, M. Kwak, K. Moon, W. Choi, J. Park et al., Various threshold switching devices for integrate and fire neuron applications. Adv. Electron. Mater. **5**, 1800866 (2019). 10.1002/aelm.201800866

[185] M. Lee, S.W. Cho, S.J. Kim, J.Y. Kwak, H. Ju et al., Simple artificial neuron using an ovonic threshold switch featuring spike-frequency adaptation and chaotic activity. Phys. Rev. Appl. **13**, 064056 (2020). 10.1103/physrevapplied.13.064056

[186] C. Zhang, W.B. Ye, K. Zhou, H.-Y. Chen, J.-Q. Yang et al., Artificial sensory nerves: bioinspired artificial sensory nerve based on nafion memristor. Adv. Funct. Mater. **29**, 1970133 (2019). 10.1002/adfm.201970133

[187] B.C. Tee, A. Chortos, A. Berndt, A.K. Nguyen, A. Tom et al., A skin-inspired organic digital mechanoreceptor. Science **350**, 313–316 (2015). 10.1126/science.aaa9306 26472906 10.1126/science.aaa9306

[188] X. Zhang, Y. Zhuo, Q. Luo, Z. Wu, R. Midya et al., An artificial spiking afferent nerve based on Mott memristors for neurorobotics. Nat. Commun. **11**, 51 (2020). 10.1038/s41467-019-13827-6 31896758 10.1038/s41467-019-13827-6 PMC6940364

[189] H. Lee, S.W. Cho, S.J. Kim, J. Lee, K.S. Kim et al., Three-terminal ovonic threshold switch (3T-OTS) with tunable threshold voltage for versatile artificial sensory neurons. Nano Lett. **22**, 733–739 (2022). 10.1021/acs.nanolett.1c04125 35025519 10.1021/acs.nanolett.1c04125

[190] A. Alaghi, J.P. Hayes, Survey of stochastic computing. ACM Trans. Embed. Comput. Syst. **12**(2), 92 (2013). 10.1145/2465787.2465794

[191] R. Pappu, B. Recht, J. Taylor, N. Gershenfeld, Physical one-way functions. Science **297**, 2026–2030 (2002). 10.1126/science.1074376 12242435 10.1126/science.1074376

[192] S. Balatti, S. Ambrogio, R. Carboni, V. Milo, Z. Wang et al., Physical unbiased generation of random numbers with coupled resistive switching devices. IEEE Trans. Electron Devices **63**, 2029–2035 (2016). 10.1109/TED.2016.2537792

[193] M. Le Gallo, T. Tuma, F. Zipoli, A. Sebastian, E. Eleftheriou, Inherent stochasticity in phase-change memory devices. in *2016 46th European Solid-State Device Research Conference (ESSDERC)*. Lausanne, Switzerland. 373–376 (2016). 10.1109/ESSDERC.2016.7599664

[194] S. Balatti, S. Ambrogio, Z. Wang, D. Ielmini, True random number generation by variability of resistive switching in oxide-based devices. IEEE J. Emerg. Sel. Top. Circuits Syst. **5**, 214–221 (2015). 10.1109/JETCAS.2015.2426492

[195] A. Fukushima, T. Seki, K. Yakushiji, H. Kubota, H. Imamura et al., Spin dice: a scalable truly random number generator based on spintronics. Appl. Phys. Express **7**, 083001 (2014). 10.7567/apex.7.083001

[196] Z. Chai, P. Freitas, W. Zhang, J.F. Zhang, J. Marsland, True random number generator based on switching probability of volatile Ge_*X*_Se_*1-X*_ ovonic threshold switching selectors. in *2021 IEEE 14th International Conference on ASIC (ASICON)*. Kunming, China. 1–4 (2021). 10.1109/ASICON52560.2021.9620374

